# Nine new cytochalasan alkaloids from Chaetomium globosum TW1-1 (Ascomycota, Sordariales)

**DOI:** 10.1038/srep18711

**Published:** 2016-01-07

**Authors:** Chunmei Chen, Qingyi Tong, Hucheng Zhu, Dongdong Tan, Jinwen Zhang, Yongbo Xue, Guangmin Yao, Zengwei Luo, Jianping Wang, Yanyan Wang, Yonghui Zhang

**Affiliations:** 1 Hubei Key Laboratory of Natural Medicinal Chemistry and Resource Evaluation, School of Pharmacy, Tongji Medical College, Huazhong University of Science and Technology, Wuhan 430030, China; 2 First College of Clinical Medical Science, China Three Gorges University & Yichang Central People’s Hospital, Yichang 443003, China; 3 Tongji Hospital Affliated to Tongji Medical College, Huazhong University of Science and Technology, Wuhan 430030, China

## Abstract

Chemical investigation on the methanol extract of *Chaetomium globosum* TW1-1, a fungus isolated from the common pillbug (*Armadillidium vulgare*), has resulted in the isolation of nine new highly oxygenated cytochalasan alkaloids, armochaetoglobins S–Z (**1** and **3**–**9**) and 7-*O*-acetylarmochaetoglobin S (**2**), together with eight structurally related known analogues (**10**–**17**). Their structures and absolute configurations were elucidated by spectroscopic analyses. Among them, compound **2** presents to be the first member of chaetoglobosin family with an acetyl group, and compounds **3** represents the first chaetoglobosin characterized by an 2′,3′-epoxy-indole moiety. The discovery of these new compounds revealed the largely untapped chemical diversity of cytochalasans and enriched their chemical research. Compounds **1**–**9** were evaluated for their cytotoxic activities against five human cancer cell lines, and compounds **8** and **9** exhibited significant cytotoxic activities with IC_50_ values ranging from 10.45 to 30.42 *μ*M.

Cytochalasans are an important class of natural products^1^,^2^, and they have attracted considerable attention from synthetic organic chemists^3^,^4^,^5^, biologists^6^,^7^,^8^, and phytochemists^9^,^10^,^11^,^12^,^13^ for decades because of their complex polycyclic skeletons and varied and often potent bioactivities, including antitumor^6^,^14^, anti-HIV^15^. and immunomodulatory activities^7^. Biogenetically, cytochalasans are biosynthesized by a large multi-domain enzymes of PKS-NRPS hybrid synthase and macrocyclization by means of a Diels-Alder reaction^16^,^17^. To date, more than 200 cytochalasans have been isolated and identified from many genera of ascomycete fungi^18^,^19^,^20^,^21^.

The genus *Chaetomium* has been reported to be a rich source of cytochalasans, with about one fourth of the naturally occurring cytochalasans were reported from this genus alone^10^,^12^,^22^,^23^. As part of our ongoing research on structurally interesting and biologically significant cytochalasans from fungi^24^,^25^,^26^,^27^, a culture of *Chaetomium globosum* TW1-1 was chemically investigated. As a result, nine new highly oxygenated cytochalasan alkaloids, armochaetoglobins S–Z (**1**, **3**, and **4–9**) and 7-*O*-acetylarmochaetoglobin S (**2**) (Fig. 1), together with eight structurally related known analogues (**10**–**17**), were obtained from the methanol extract of solid rice culture of *Chaetomium globosum* TW1-1. In this paper, the fermentation, isolation, and structure elucidation of these compounds are described.

## Results and Discussion

### Isolation and Structure Elucidation

The solid rice culture of *Chaetomium globosum* TW1-1 was extracted with methanol for five times at room temperature. The extract was subjected to series of chromatographic separation, including silica gel, RP-C_18_ (reversed-phase), Sephadex LH-20, and semi-preparative HPLC to yield nine new (**1**−**9**) and eight known cytochalasan alkaloids (**10**−**17**). The known compounds were identified as chaetoglobosins F (**10**)^28^, U (**11**)^20^, V (**12**)^29^, Y (**13**)^22^, cytoglobosin A (**14**)^30^, 20-dihydro-chaetoglobosin A (**15**)^31^, prochaetoglobosins I (**16**)^31^ and II (**17**)^31^ by comparing their NMR data with those reported in the literature.

**Figure 2:**
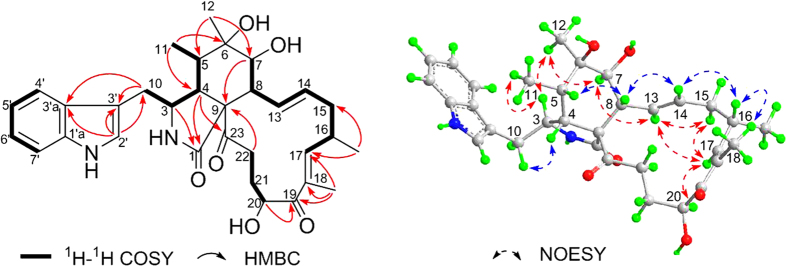
Key 2D NMR correlations for 1.

The molecular formula of **1** was established as C_32_H_40_N_2_O_6_ by HRESIMS at *m*/*z* 549.2936 ([M + H]^+^, cacld for C_32_H_41_N_2_O_6_, 549.2965), indicating 13 degrees of unsaturation. The ^13^C NMR and DEPT spectra of compound **1** displayed 32 carbons, including four methyls, four methylenes, fifteen methines, and nine quaternary carbons. The carbon profile and characteristic ^1^H NMR signals (Table 1), as well as 2D NMR spectra of **1** revealed that it has a similar indole based cytochalasan skeleton as that of chaetoglobosin Q^14^. Comparison of the NMR data (Tables 1 and 2) of **1** with those of chaetoglobosin Q suggested their structural similarities. The main difference of the two compounds is the fragment from C-19 to C-22 in the macrocycle ring. The olefinic carbons C-21 and C-22 in chaetoglobosin Q were replaced by two methylene signals in the spectra of **1**, which was suggested by ^1^H–^1^H COSY correlation of H-21/H-22 and HMBC cross-peaks from H-20 to C-19, C-21, and C-22. (Fig. 2) Additionally, chemical shifts of C-19 (*δ*_C_ 205.7) and C-20 (*δ*_C_ 72.4) combined with ^1^H–^1^H COSY cross-peak of H-20/H-21 and HMBC correlation from C-18-Me to C-19 indicated that the position of the carbonyl and the oxygenated methine were reversed comparing to chaetoglobosin Q.

**Figure 3:**
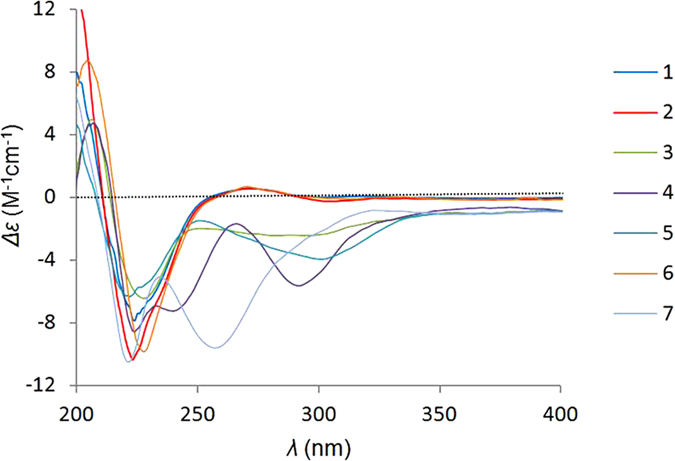
ECD curves for compounds 1−6.

The relative configuration of compound **1** was established by a NOESY experiment (Fig. 2) aided by comparing the chemical shifts and coupling constants of **1** with the known analogues. The strong NOESY correlation of H-5 and H-8 implied a boat conformation of the six-membered ring and both protons were ambiguously assigned as *β*-orientations. Consequently, NOESY correlations of Me-11 to H-3 and Me-12, and of Me-12 to H-7 revealed their co-facial and *α*-orientation. In addition, NOESY interaction of H-4 and H-10 suggested their *β*-orientations. The geometries of the double bonds (Δ^13^ and Δ^17^) in the 13-membered ring were determined to be *trans* (*E*) by the large coupling constant (*J* = 14.6 Hz) and NOESY correlation of H-16/18-Me, respectively. Furthermore, the *α*-orientation of 16-Me and *β*-orientation of 20-OH were determined by NOESY correlations from H-14 to H-8 and H-16, from H-17 to H-20 and H-15*α*, and from H-13 to H-15*α* and H-7. The absolute configuration of **1** was established by comparing the ECD spectrum (Fig. 3) of **1** with that of armochaetoglobin F^26^, whose absolute configuration was undoubtedly identified by X-Ray diffraction analysis. The final name of armochaetoglobin S was assigned to **1**.

The molecular weight of compound **2** was 42 mass units more than that of **1** as revealed by HRESIMS ion peak at *m*/*z* 613.2859 ([M + Na]^+^, cacld for C_34_H_42_N_2_O_7_Na, 613.2890). Comparison of the NMR data (Tables 1 and 2) for **2** and **1** revealed a high level of similarity with the only significant difference of the presence of an additional acetyl (*δ*_C_ 20.8 and 172.3; *δ*_H_ 1.98, 3H, s). The acetyl group was located at C-7 based on HMBC correlations from H-7 to C-1′′. Further analyses of 2D NMR data (^1^H–^1^H COSY, HMBC, and NOESY) of **2** confirmed the structure and relative configuration of **2**, and it was given the trivial name 7-*O*-acetylarmochaetoglobin S. To the best of our knowledge, compound **2** presents to be the first acetylated chaetoglobosin.

Compound **3** was obtained as colorless amorphous powder. The HRESIMS data showed a quasi-molecular ion peak at *m*/*z* 569.2628 ([M + Na]^+^), corresponding to a molecular formula of C_32_H_38_N_2_O_6_ with 15 degrees of unsaturation. Comparison of the NMR data (Tables 1 and 2) of **3** with those of chaetoglobosin F(**10**)^28^ suggested their structural similarities, however, with higher degrees of oxidation than that of **10**. The unexpected epoxy ring fused to the indole ring in compound **3** was evidenced by the chemical shifts of C-2′ (*δ*_C_ 82.4) and C-3′ (*δ*_C_ 91.9) and HMBC correlations from H-2′ to C-10, C-3′, C-1′a, and C-3′a. The overall NMR data, including HSQC, ^1^H–^1^H COSY, and HMBC spectra, allowed the elucidation of the planar structure of **3**. Compound **3** represents the first example of naturally occurring chaetoglobosin characterized by 2′,3′-epoxy-indole moiety. The configuration for the core skeleton of **3** was assigned to be as the same as that of **10** by a NOESY experiment and further confirmed by comparison of their NMR data. Based on the identical ECD curves (Fig. 3) of **3** and **1**, the stereochemistry of **3** was elucidated.

The molecular formula of compound **4** was determined as C_32_H_38_N_2_O_6_ according to its HRESIMS data (*m/z* 569.2609, [M + Na]^+^, cacld for C_32_H_38_N_2_O_6_Na, 569.2628). Exhaustive interpretation of the ^1^H and ^13^C NMR data (Tables 1 and 2) revealed that **4** was also a chaetoglobosin derivative. Moreover, its NMR data closely resembled those of **1** according to the consequent comparison, in which the major difference of the chemical shifts were noticed in the position of C-20 in the macrocyclic ring. A detailed comparison of the ^13^C NMR spectra of **4** and **1** suggested that the oxygenated methine (C-20, *δ*_C_ 72.6) in **1** was replaced by a carbonyl group (*δ*_C_ 205.9) in **4**. Furthermore, the carbon signal of the methine (C-6) at *δ*_C_ 74.0 in **1** shifted downfield to *δ*_C_ 77.7 in **4**, which could only be rationalized by supposing the change of configuration of C-6. This hypothesis was evidenced by NOESY correlation of Me-12/H-8 and the absence of the NOESY cross-peak between Me-12 and Me-11. Additional supporting evidences for the structure of **4** were provided by ^1^H–^1^H COSY, HMBC, NOESY, and ECD spectra, which allowed the confirmation of the planar structure and configuration of **4**.

Armochaetoglobin V (**5**) had the same molecular formula as that of **1**, suggesting that **5** is an isomer of **1**. Comparison of its NMR spectra (Tables 1 and 2) with that of **1** disclosed that the 6,7-diol in **1** was substituent by a 5,7-diol in **5**, which was evidenced by chemical shifts of C-5 and C-6 (*δ*_C_ 40.1 and 74.0 for **1**; *δ*_C_ 74.8 and 49.3 for **5**), and further confirmed by ^1^H–^1^H COSY cross-peak of H-6/H-7 and HMBC correlations from Me-12 to C-5, C-6, and C-7. The configurations of the two hydroxyls were assigned to be co-facial and *β*-oriented as revealed by NOESY correlations of Me-3 to Me-11 and Me-12, and of Me-12 to H-7. As the first member of chaetoglobosin with a hydroxyl at C-5, compound **5** was outstanding from the chaetoglobosin family.

Compound **6** was obtained as white amorphous powder, which possessed a molecular formula of C_32_H_38_N_2_O_5_, with 15 degrees of unsaturation, based on the [M + Na]^+^ peak at *m*/*z* 553.2660 (calcd for 553.26784) in the positive HRESIMS. The ^1^H and ^13^C NMR data of **6** (Tables 1 and 2) resembled those of **1**, which indicated that they share the same chaetoglobosin skeleton. However, the downfield shifted chemical shifts of H-7 (*δ*_H_ 5.43) and H-12 (*δ*_H_ 3.89) in compound **6**, suggested the *vicinal* diol at C-6 and C-7 in **1** were replaced by a Δ^6^ double bond in **6**. Moreover, the methyl (C-12) in **1** was oxygenated to be a hydroxymethyl (*δ*_C_ 61.4). These speculations were further supported by combination of HRESIMS and 2D NMR data (^1^H–^1^H COSY, HSQC, and HMBC). Further analyses of its NOESY spectrum and ECD curve established the absolute configuration of **6** and the trivial name armochaetoglobin W was given.

HRESIMS analysis of **7** indicated that it possessed the molecular formula of C_32_H_38_N_2_O_6_. The ^1^H and ^13^C NMR data for **7** (Tables 1 and 2) were similar to those of chaetoglobosin U (**11**)^20^. The major differences of them were the chemical shifts of C-6 and C-7 (*δ*_C_ 73.5 and 74.2 in **7**; *δ*_C_ 57.5 and 60.9 in **11**). The above analyses, combined with the HRESIMS, suggested that the epoxy ring of **11** was hydrolyzed to be a *vicinal* diol at C-6 and C-7 in compound **7**. The observed HMBC correlations from H-4 to C-6, from H-11 to C-4, C-5, and from C-6, and H-12 to C-5, C-6, and C-7 supported the above deduction. In addition, the similar NOESY spectra and identical ECD curves of the two compounds allowed the assignment of the absolute configuration of **7**. Therefore, the structure of compound **7** was established as shown and named armochaetoglobin X.

The HRESIMS data of **8** exhibited an ion peak at *m*/*z* 535.2509 (calcd for C_32_H_36_N_2_O_4_Na, 535.2573), suggesting a molecular formula of C_32_H_36_N_2_O_4_, with 16 degrees of unsaturation. Its NMR spectra (Tables 1 and 2) were in general similar to those of **11**, especially in rings A–C. However, inspection of the ^1^H NMR spectrum of **8** revealed a new olefinic proton at *δ*_H_ 5.91 that was not observed for **11**. In addition, the associated carbon signal of C-18 was downfield shifted from 147.2 to 183.5 ppm. Examination of the 2D NMR data of **8** confirmed that an additional olefinic proton was assigned to C-18 of the trisubstituted double bond in **8**, which replaced the hydroxyl at the tetrasubstituted double bond in **11**. The relative configuration of **8** was established by a NOESY experiment, in which the correlation pattern of the rings A and B were similar to other chaetoglobosin. However, the observed NOESY correlations of H-21 to 16-Me and H-13 suggested that their co-facial and *α*-oriented. In addition, the *β* conformation of H-17 was determined by NOESY correlation of H-17 and H-16, and aided by the lack of NOESY cross-peak between H-17 and 16-Me.

**Figure 4:**
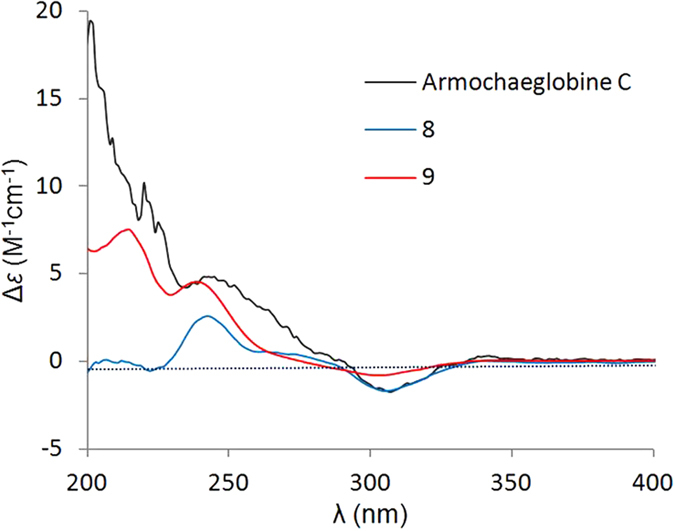
ECD curves of armochaeglobine C and compounds 8 and 9.

The absolute configuration of **8** was assigned comparison of its ECD spectrum (Fig. 4) with that of armochaeglobine C^25^, whose absolute configuration was determined by X-ray diffraction analysis and reported by us previously.

Armochaetoglobin Z (**9**) was obtained as a colorless amorphous powder. Its molecular formula was determined as C_32_H_36_N_2_O_4_ by HRESIMS. Detailed comparison of the NMR data of **9** with that of **8** suggested a similar structure for them. The main differences of the two compounds were that the epoxy group in **8** was substituted by a double bond at C-5 and a hydroxyl at C-7 in **9**, which was evidenced by analyses of their NMR data and HMBC correlations from Me-11 to C-4, C-5, and C-6, from Me-12 to C-5, C-6, C-7, and from H-7 to H-8 and H-13. The 7-OH was revealed to *β*-oriented according to NOESY correlations of H-7 to Me-11 and Me-12. In addition, the ECD curve of **9** (Fig. 4) was nearly identical to that of armochaeglobine C and **8**, leading to the final assignment of its absolute configuration.

### Evaluation of cytotoxicity in human cell lines

Cytochalasans are commonly reported to exhibit cytotoxic effects and some of them have been considered as starting molecular for anti-tumor drug candidates. Therefore, in this case, compounds **1**–**9** were all evaluated for their cytotoxic activities against five human tumor cell lines (HL-60, A-549, SMMC-7721, MCF-7, and SW-480) and the immortalized non-cancerous Beas-2B human bronchial epithelial cell line, *in vitro*, by the MTS method (Table S1, SI). According to the reported structure-activity relationships, the epoxide at C-6/C-7 and the aromatic substituent at C-10 were required for cytotoxicity^1^, and our results were consistent with literature. Compound **8** exhibited most potent cytotoxic activities toward HL-60, A-549, SMMC-7721, and SW-480 cell lines, and in contrast, compound **9**, with the epoxide hydrolysed, only exhibited cytotoxicity against HL-60 and A-549 cell lines. Moreover, compound **3** was inactive at a concentration of 40 *μ*M, which might be due to the destruction of the aromatic feature of the substituent at C-10 induced by epoxidation at the indole ring.

In summary, the fungus *Chaetomium globosum* has provided a rich spectrum of indole-based cytochalasans. In this study, nine new highly oxygenated cytochalasan derivatives (**1**–**9**), along with eight known chaetoglobosins, were isolated from the rice culture of *Chaetomium globosum*, isolated from the common pillbug (*Armadillidium vulgare*). The structures of the new compounds were determined by a combination of spectroscopic and ECD computational methods. A literature search revealed that changes among chaetoglobosins commonly occur in the positions of C-5–C-7, C-12, and C-19–C-22, with the other positions relatively fixed. Compound **2** is the first member of the chaetoglobosin family with an acetyl group. Compound **3** and **5** present to be the first examples of natural chaetoglobosins characterized by oxygenation of indole moiety and hydroxylation of C-5, respectively. In addition, compounds **8** and **9** exhibited significant cytotoxic activities with IC_50_ values ranging from 10.45 to 30.42 *μ*M.

## Methods

### General experimental procedures

Optical rotations were determined with a Perkin-Elmer 341 polarimeter. UV, CD, and FT-IR spectra were measured using a Varian Cary 50, a JASCO-810 CD spectrometer, and a Bruker Vertex 70 instruments, respectively. NMR spectra were recorded on a Bruker AM-400 spectrometer, and the ^1^H and ^13^C NMR chemical shifts were referenced to the solvent or solvent impurity peaks for CD_3_OD (*δ*_H_ 3.31 and *δ*_C_ 49.0) and DMSO-*d*_6_ (*δ*_H_ 2.50 and *δ*_C_ 39.5). High-resolution electrospray ionization mass spectra (HRESIMS) were carried out in the positive ion mode on a Thermo Fisher LC-LTQ-Orbitrap XL spectrometer. Semipreparative HPLC was carried out on an Agilent 1200 quaternary system with a UV detector or a Dionex HPLC system equipped with an Ultimate 3000 pump, an Ultimate 3000 autosamper injector, and an Ultimate 3000 DAD detector controlled by Chromeleon software (version 6.80), using a reversed-phased C_18_ column (5 *μ*m, 10 × 250 mm). Column chromatography was performed using silica gel (100–200 and 200–300 mesh), ODS (50 *μ*m), and Sephadex LH-20. Thin-layer chromatography (TLC) was performed with silica gel 60 F_254_ and RP-C_18_ F_254_ plates.

### Fungal material

The strain *Chaetomium globosum* TW1-1 was isolated from *Armadillidium vulgare* in November 2012 at Tongji Medical College, Hubei Province, China. The 16S RNA sequence data for this strain have been submitted to the DDBJ/EMBL/GenBank under accession No. KF993614. A voucher sample, CCM20121113, was deposited in the culture collection of Tongji Medical College, Huazhong University of Science and Technology.

### Fermentation, extraction, and isolation

The strain was cultured on potato dextrose agar (PDA) at 28 °C for 7 days in stationary phase to prepare the seed culture. Then the Agar plugs were cut into small pieces and inoculated into 250 Erlenmeyer flasks (1 L), previously sterilized by autoclaving, each containing 200 g rice and 200 mL distilled water. All flasks were incubated at 28 °C for 28 days. Following incubation, the growth of cells was stopped by adding 300 mL EtOAc to each flask, and the culture was homogenized. Then the suspension was extracted by ultrasonic extraction with methanol for five times at room temperature. The methanol was removed under reduced pressure to yield a brown extract (827 g), and then suspended in H_2_O (5L), and partitioned with EtOAc against water. The EtOAc soluble extract (230 g) was separated by chromatography on a silica gel column (80–120 mesh, 10 × 120 cm, CC) eluting with CH_2_Cl_2_/MeOH (50:1–0:1) to yield six fractions (Fr. 1–Fr. 8). Fr.3 was further purified by MPLC ODS (40%–100% MeOH in H_2_O) to obtain four subfractions (Fr. 4.1−Fr. 4.4). Fr.4.2 was purified over Sephadex LH-20 (MeOH) followed by MPLC ODS (40% MeOH in H_2_O) and finally by repeated semi-preparative HPLC to yield **8** (11.6 mg), **10** (6.7 mg), **11** (20.4 mg), **13** (17.6 mg), and **14** (6.3 mg). Fr.4.3 was separated over repeated silica gel CC (petroleum ether to acetone, 5:1 → 2:1), Sephadex LH-20 (MeOH), and semi-preparative HPLC to yield **9** (7.2 mg), **12** (3.6 mg), **15** (6.4 mg), **16** (12.9 mg), and **17** (4.5 mg). Fr. 4 was applied to MPLC ODS (40%–100% MeOH in H_2_O) and then to Sephadex LH-20 (MeOH) and repeated silica gel CC (petroleum ether to acetone, 4:1 → 2:1) to obtain two mixtures (I and II). Mixture I was purified by repeated semi-preparative HPLC (50% MeOH in H_2_O and 40% MeCN in H_2_O) to obtain **1** (3.0 mg) and **2** (27.3 mg), and mixture II was purified by semi-preparative HPLC (55% MeOH in H_2_O) to obtain **3** (4.8 mg). Fr. 6 was applied to MPLC ODS (20%–80% MeOH in H_2_O) to obtain six subfractions (Fr. 6.1−Fr. 6.6). Fr. 6.2 was separated with Sephadex LH-20 (MeOH) and silica gel CC (petroleum ether to acetone, 2:1) to obtain two mixtures (Fr. 6.2.1–Fr. 6.2.2). Fr. 6.2.1 was purified by semi-preparative HPLC (45% MeOH in H_2_O) to obtain **4** (4.5 mg) and **6** (2.0 mg), while Fr 6.2.2 was purified by semi-preparative HPLC (36% MeCN in H_2_O) to yield **5** (2.1 mg) and **7** (2.0 mg).

*Armochaetoglobin S* (**1**): colorless powder; 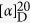
–13.5 (*c* = 0.53, MeOH); UV (MeOH) *λ*_max_ (log *ε*) = 203 (4.36) and 222 (4.45) nm; IR *v*_max_ = 3379, 2966, 2929, 1682, 1554, 1431, and 1268 cm^–1^; CD (MeOH) *λ*_max_ (Δ*ε*) 224 (−7.9), 272 (+0.6) nm; For ^1^H NMR (400 MHz) and ^13^C NMR (100 MHz) data, see Tables 1 and 2; HRESIMS [M + H]^+^ *m/z* 549.2936 (calcd for C_32_H_42_N_2_O_6_, 549.2965).

*7-O-acetylarmochaetoglobin S* (**2**): colorless powder; 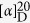
+ 12.7 (*c* = 0.34, MeOH); UV (MeOH) *λ*_max_ (log *ε*) = 203 (4.42) and 221 (4.47) nm; IR (KBr) *v*_max_ = 3389, 2968, 2930, 1686, 1551, 1454, and 1239 cm^–1^; CD (MeOH) *λ*_max_ (Δ*ε*) 223 (−10.3), 271 (0.6) nm; For ^1^H NMR (400 MHz) and ^13^C NMR (100 MHz) data, see Tables 1 and 2; HRESIMS [M + Na]^+^ *m/z* 613.2859 (calcd for C_34_H_42_N_2_O_7_Na, 613.2890).

*Armochaetoglobin T* (**3**): colorless powder; 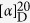
+ 21.0 (*c* = 0.23, MeOH); UV (MeOH) *λ*_max_ (log *ε*) = 204 (4.55) and 222 (4.59) nm; IR *v*_max_ = 3388, 2973, 1670, 1616, 1562, 1409, 1333, and 1192 cm^–1^; CD (MeOH) *λ*_max_ (Δ*ε*) 227 (−6.4) nm; For ^1^H NMR (400 MHz) and ^13^C NMR (100 MHz) data, see Tables 1 and 2; HRESIMS [M + Na]^+^ *m/z* 569.2628 (calcd for C_32_H_38_N_2_O_6_Na, 569.2606).

*Armochaetoglobin U* (**4**): colorless powder; 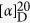 –61.0 (*c* = 0.20, MeOH); UV (MeOH) *λ*_max_ (log *ε*) = 221 (4.48) nm; IR (KBr) *v*_max_ = 3378, 2969, 2927, 1703, 1454, 1430, 1361, and 1228 cm^–1^; CD (MeOH) *λ*_max_ (Δ*ε*) 223 (−8.6), 291 (−5.6) nm; For ^1^H NMR (400 MHz) and ^13^C NMR (100 MHz) data, see Tables 1 and 2; HRESIMS [M + Na]^+^ *m/z* 569.2609 (calcd for C_32_H_38_N_2_O_6_Na, 569.2628).

*Armochaetoglobin V* (**5**): colorless powder; 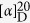 –35.6 (*c* = 0.06, MeOH); UV (MeOH) *λ*_max_ (log *ε*) = 222 (4.56) and 269 (3.68), nm; IR (KBr) *v*_max_ = 3413, 2966, 2928, 1685, 1453, 1435, and 1234 cm^–1^; CD (MeOH) *λ*_max_ (Δ*ε*) 222 (−6.3) nm; For ^1^H NMR (400 MHz) and ^13^C NMR (100 MHz) data, see Tables 1 and 2; HRESIMS [M + Na]^+^ *m/z* 571.2767 (calcd for C_32_H_40_N_2_O_6_Na, 571.2784).

*Armochaetoglobin W* (**6**): colorless powder; 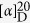 + 6.4 (*c* = 0.34, MeOH); UV (MeOH) *λ*_max_ (log *ε*) = 203 (4.55), 222 (4.62), and 283 (3.79) nm; IR (KBr) *v*_max_ = 3379, 2964, 2926, 1700, 1454, 1431, 1382, and 1048 cm^–1^; CD (MeOH) *λ*_max_ (Δ*ε*) 227(−9.8) nm; For ^1^H NMR (400 MHz) and ^13^C NMR (100 MHz) data, see Tables 1 and 2; HRESIMS [M + Na]^+^ *m/z* 553.2660 (calcd for C_32_H_38_N_2_O_5_Na, 553.2678).

*Armochaetoglobin X* (**7**): colorless powder; 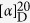 –70.6 (*c* = 0.07, MeOH); UV (MeOH) *λ*_max_ (log *ε*) = 221 (4.43) and 269 (3.93), nm; IR (KBr) *v*_max_ = 3318, 2967, 2927, 1700, 1428, 1360, 1228, and 1108 cm^–1^; CD (MeOH) *λ*_max_ (Δ*ε*) 221 (−10.5), 257 (−9.6) nm; For ^1^H NMR (400 MHz) and ^13^C NMR (100 MHz) data, see Tables 1 and 2; HRESIMS [M + H]^+^ *m/z* 547.2789 (calcd for C_32_H_39_N_2_O_6_, 547.2808).

*Armochaetoglobin Y* (**8**): colorless powder; 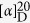 + 75.2 (*c* = 0.46, MeOH); UV (MeOH) *λ*_max_ (log *ε*) = 203 (4.50), 222 (4.62), 283 (3.80) nm; IR (KBr) *v*_max_ = 3401, 2966, 2924, 1688, 1616, 1454, 1432, 1382, and 1106 cm^–1^; CD (MeOH) *λ*_max_ (Δ*ε*) 206 (+0.1), 222 (−0.6), 243 (+2.6) nm; For ^1^H NMR (400 MHz) and ^13^C NMR (100 MHz) data, see Tables 1 and 2; HRESIMS [M + Na]^+^ *m/z* 535.2509 (calcd for C_32_H_36_N_2_O_4_Na, 535.2573).

*Armochaetoglobin Z* (**9**): colorless powder; 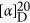 + 73.6 (*c* = 0.39, MeOH); UV (MeOH) *λ*_max_ (log *ε*) = 203 (4.32) and 222 (4.26) nm; IR (KBr) *v*_max_ = 3380, 2952, 2914, 1685, 1614, 1434, 1379, 1227, and 1106 cm^–1^; CD (MeOH) *λ*_max_ (Δ*ε*) 215 (+7.5), 239 + 4.5) nm; For ^1^H NMR (400 MHz) and ^13^C NMR (100 MHz) data, see Tables 1 and 2; HRESIMS [M + H]^+^ *m/z* 513.2734 (calcd for C_32_H_37_N_2_O_4_, 513.2753).

### Cytotoxic assay

Five human cancer cell lines including HL-60, SMMC-7721, A-549, MCF-7, and SW-480, as well as the noncancerous cell line, the Beas-2B human bronchial epithelial cell line, were used in the cytotoxic activity assay. Cytotoxic activity was measured as described in our previous report^32^.

## Additional Information

**How to cite this article**: Chen, C. *et al.* Nine new cytochalasan alkaloids from Chaetomium globosum TW1-1 (Ascomycota, Sordariales). *Sci. Rep.* **6**, 18711; doi: 10.1038/srep18711 (2016).

## Supplementary Material

Supplementary Information

## Figures and Tables

**Figure 1:**
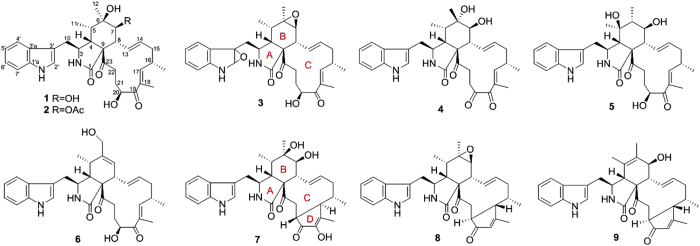
Structures of compounds 1–9.

**Table 1:** ^1^H-NMR data of compounds **1–9** (400MHz, *J* in Hz).

Position	1^a^	2^a^	3^b^	4^a^	5^a^	6^b^	7^a^	8^a^	9^a^
3	3.81 dd (9.0, 4.6)	3.78 dd (8.9, 5.2)	4.13 dt (11.0, 5.4)	4.41 dd (8.9, 3.7)	3.83 dd (8.0, 6.4)	3.32 m	3.79 m	3.80 m	3.53 dd (9.8, 5.5)
4	2.50 m	2.75 m	2.52 m	2.10 m	2.63 br s	2.48 m	2.70 m	2.75 br d (6.4)	3.18 m
5	2.02 m	1.98 m	1.56 m	1.73 m		2.24 m	2.15 m	1.68 m	
6					1.58 dd (8.9, 6.9)				
7	3.16 d (11.7)	4.74 d (11.7)	2.89 d (4.9)	3.53 d (12.5)	3.07 dd (10.9, 8.9)	5.43 br s	3.31 overlapped	2.90 d (5.9)	4.03 br d (9.7)
8	2.63 dd (11.7, 10.3)	2.85 m	2.49 m	2.67 m	2.82 m	3.73 m	2.71 m	2.32 dd (10.5, 6.0)	2.29 dd (10.9, 9.7)
10	2.98 dd (14.6, 4.8)	2.91 dd (14.7, 5.7)	2.31 dd (12.4, 5.4)	3.13 dd (14.9, 3.4)	2.94 m	2.77 dd (14.4, 5.1)	2.87 dd (14.6, 5.2)	2.85 dd (14.4, 4.4)	2.88 dd (14.0, 5.5)
	2.80 dd (14.6, 4.7)	2.83 dd (14.7, 5.1)	1.44 dd (12.4, 11.0)	2.85 dd (14.9, 3.3)	2.84 m	2.68 dd (14.4, 4.9)	2.73 dd (14.6, 4.0)	2.59 dd (14.4, 7.7)	2.45 dd (14.0, 9.8)
11	1.02 d (6.6)	1.02 d (7.3)	1.07 d (7.3)	1.21 d (7.1)	1.01 s	0.96 d (7.2)	1.00 d (7.2)	0.79 d (7.3)	1.16 s
12	1.22 s	1.15 s	1.30 s	1.15 s	1.08 d (7.4)	3.89 br s	1.18 s	1.20 s	1.61 s
13	5.94 dd (14.5, 10.8)	5.98 dd (14.1, 10.2)	6.03 dd (14.6, 10.7)	5.58 dd (14.5, 10.6)	6.35 dd (15.0, 10.5)	6.25 dd (15.6, 10.5)	5.93 dd (15.3, 9.9)	6.37 dd (15.4, 1.5)	6.37 dd (14.3, 10.9)
14	2.12 ddd (14.5, 10.9, 2.5)	5.16 ddd (14.1, 11.0, 2.5)	5.25 ddd (14.6, 10.1, 2.6)	4.94 ddd (14.5, 11.0, 2.1)	5.20 ddd (15.0, 10.9, 2.4)	5.06 ddd (15.6, 10.9, 2.6)	5.11 ddd (15.3, 11.6, 3.8)	5.27 ddd (15.4, 11.4, 2.4)	5.33 ddd (14.3, 12.1. 2.3)
15	2.40 m	2.34 m	2.44 m	2.35 m	2.49 br d (14.8)	2.31 m	2.24 br d (12.5)	2.62 dd (14.0, 5.4)	2.68 m
	1.97 m	1.98 m	1.96 m	1.84 m	2.12 dt (14.8, 11.0)	1.92 m	1.85 dt (12.5, 11.7)	1.83 m	1.91 m
16	2.74 m	2.75 m	2.77 m	2.72 m	2.82 m	2.66 m	1.44 m	2.38 m	2.47 m
17	6.19 d (9.1)	6.21 d (8.7)	6.29 d (8.7)	5.98 d (10.2)	6.31 d (9.2)	6.15 d (9.2)	2.19 d (6.6)	2.87 br s	3.15 m
19								5.91 s	6.02 s
20	4.66 dd (6.2, 4.7)	4.71 t (5.5)	4.79 t (5.8)		4.80 dd (11.7, 5.4)	4.48 dd (11.2, 6.7)			
21	1.60 m	1.68 m	1.57 m	2.24 m	1.83 m	1.47 m	2.02 m	2.53 dd (7.3, 5.6)	2.56 m
	1.36 m	1.46 m	1.42 m	1.62 m	1.70 m	1.27 m			
22	2.50 m	2.64 m	2.63 m	2.49 m	3.05 m	2.52 m	2.70 m	3.09 dd (14.8, 5.6)	3.69 dd (16.3, 6.2)
	1.82 m	2.12 m	2.50 m	0.89 m	2.88 m	1.75 m	0.84 m	1.86 dd (14.8, 7.3)	2.65 dd (16.3, 4.9)
2′	7.03 br s	7.06 br s	4.99 d (3.5)	7.11 s	7.10 br s	7.09 d (1.9)	6.95 s	6.96 s	7.00 br s
4′	7.51 d (7.8)	7.52 d (7.8)	7.13 d (7.2)	7.58 d (7.7)	7.51 d (7.7)	7.49 d (7.8)	7.48 d (7.7)	7.48 d (7.8)	7.51 d (7.8)
5′	7.02 t (7.4)	7.02 t (7.4)	6.64 t (7.4)	7.06 t (7.9)	7.02 t (7.6)	6.96 t (7.0)	7.01 t (7.2)	7.01 dd (7.8, 7.2)	7.01 t (7.3)
6′	7.09 t (7.2)	7.09 t (7.2)	7.04 t (7.6)	7.12 t (7.3)	7.07 t (7.7)	7.03 t (7.1)	6.97 t (7.6)	7.06 dd (7.9, 7.2)	7.09 t (7.5)
7′	7.33 d (8.0)	7.33 d (8.1)	6.55 d (7.9)	7.35 d (8.0)	7.33 d (7.1)	7.30 d (8.0)	7.26 d (8.0)	7.28 d (7.9)	7.33 d (8.1)
16-Me	1.00 d (6.5)	1.01 d (6.7)	1.00 d (6.7)	0.98 d (6.6)	1.06 d (7.3)	0.97 d (6.6)	0.97 d (7.1)	0.67 d (6.8)	0.78 d (6.8)
18-Me	1.76 s	1.77 s	1.71 s	1.78 s	1.83 br s	1.68 s	2.66 s	2.09 s	2.16 s
C-2′′		1.98s							

^a^in CD_3_OD.

^b^in DMSO–*d*_6._

**Table 2:** ^13^C NMR for compounds 1–9 (100 MHz, *J* in Hz).

No	1^a^	2^a^	3^b^	4^a^	5^a^	6^b^	7^a^	8^a^	9^a^
1	176.8	176.5	170.2	176.6	177.4	173.6	176.0	176.8	176.8
3	54.4	55.2	59.8	54.8	55.4	53.5	53.9	54.0	59.3
4	45.8	44.8	46.9	46.8	55.2	49.4	46.3	49.4	50.2
5	40.1	41.3	34.6	39.1	74.8	33.7	40.9	38.0	128.3
6	74.0	73.8	57.6	77.7	49.3	142.6	73.5	58.9	134.4
7	73.6	74.9	61.1	76.4	73.8	125.6	74.2	62.3	70.1
8	47.2	46.0	45.2	47.6	45.0	45.3	50.9	51.5	55.2
9	65.6	65.1	68.5	65.3	65.4	66.6	67.9	68.0	64.7
10	33.4	32.9	47.3	31.8	33.0	32.4	33.1	34.2	33.0
11	13.5	13.3	13.8	13.3	20.1	11.9	12.6	12.7	17.4
12	24.8	24.8	20.1	22.2	13.8	61.4	24.9	19.5	14.5
13	129.4	128.5	129.5	128.1	129.9	130.7	130.6	131.5	132.6
14	134.9	135.4	132.5	135.1	135.7	130.7	134.3	133.5	134.0
15	41.9	41.8	40.3	40.8	42.1	40.2	45.3	39.5	39.6
16	34.5	34.5	32.9	34.5	34.5	32.8	43.8	32.5	32.8
17	149.9	149.9	147.3	156.0	150.4	147.2	54.2	49.8	49.6
18	136.3	136.2	134.8	132.6	136.0	134.6	149.7	183.5	183.7
19	205.7	205.5	204.1	197.5	205.3	203.8	148.6	129.5	129.8
20	72.4	72.6	72.0	205.9	73.0	70.9	204.4	212.0	211.6
21	31.8	32.1	31.0	33.4	32.4	30.1	52.8	44.6	44.4
22	38.5	38.8	37.4	37.2	39.5	37.6	42.8	42.7	41.9
23	209.9	209.9	207.2	208.5	211.4	209.2	213.9	211.2	210.6
2′	125.6	125.3	82.4	126.5	124.5	124.5	125.8	125.1	124.7
3′	109.9	110.3	91.9	109.6	110.0	109.0	109.5	110.4	111.5
3′a	129.2	129.0	131.0	129.7	128.8	127.7	129.2	128.8	128.5
4′	119.3	119.2	122.6	119.6	119.2	118.3	119.4	119.3	119.2
5′	120.1	120.1	117.6	120.5	119.8	118.3	120.1	120.1	119.9
6′	122.5	122.5	129.0	122.7	122.4	120.7	122.3	122.5	122.4
7′	112.5	112.5	109.6	112.4	112.4	111.3	112.6	112.3	112.4
1′a	137.9	138.0	148.4	137.8	138.1	136.0	138.0	138.1	138.1
16-Me	20.1	20.1	19.8	19.8	20.1	19.7	21.7	16.3	15.9
18-Me	12.4	12.3	12.1	10.9	12.2	12.0	16.9	17.7	17.7
1′′		172.3							
2′′		20.8							

^a^in CD_3_OD.

^b^in DMSO–*d*_6._
