# Supplementary material for: Nine new cytochalasan alkaloids from Chaetomium globosum TW1-1 (Ascomycota, Sordariales)
Source: Sci Rep. 2016 Jan 7;6:18711. doi: 10.1038/srep18711 (PMC4703973; doi:10.1038/srep18711)

Nine new cytochalasan alkaloids from Chaetomium globosum TW1-1 (Ascomycota, Sordariales)

Chunmei Chen[1], Qingyi Tong[1], Hucheng Zhu[1], Dongdong Tan[1], Jinwen Zhang[3], Yongbo Xue[1], Guangmin Yao[1], Zengwei Luo[1], Jianping Wang[1,*], Yanyan Wang[2,*] & Yonghui Zhang[1,*]

[1] Hubei Key Laboratory of Natural Medicinal Chemistry and Resource Evaluation, School of Pharmacy, Tongji Medical College, Huazhong University of Science and Technology, Wuhan 430030, China

[2] First College of Clinical Medical Science，China Three Gorges University & Yichang Central People’s Hospital, Yichang 443003, China

[3] Tongji Hospital Affliated to Tongji Medical College, Huazhong University of Science and Technology, Wuhan 430030, China

Content

Cytotoxic activities of compounds **1–9** 1

UV, IR, MS, and NMR spectrum of compound **1** 2

UV, IR, MS, and NMR spectrum of compound **2** 5

UV, IR, MS, and NMR spectrum of compound **3** 10

UV, IR, MS, and NMR spectrum of compound **4** 14

UV, IR, MS, and NMR spectrum of compound **5** 19

UV, IR, MS, and NMR spectrum of compound **6** 23

UV, IR, MS, and NMR spectrum of compound **7** 28

UV, IR, MS, and NMR spectrum of compound **8** 32

UV, IR, MS, and NMR spectrum of compound **9** 37

Table S1. Cytotoxic activities of compounds 1–9 (μM)

Compound	HL-60	SMMC-7721	A-549	MCF-7	SW480	BEAS-2B
**1**	>40	>40	>40	>40	>40	>40
**2**	>40	>40	>40	>40	>40	>40
**3**	>40	>40	>40	>40	>40	>40
**4**	>40	>40	>40	>40	>40	>40
**5**	>40	>40	>40	>40	>40	>40
**6**	>40	>40	>40	>40	>40	>40
**7**	>40	>40	>40	>40	>40	>40
**8**	23.12	14.13	17.08	>40	30.42	23.87
**9**	10.45	>40	23.11	>40	>40	10.45
DDP	1.93	10.21	6.59	8.20	12.16	9.07

UV spectrum of compound **1**

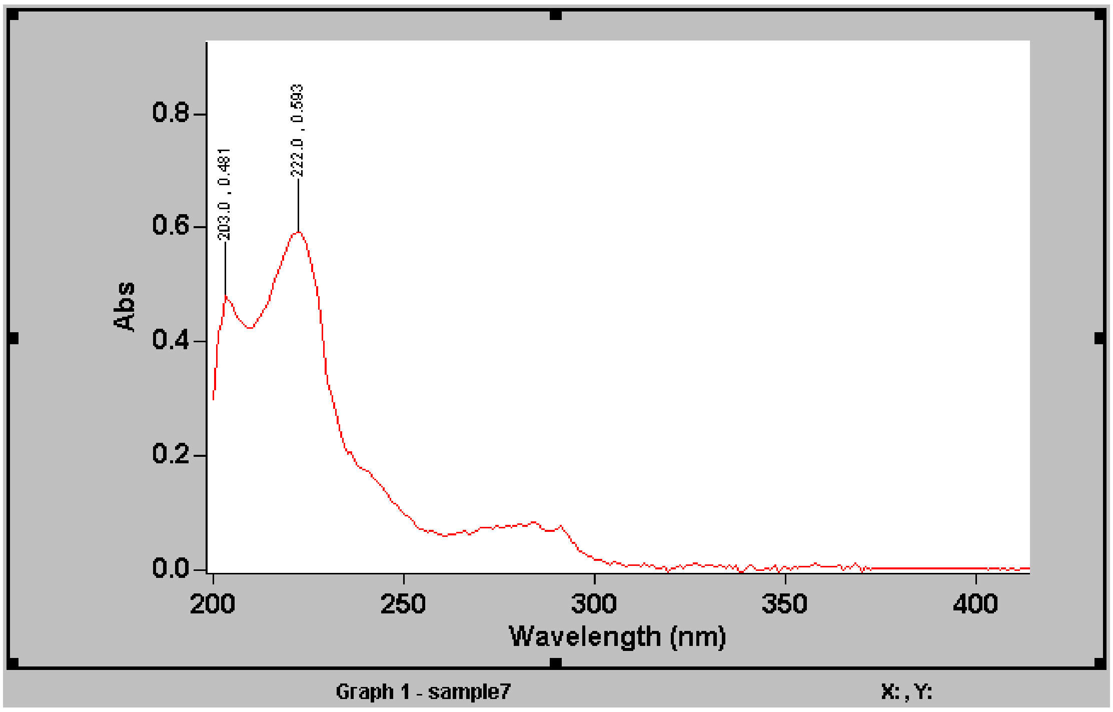

IR spectrum of compound **1**

E:\同济医学院\张勇慧\20140325\CG2-7-11.0

仪器型号：Bruker Vertex 70

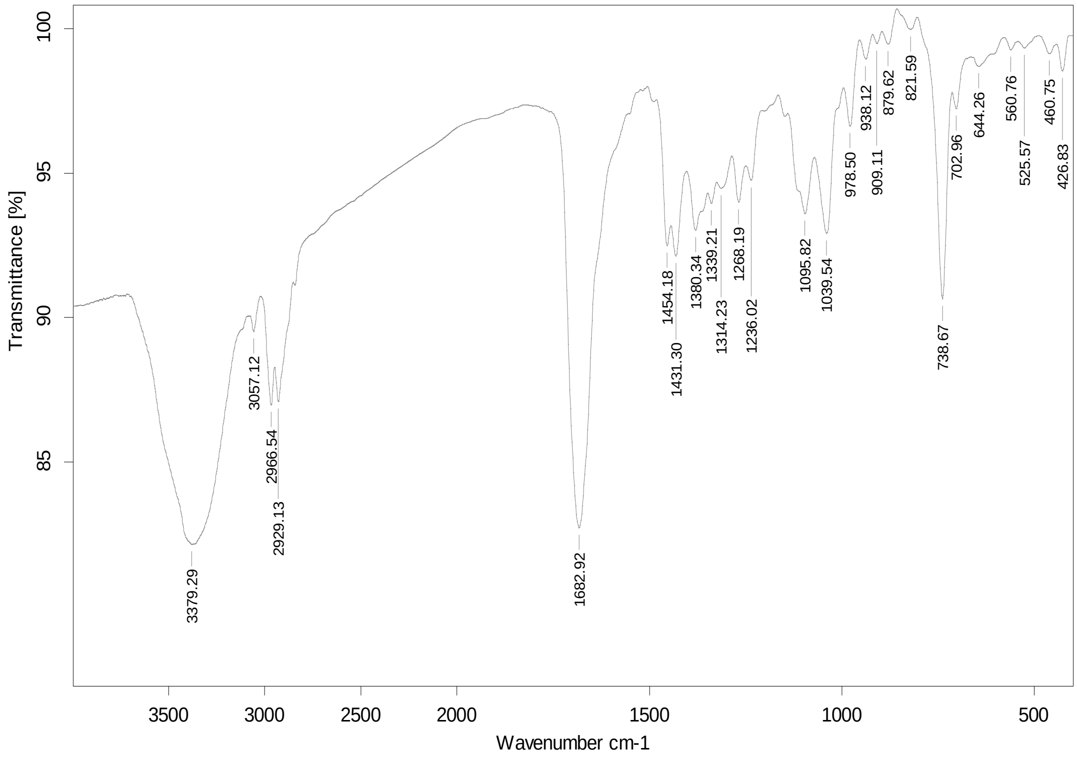

HRESIMS spectrum of compound **1**

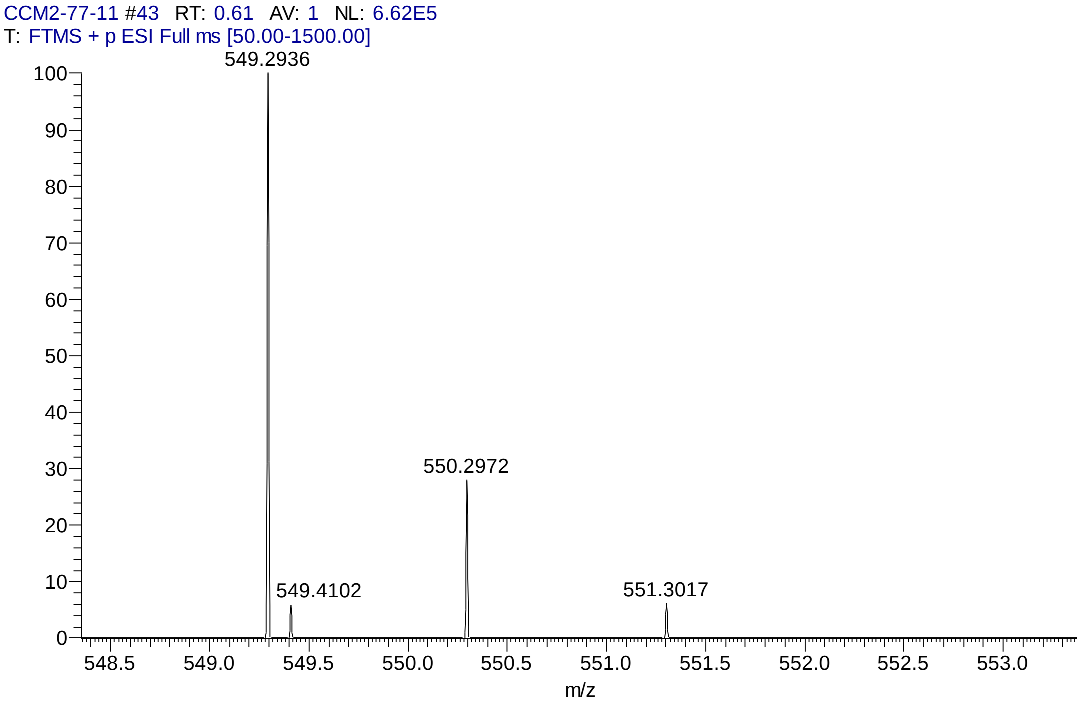

^{1}H NMR spectrum of compound **1**

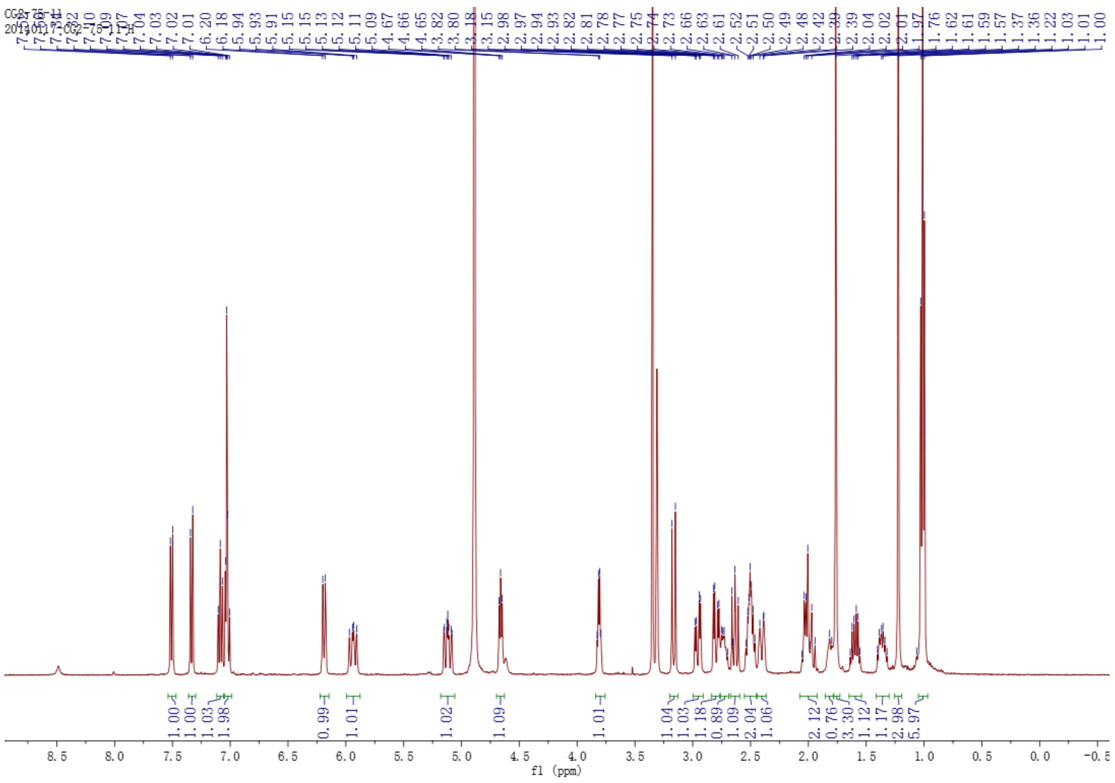

^{13}C NMR and DEPT spectra of compound **1**

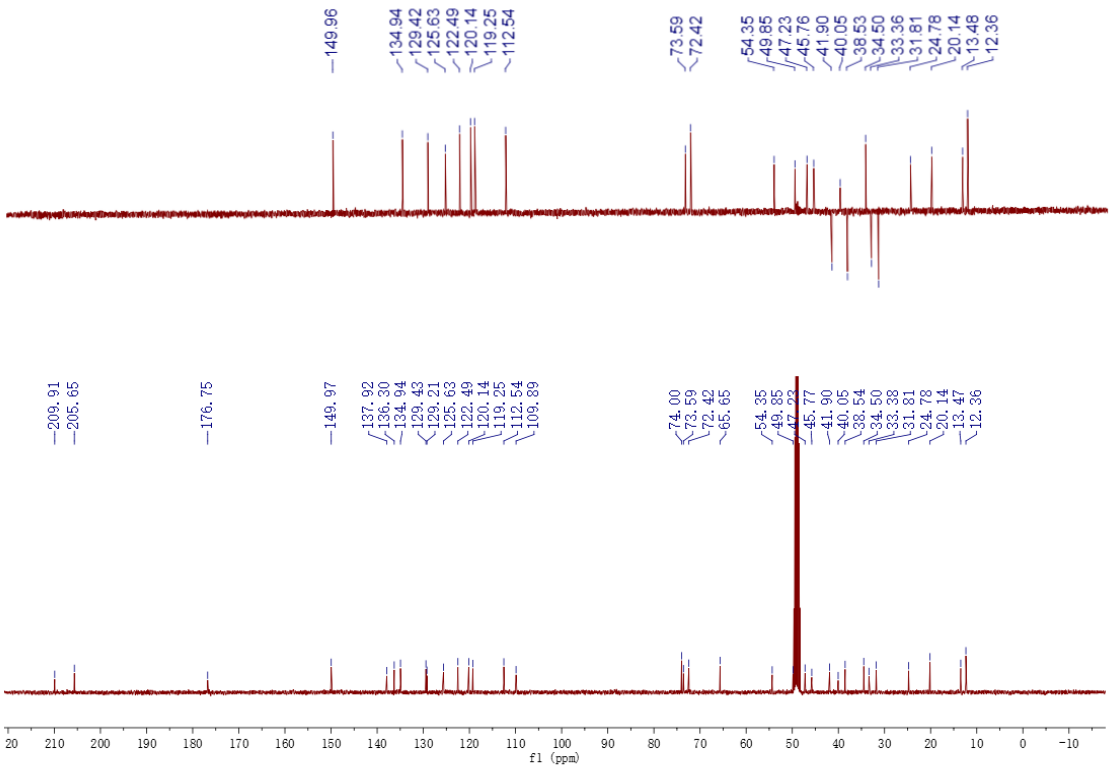

HSQC spectrum of compound **1**

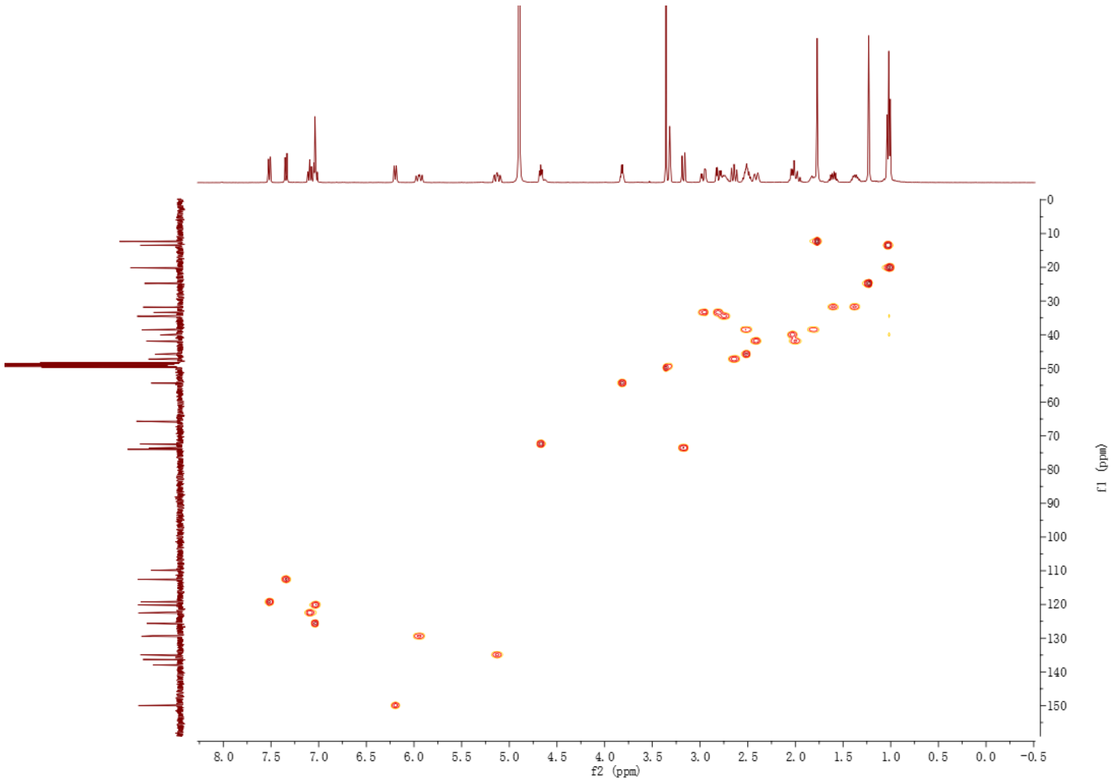

HMBC spectrum of compound **1**

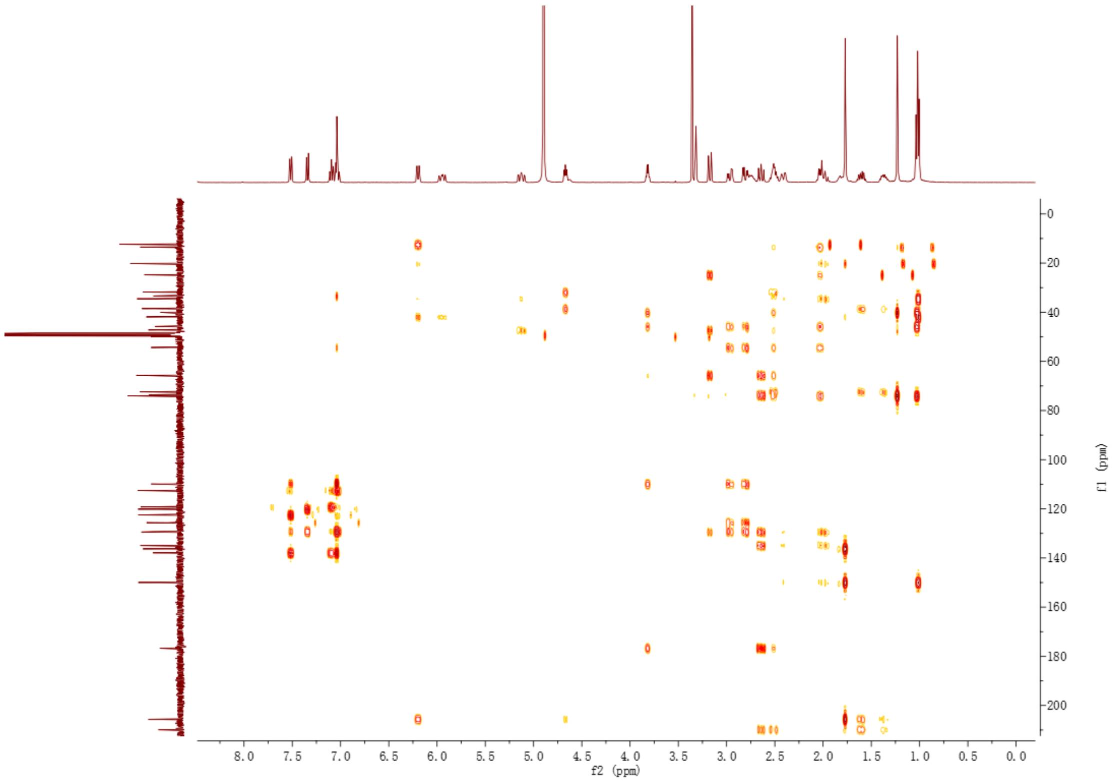

^{1}H–^{1}H COSY spectrum of compound **1**

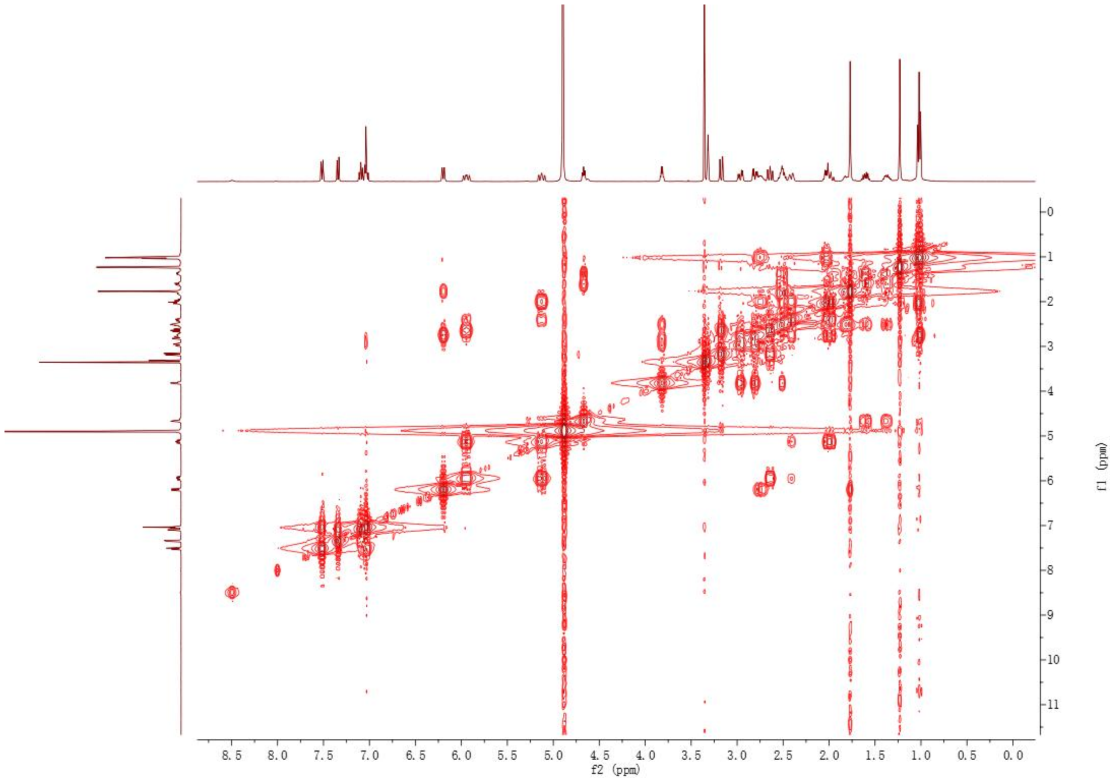

NOESY spectrum of compound **1**

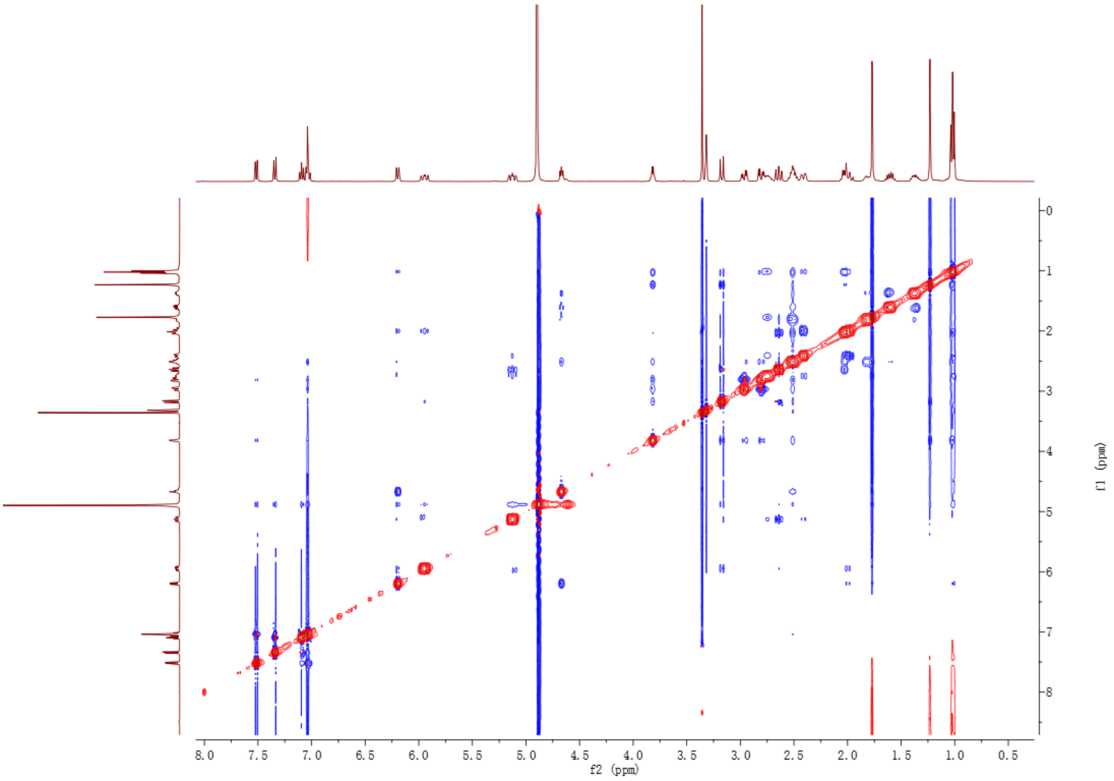

UV spectrum of compound **2**

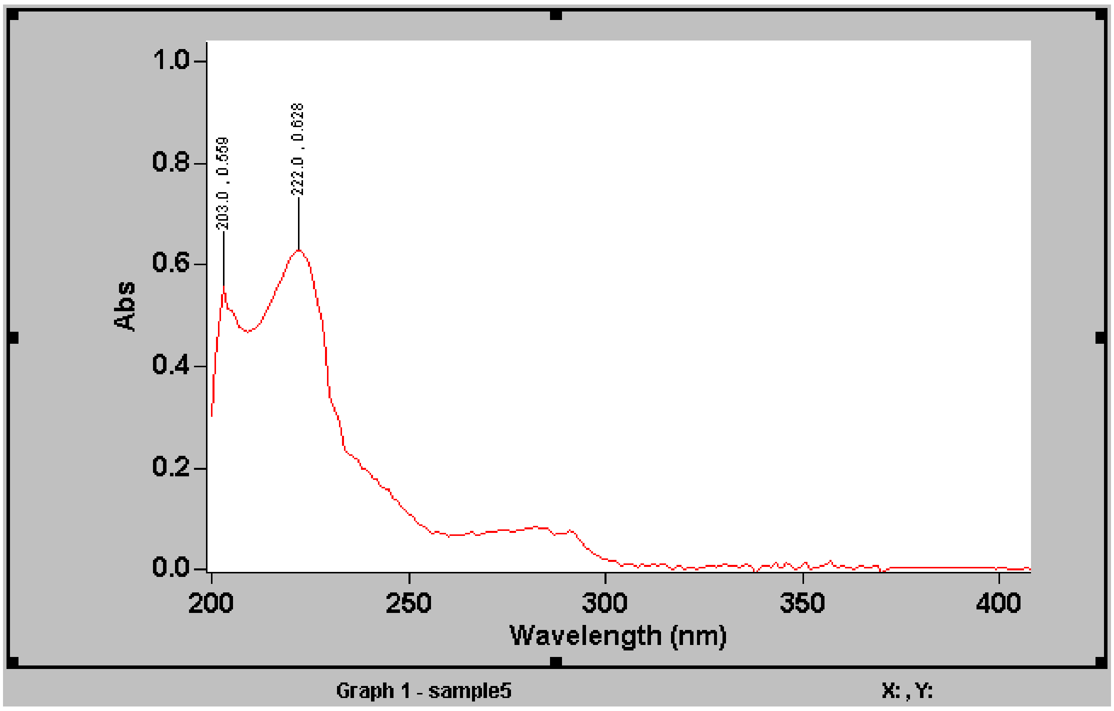

IR spectrum of compound **2**

E:\同济医学院\张勇慧\20140325\CG2-79-2.0

仪器型号：Bruker Vertex 70

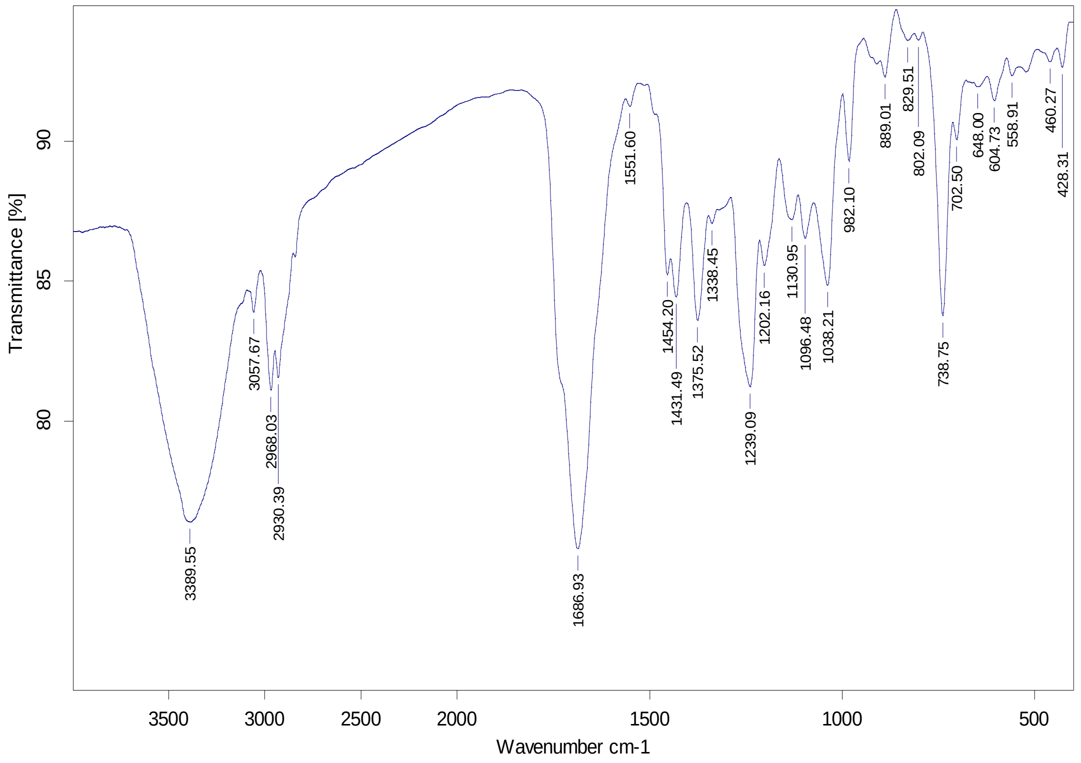

HRESIMS spectrum of compound **2**

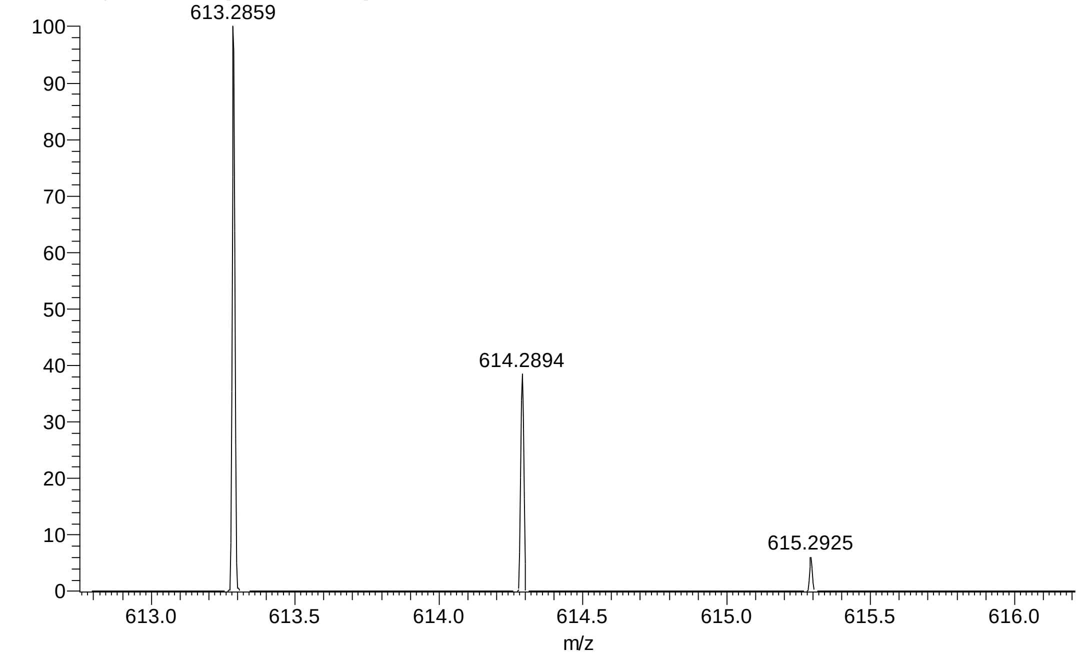

^{1}H NMR spectrum of compound **2**

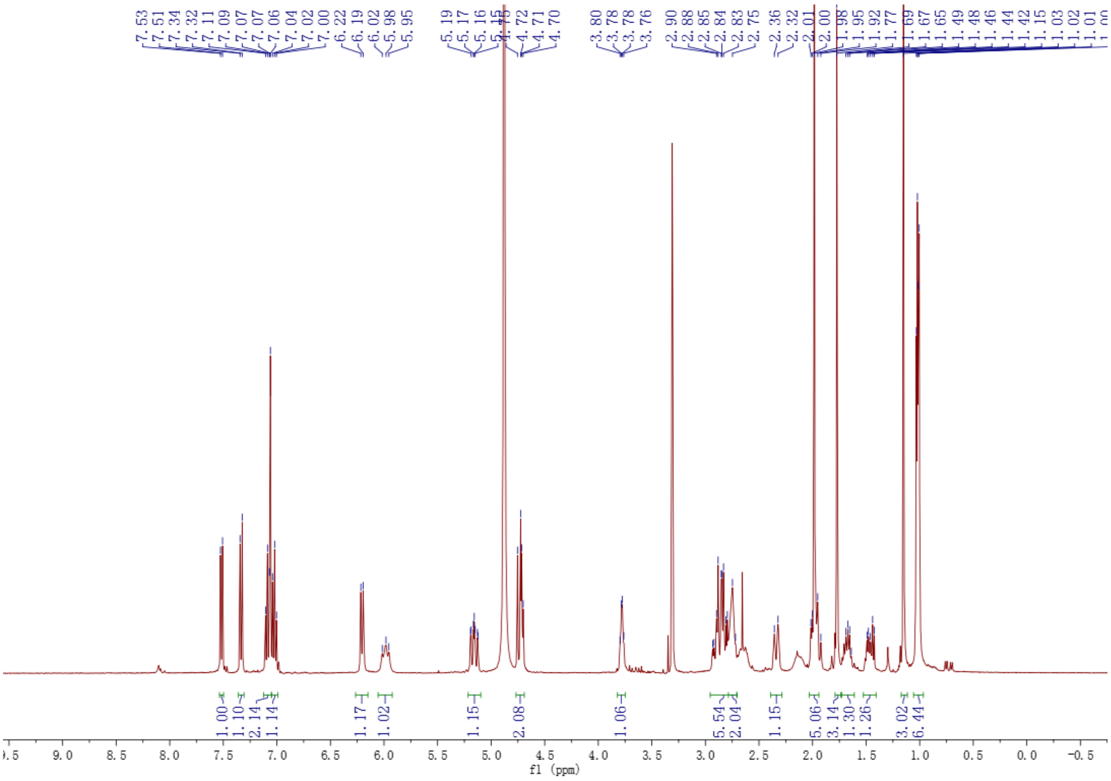

^{13}C NMR and DEPT spectra of compound **2**

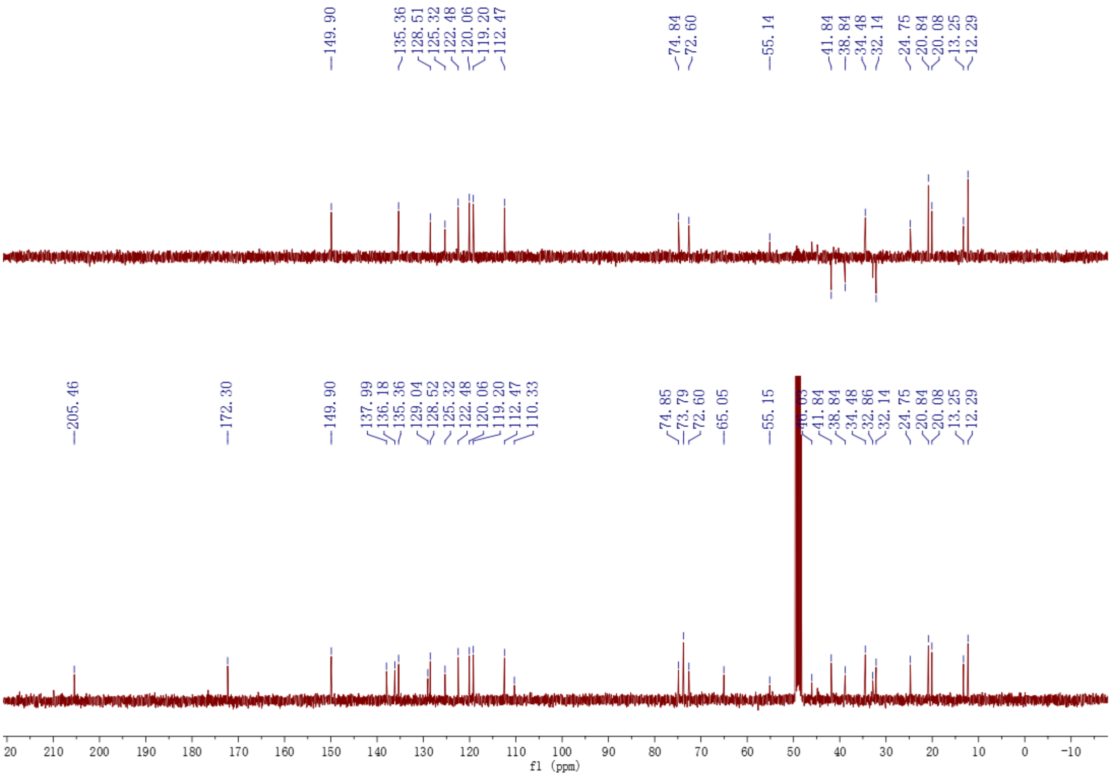

HSQC spectrum of compound **2**

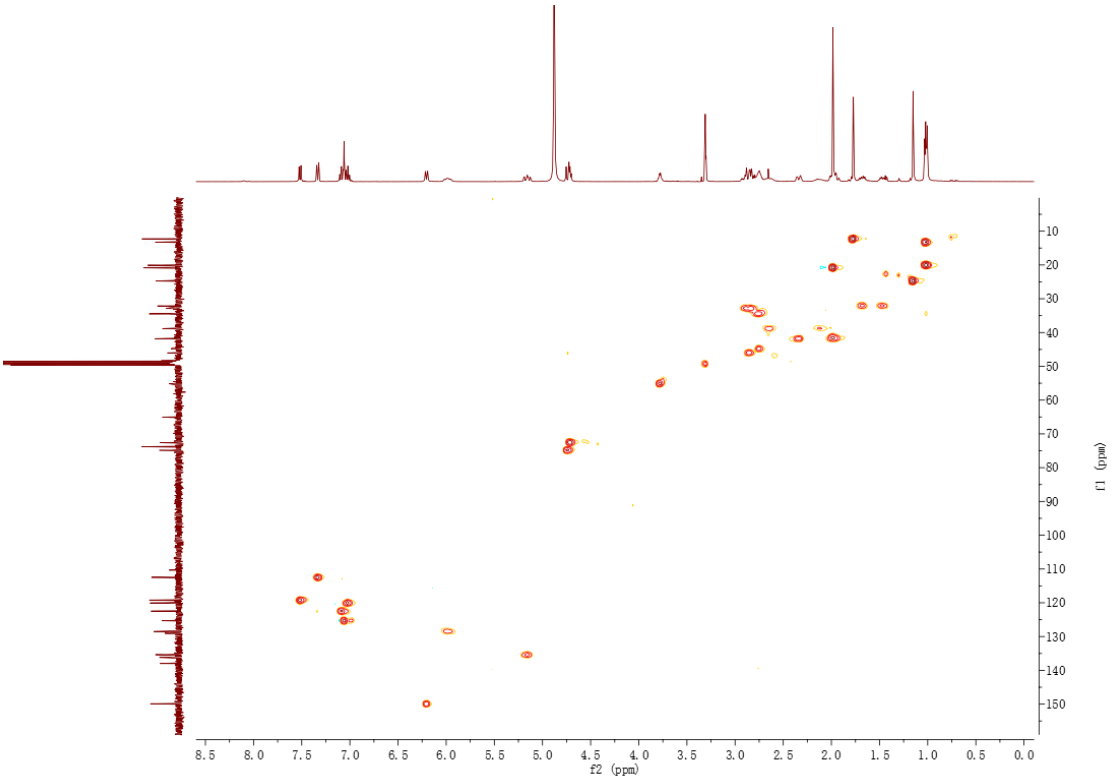

HMBC spectrum of compound **2**

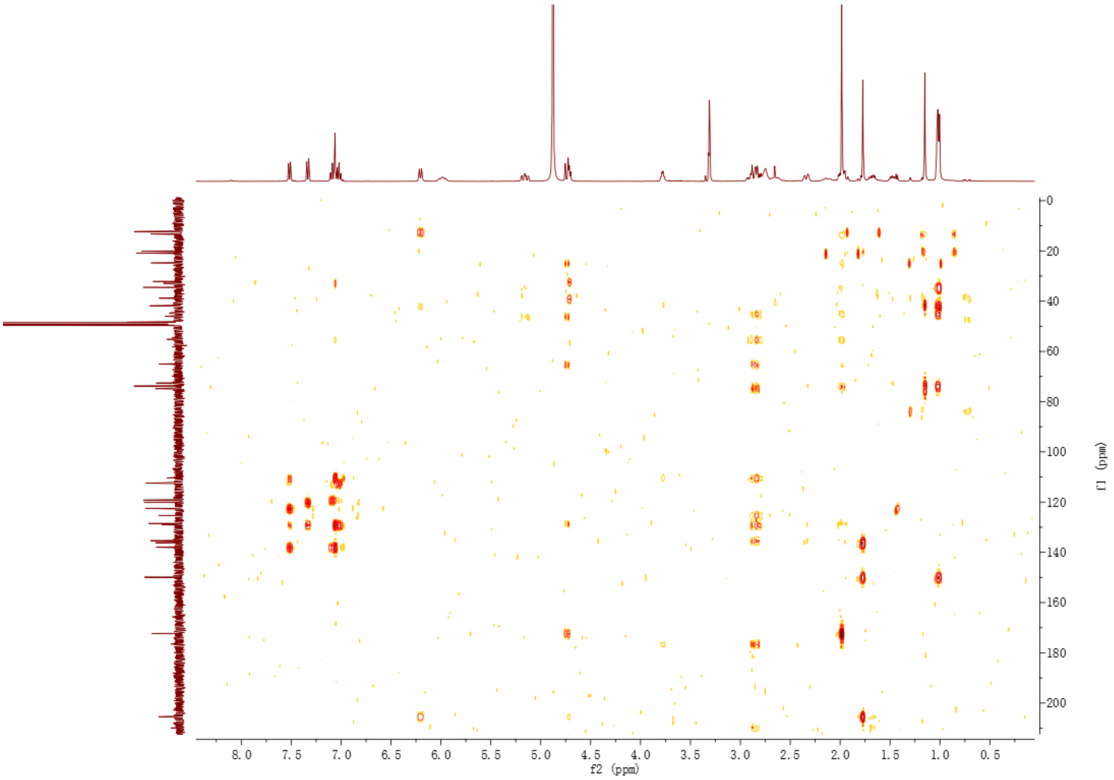

^{1}H–^{1}H COSY spectrum of compound **2**

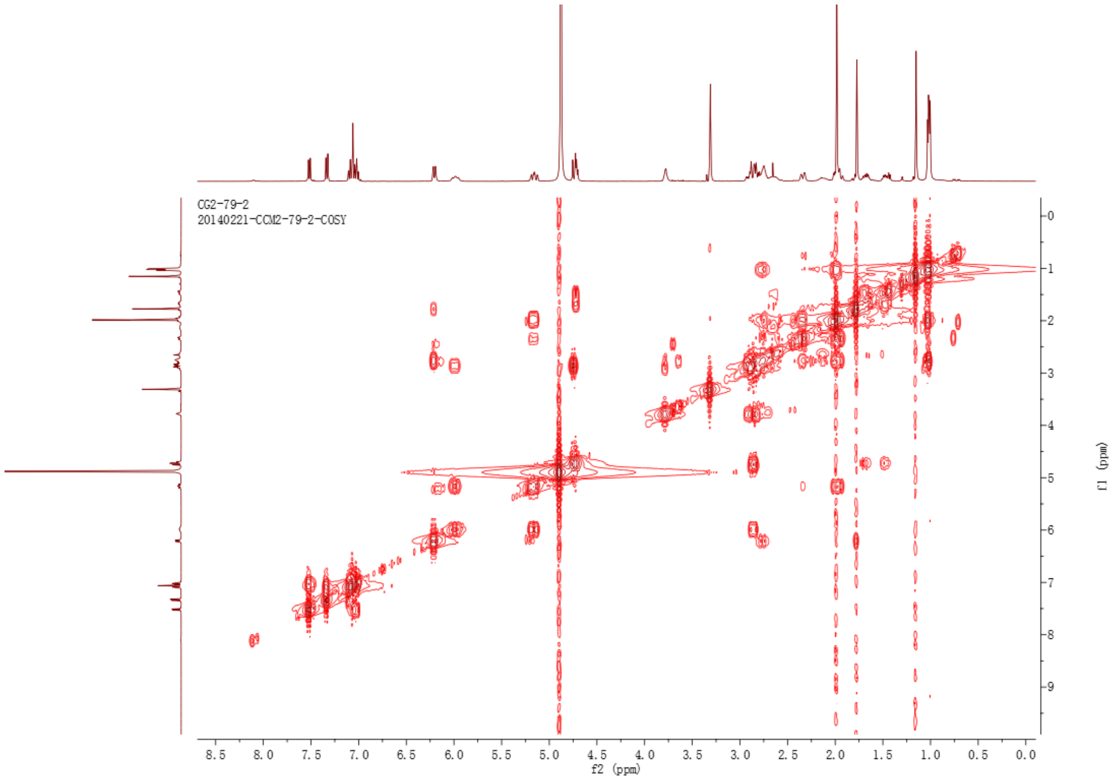

NOESY spectrum of compound **2**

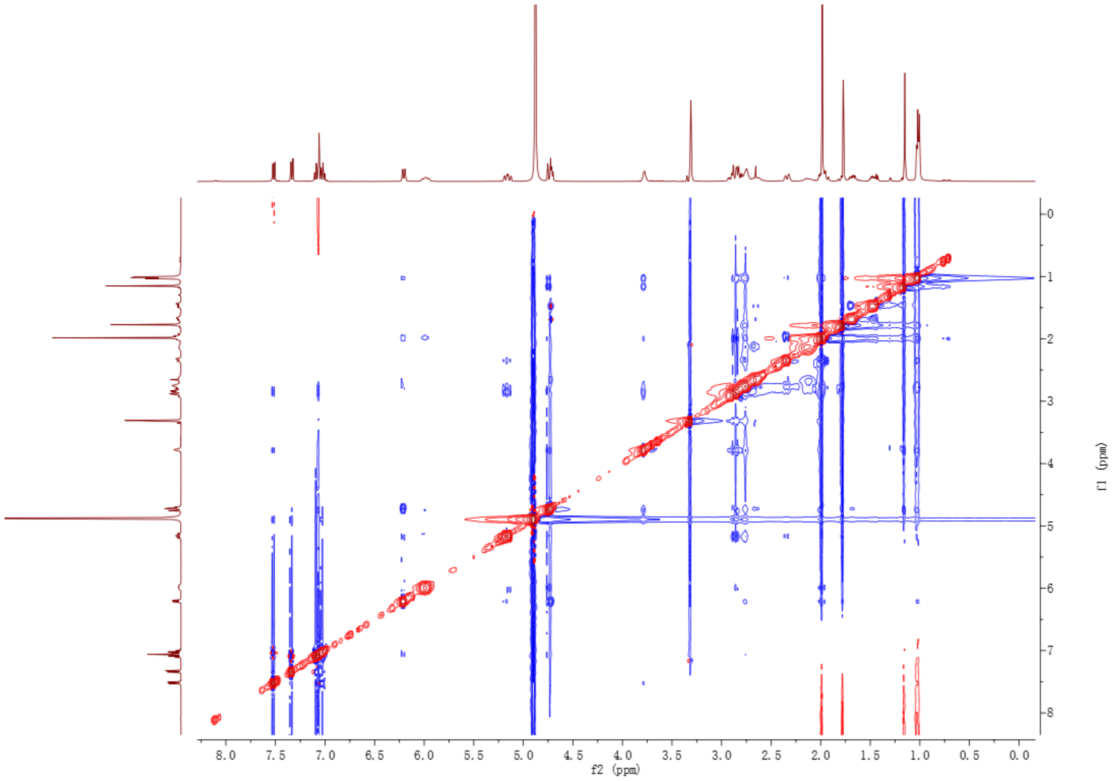

UV spectrum of compound **3**

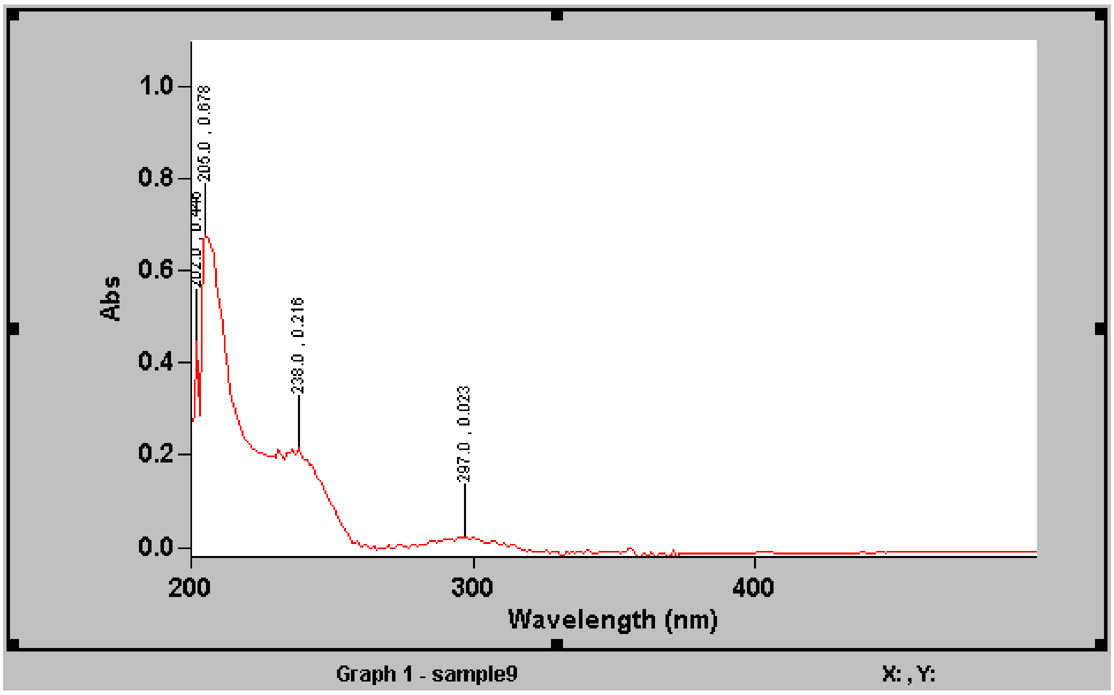

IR spectrum of compound **3**

E:\同济医学院\张勇慧\20140617\001.6

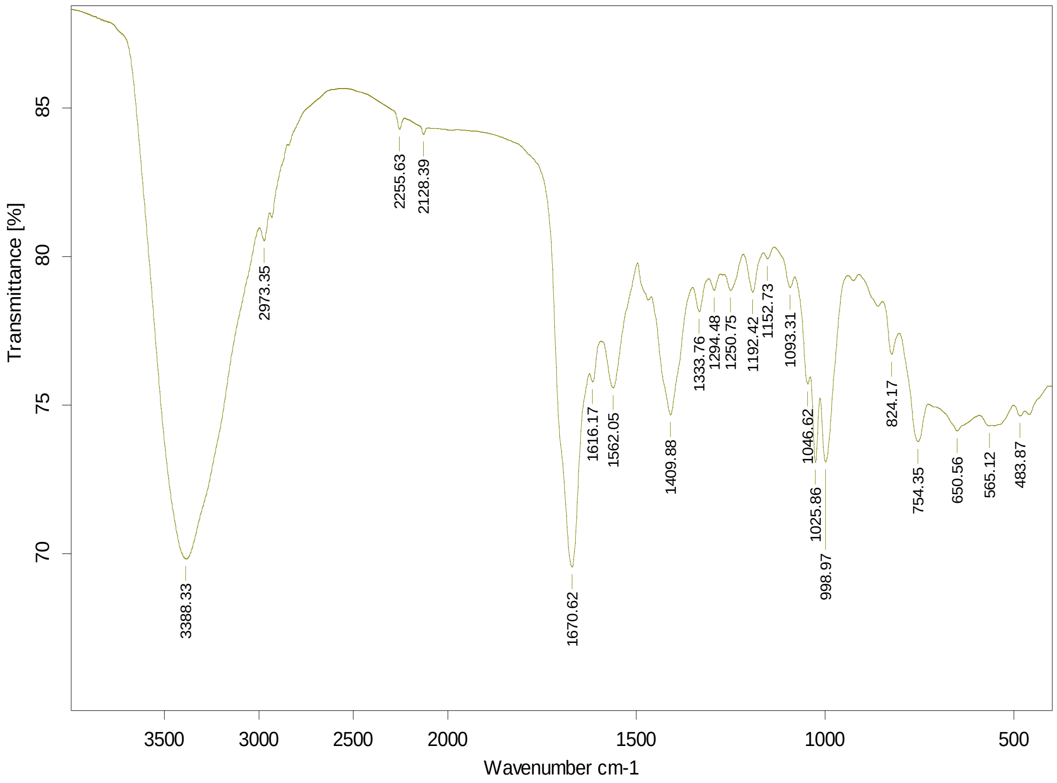

HRESIMS spectrum of compound **3**

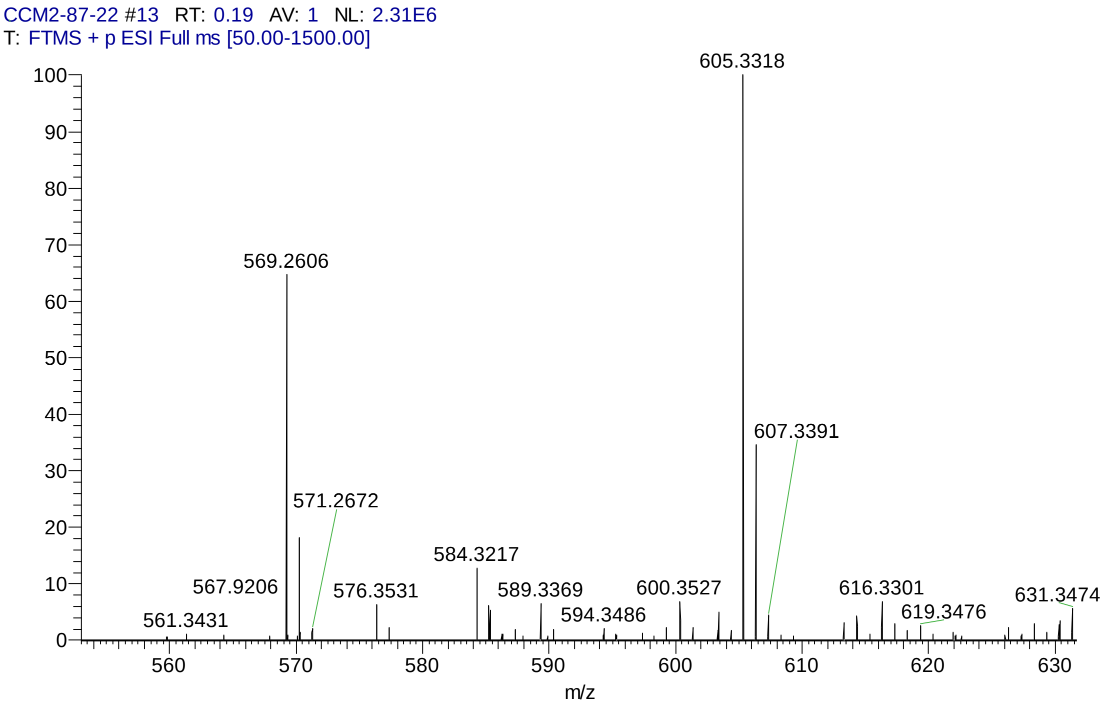

^{1}H NMR spectrum of compound **3**

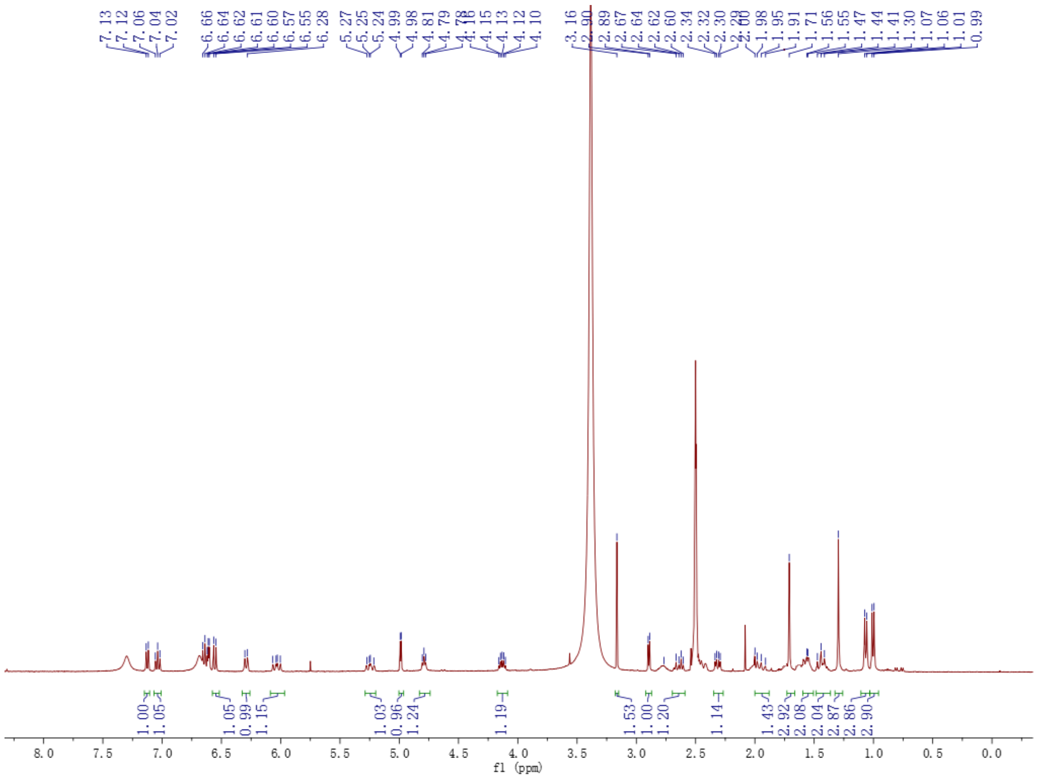

^{13}C NMR and DEPT spectra of compound **3**

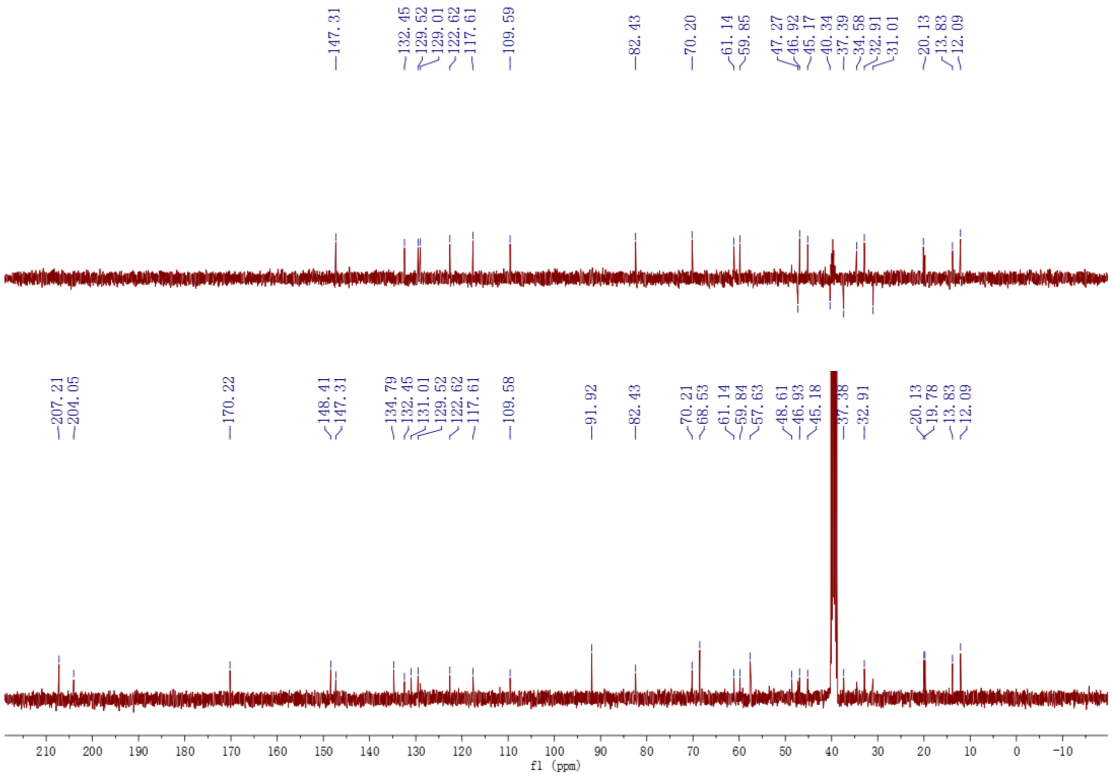

HSQC spectrum of compound **3**

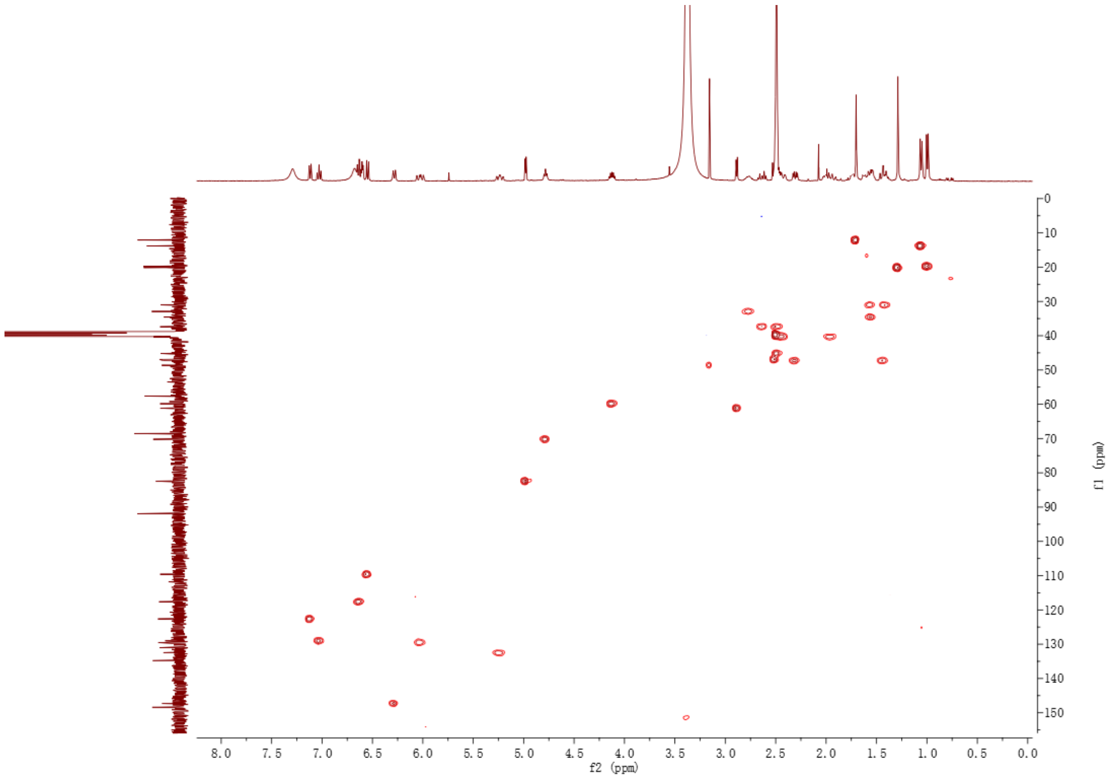

HMBC spectrum of compound **3**

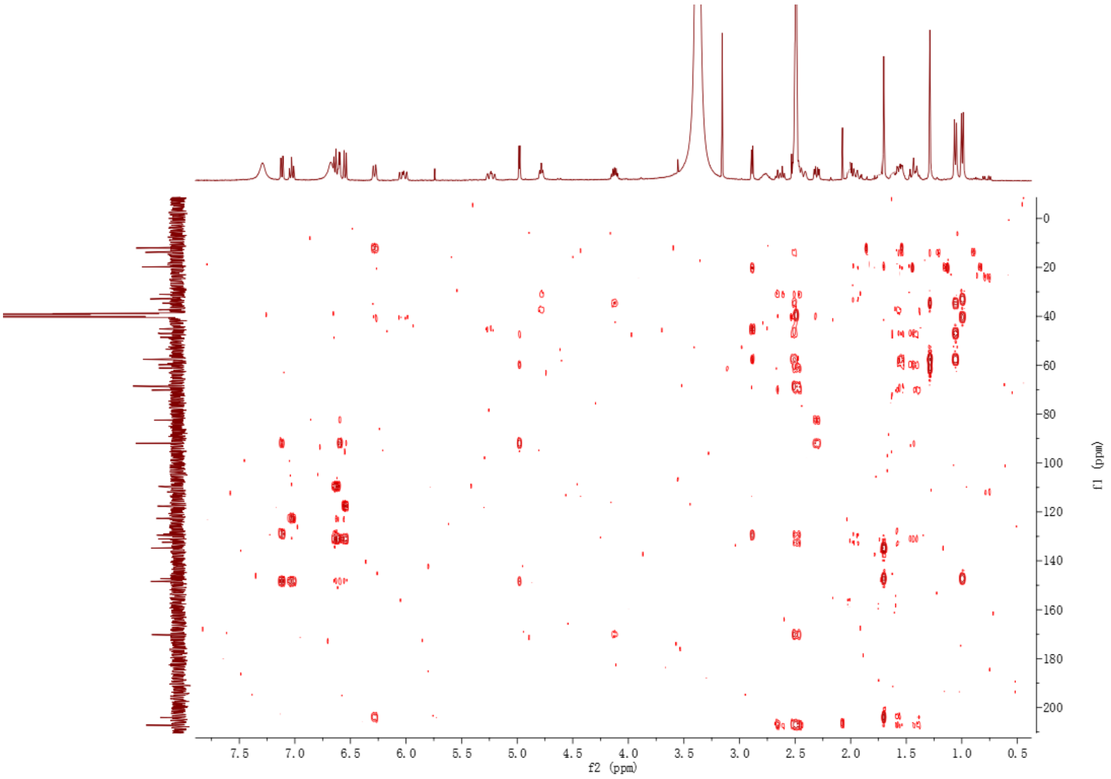

^1H–^1H COSY spectrum of compound **3**

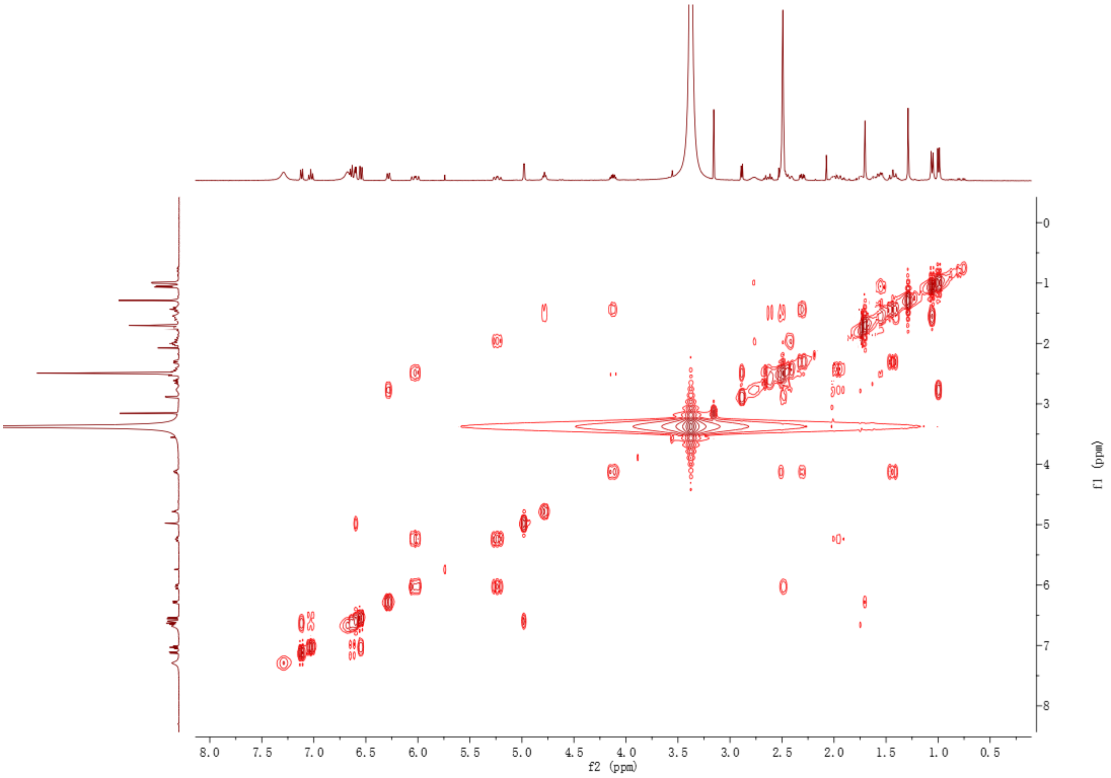

NOESY spectrum of compound **3**

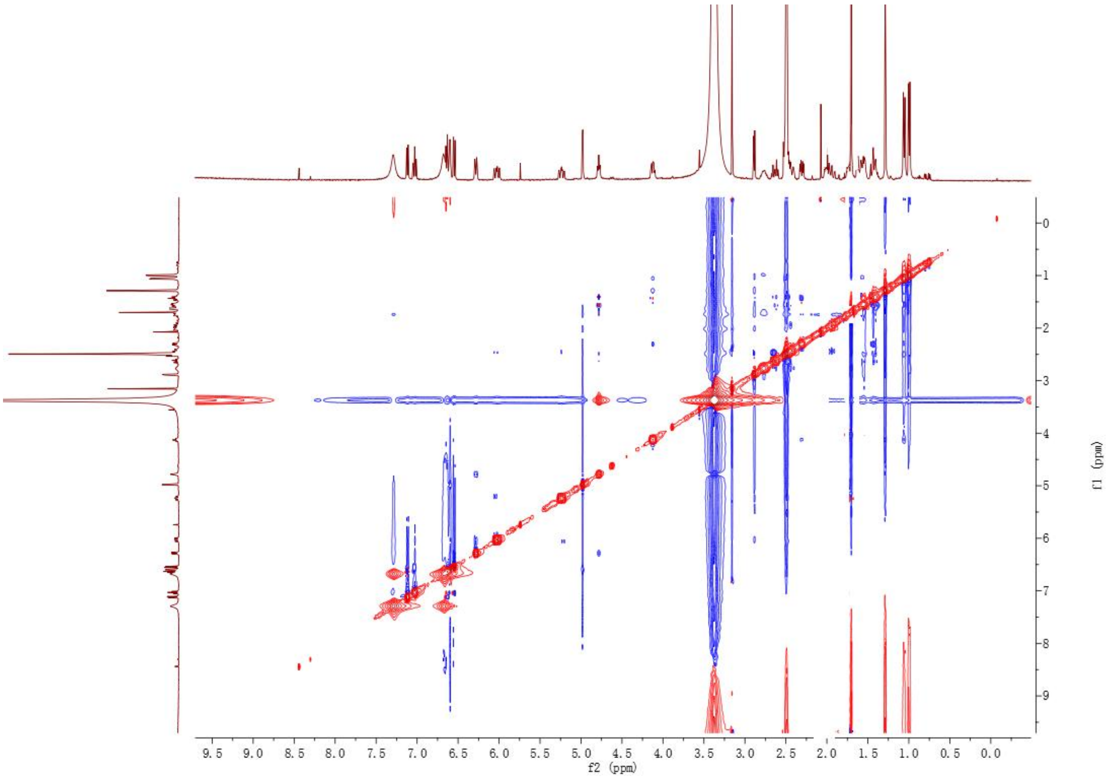

UV spectrum of compound **4**

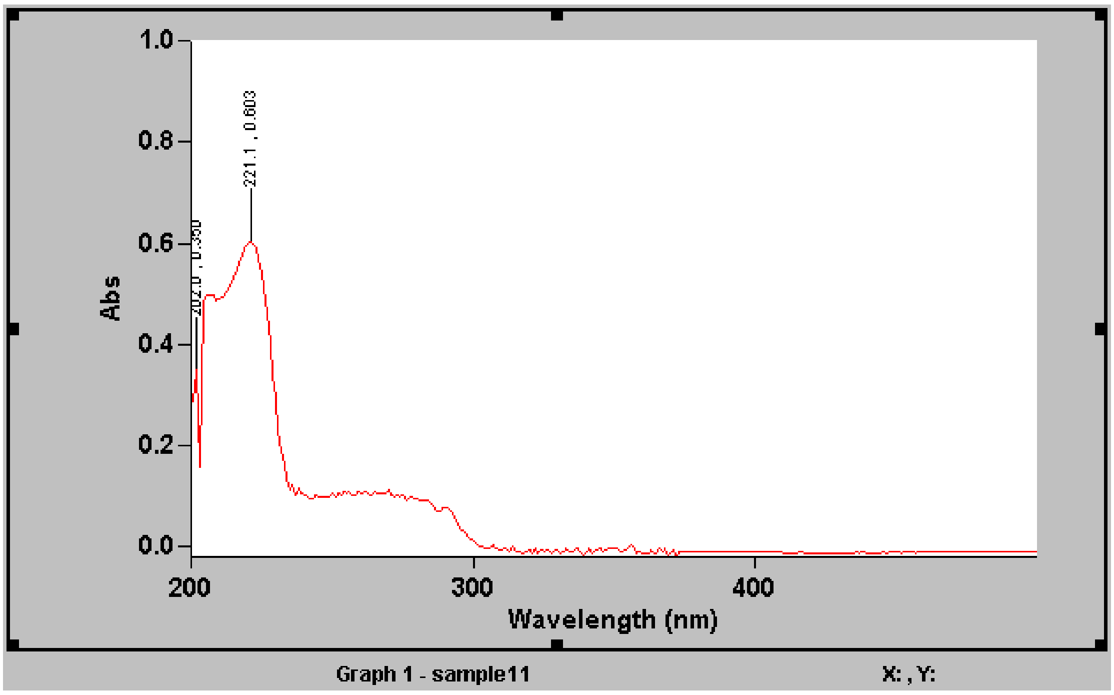

IR spectrum of compound **4**

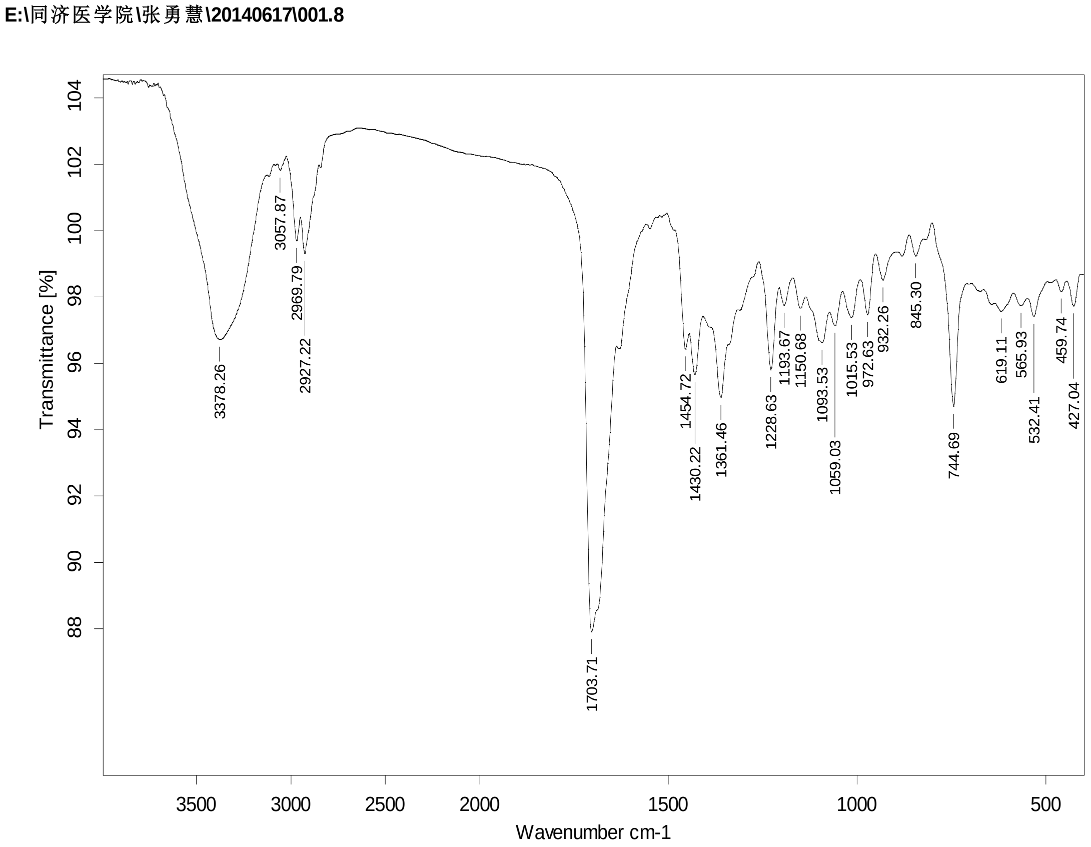

HRESIMS spectrum of compound **4**

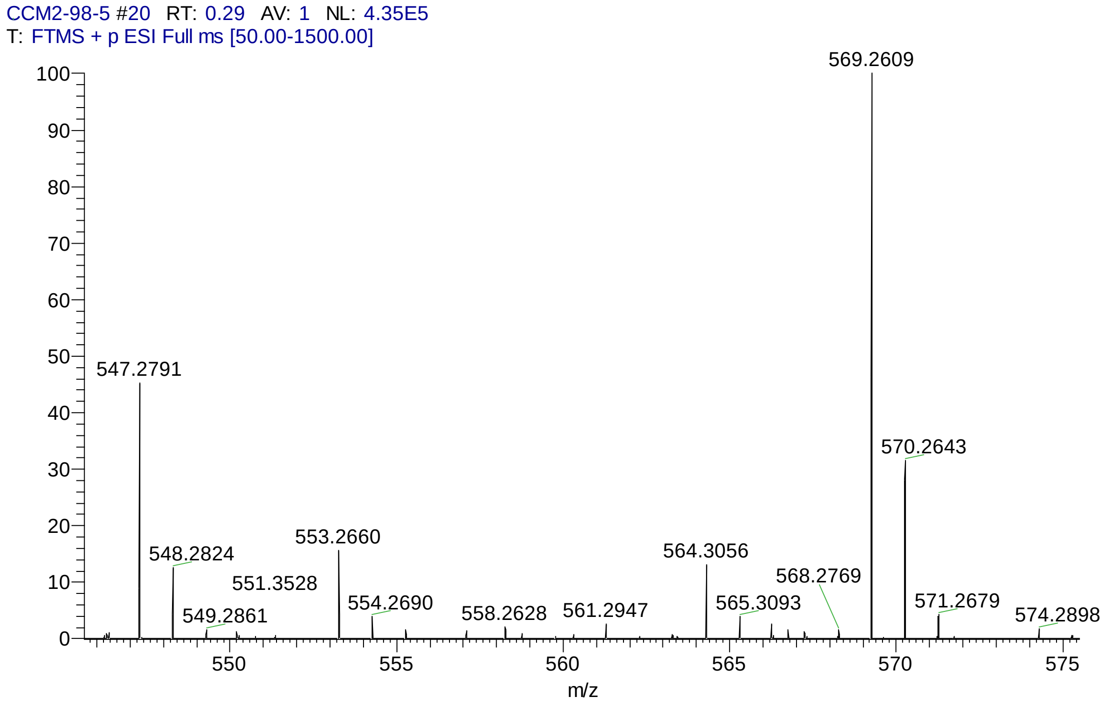

^{1}H NMR spectrum of compound **4**

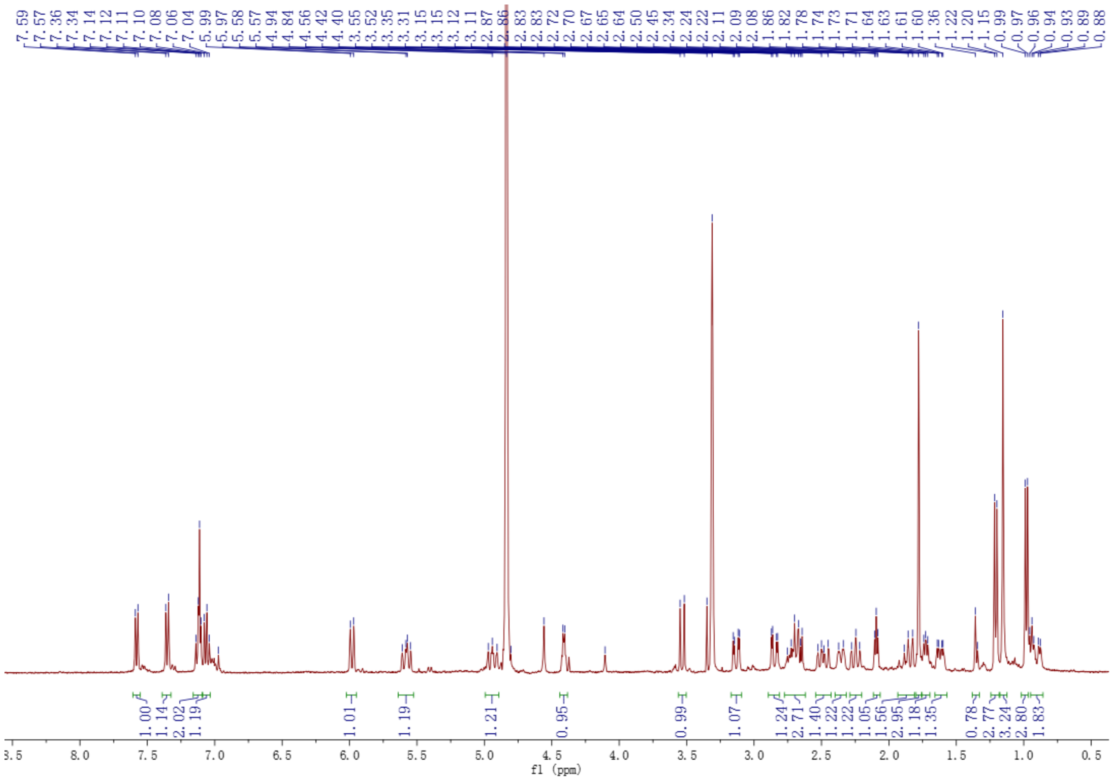

^{13}C NMR and DEPT spectra of compound **4**

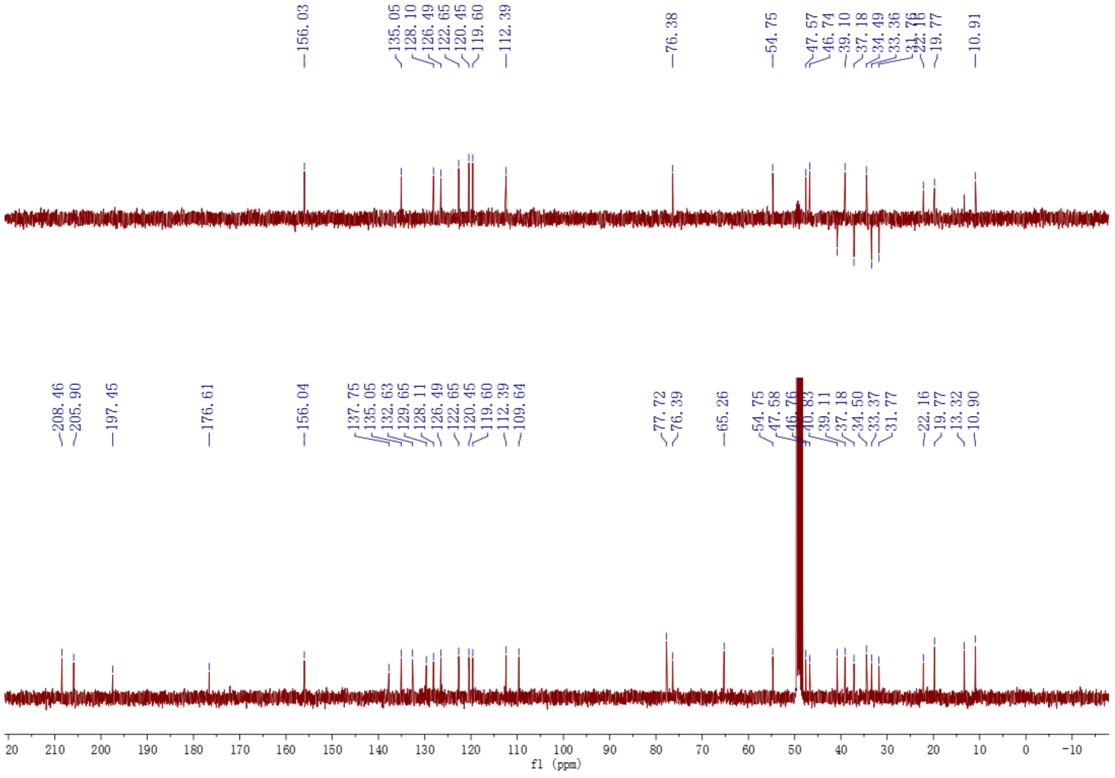

HSQC spectrum of compound **4**

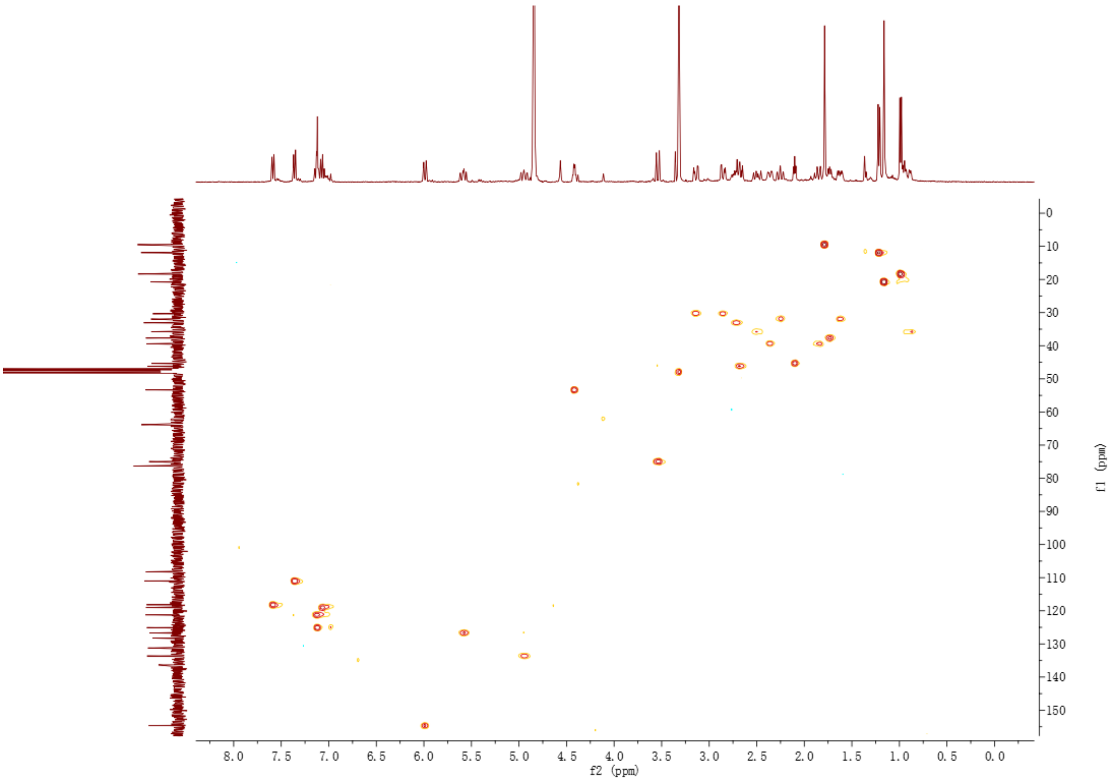

HMBC spectrum of compound **4**

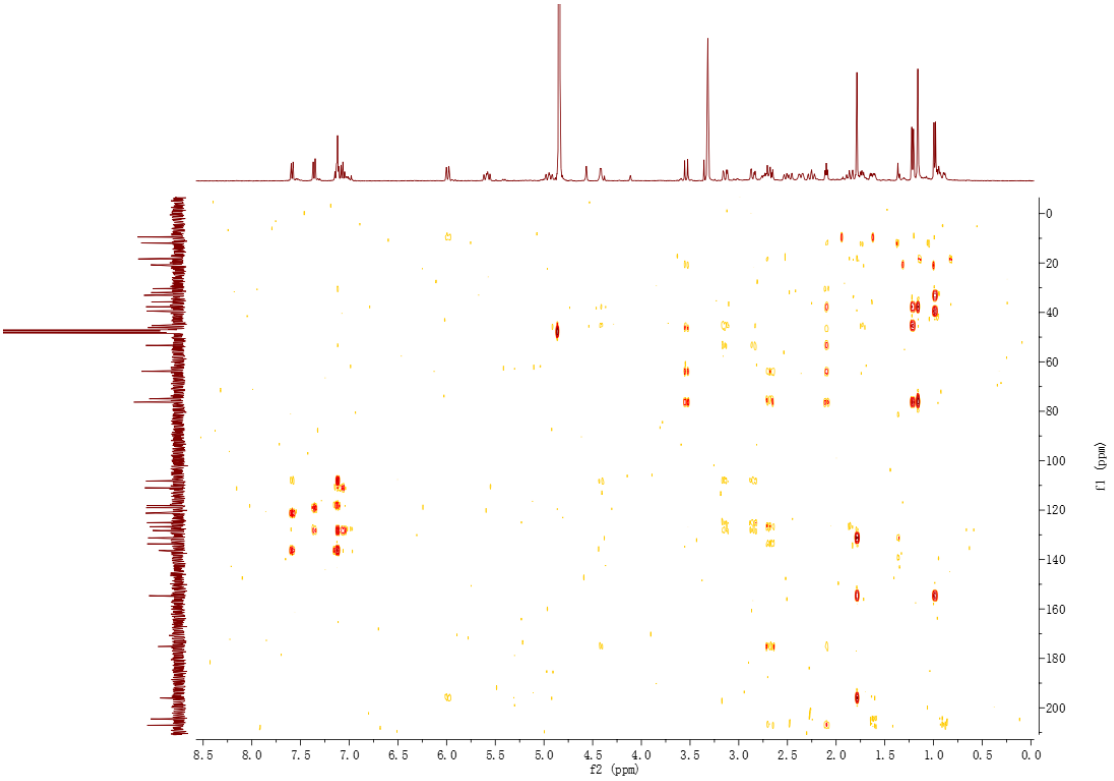

^{1}H–^{1}H COSY spectrum of compound **4**

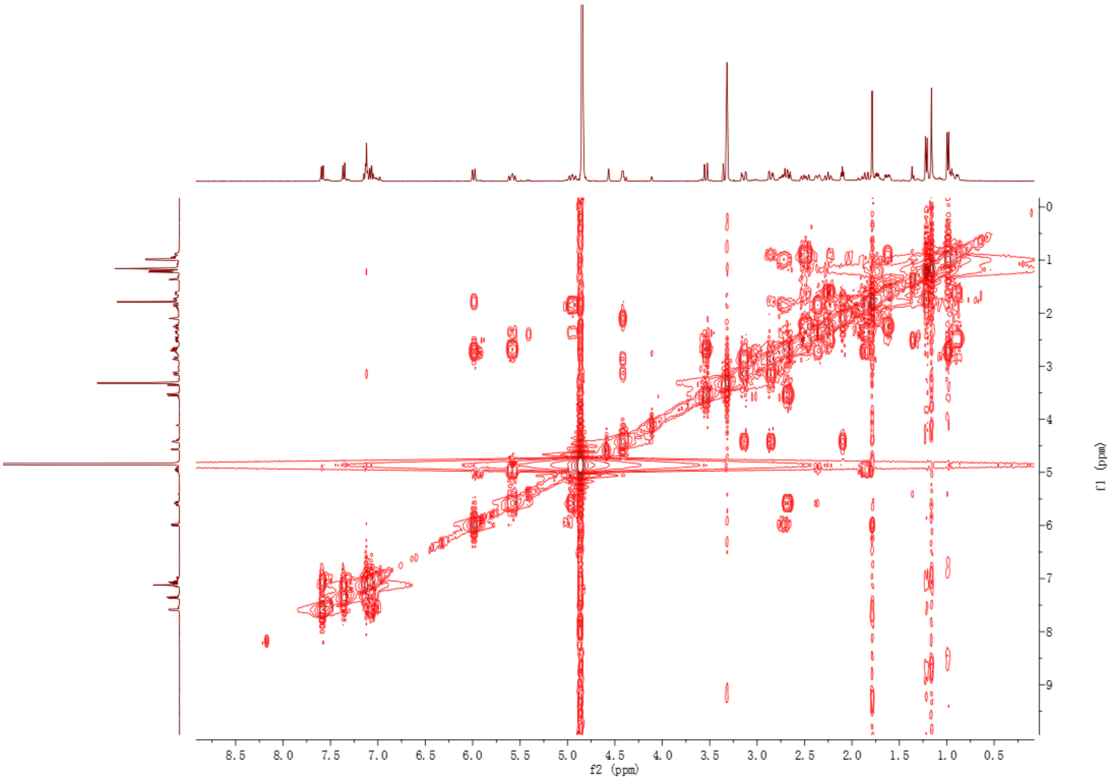

NOESY spectrum of compound **4**

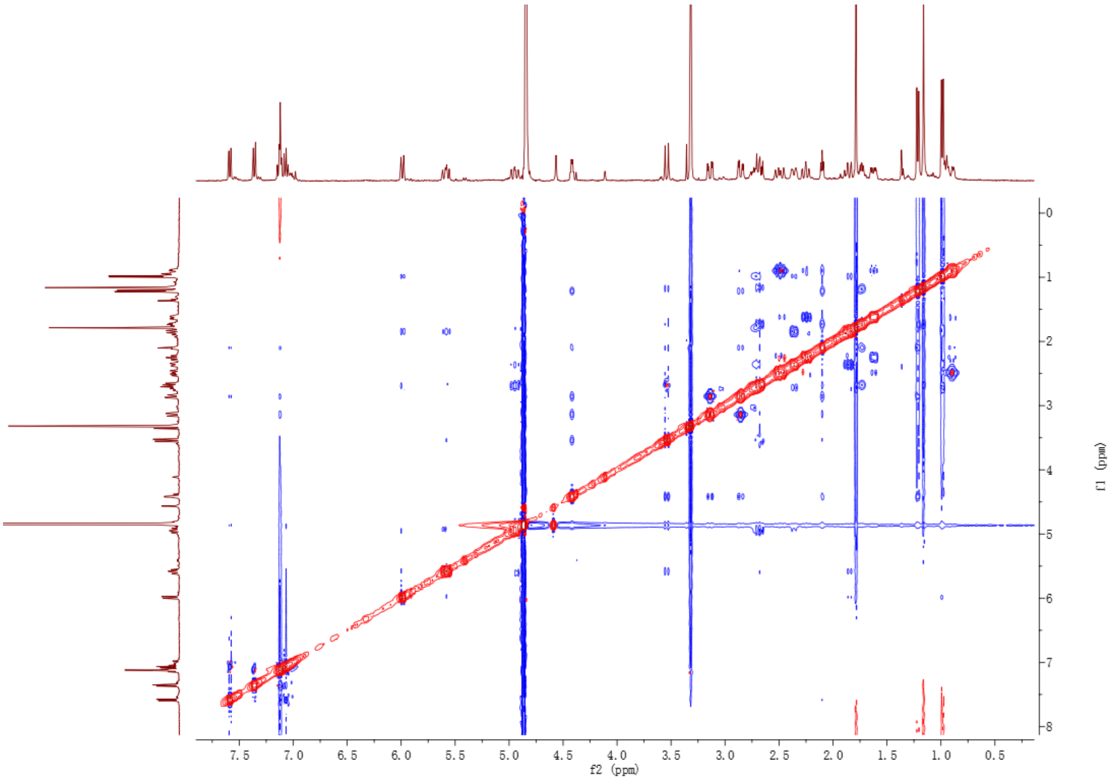

UV spectrum of compound **5**

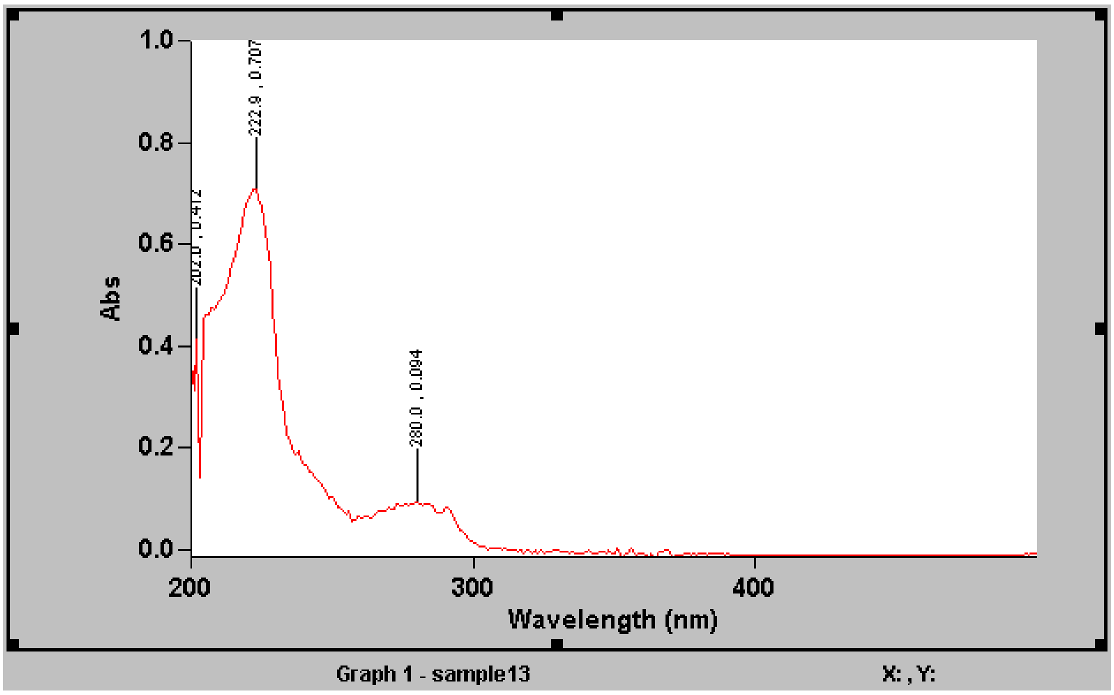

IR spectrum of compound **5**

E:\同济医学院\张勇慧\20131217\2-55-12.0

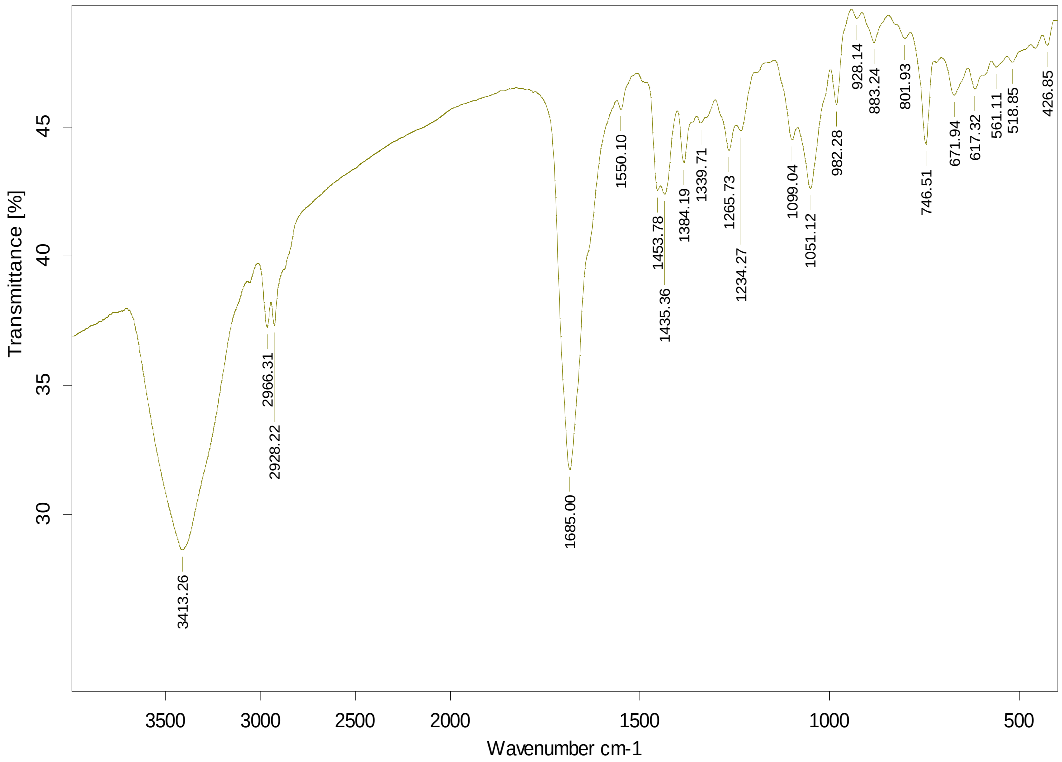

HRESIMS spectrum of compound **5**

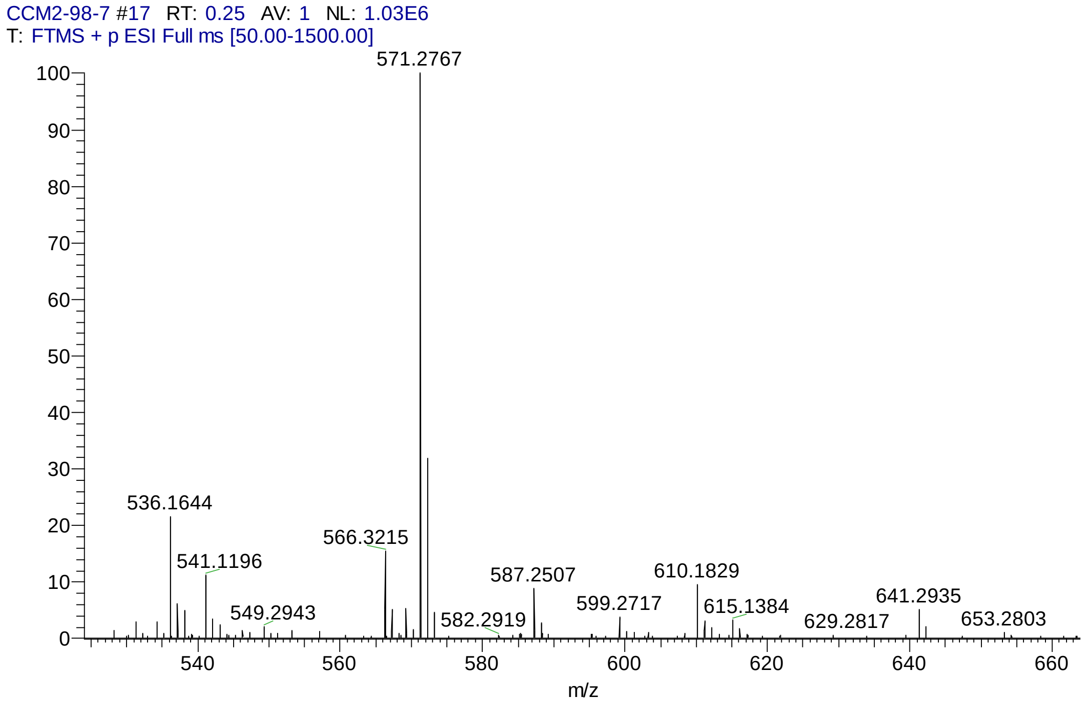

^{1}H NMR spectrum of compound **5**

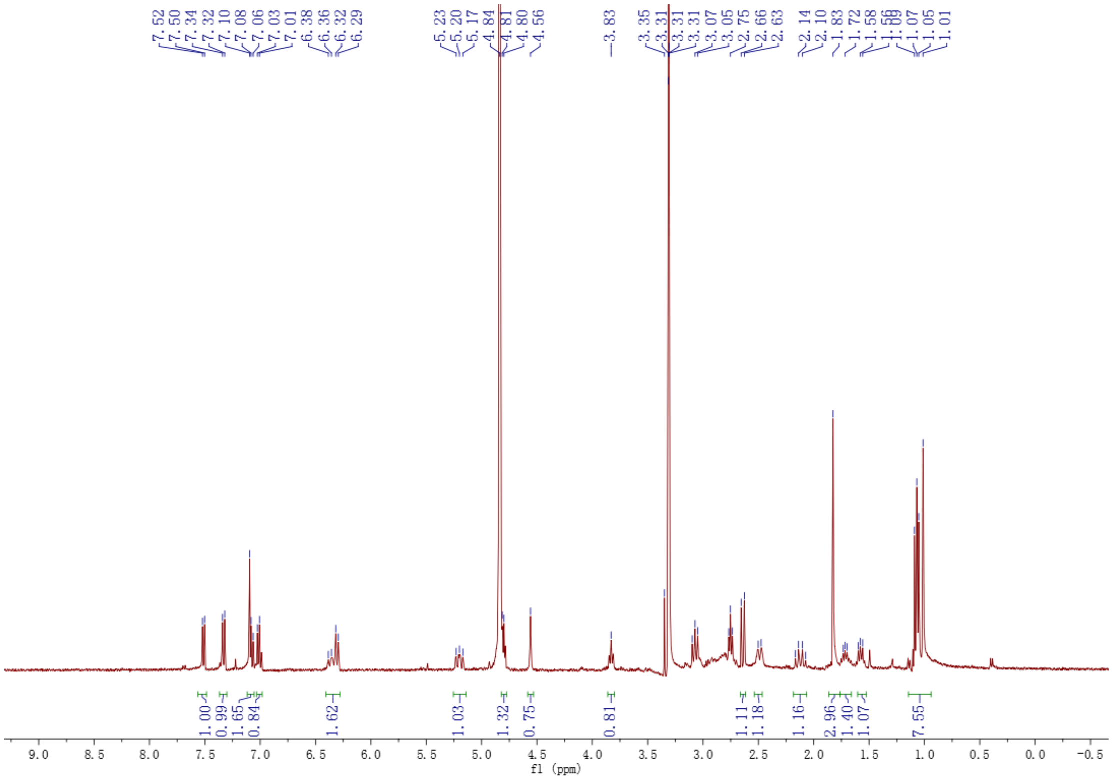

^{13}C NMR and DEPT spectra of compound **5**

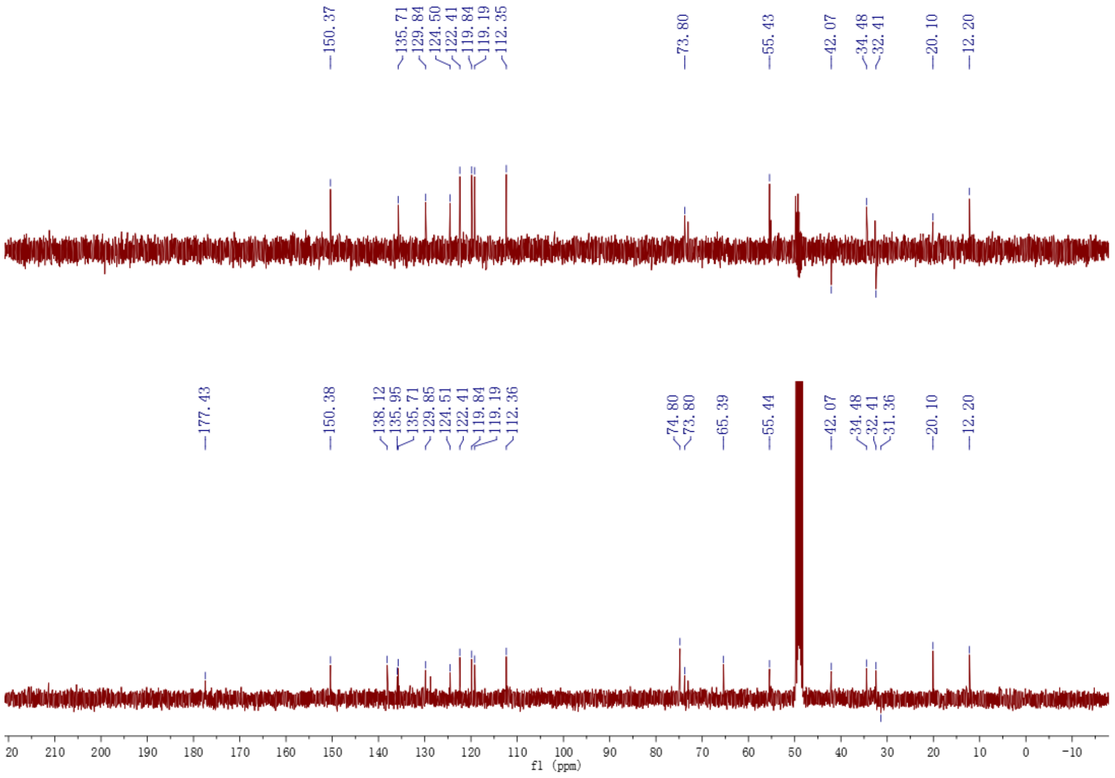

HSQC spectrum of compound **5**

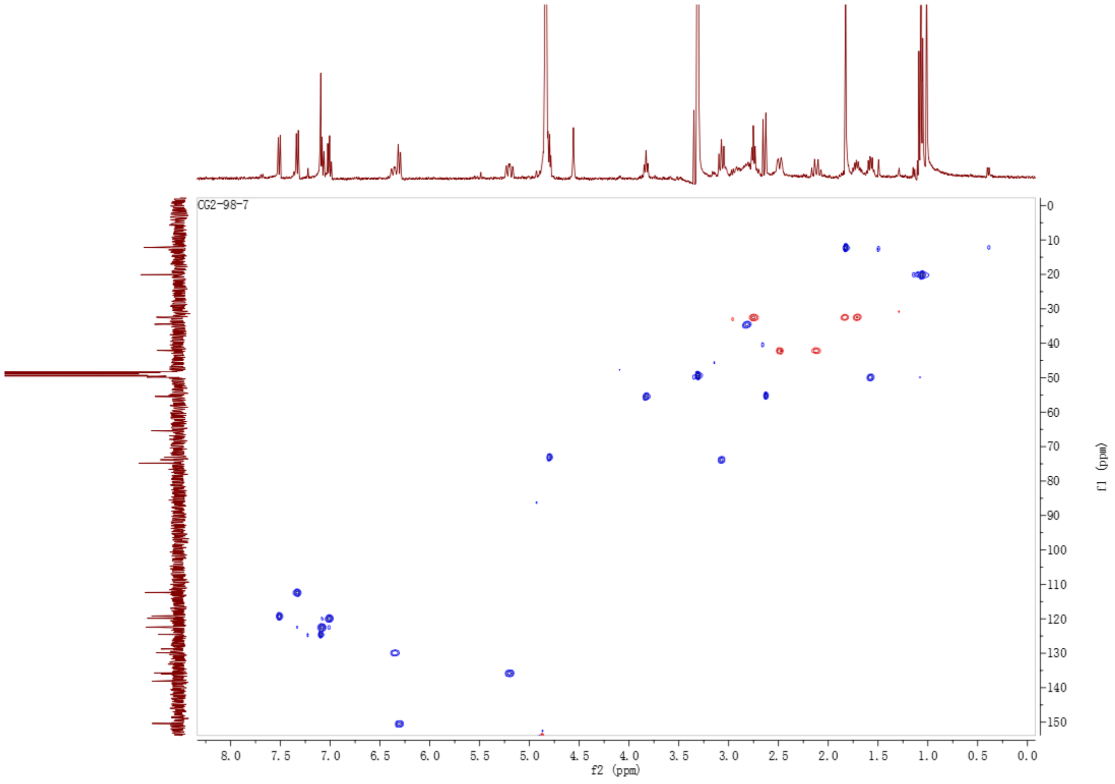

HMBC spectrum of compound **5**

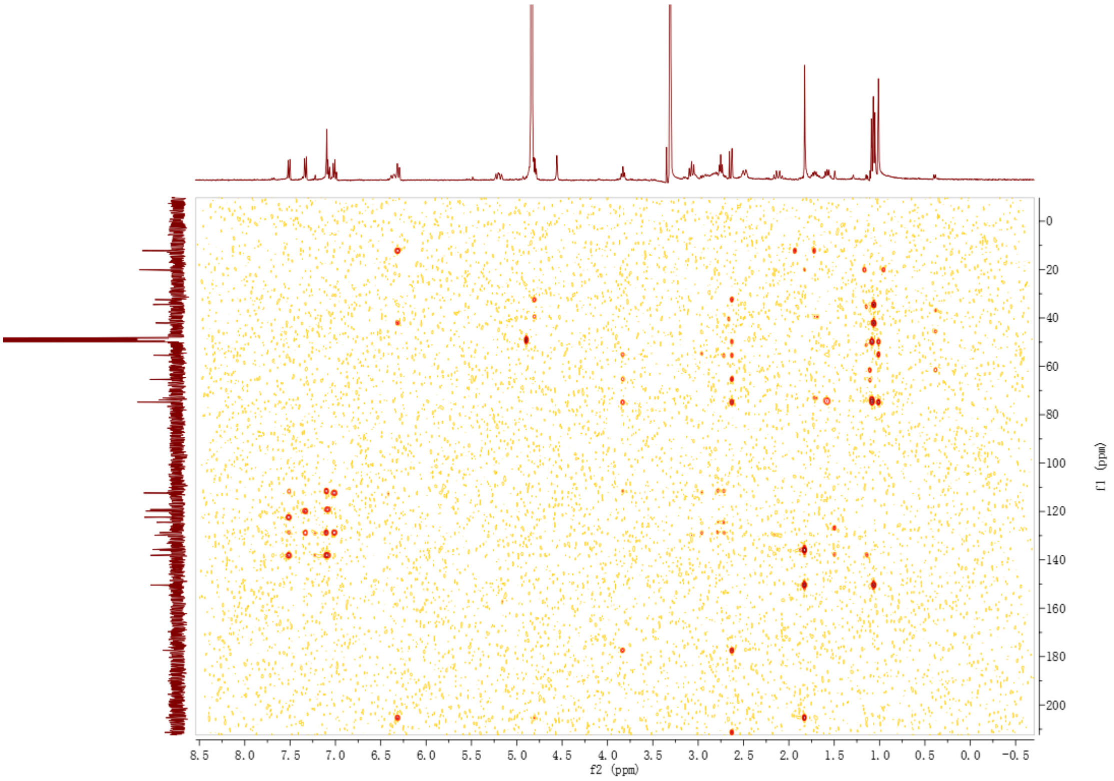

^1H–^1H COSY spectrum of compound **5**

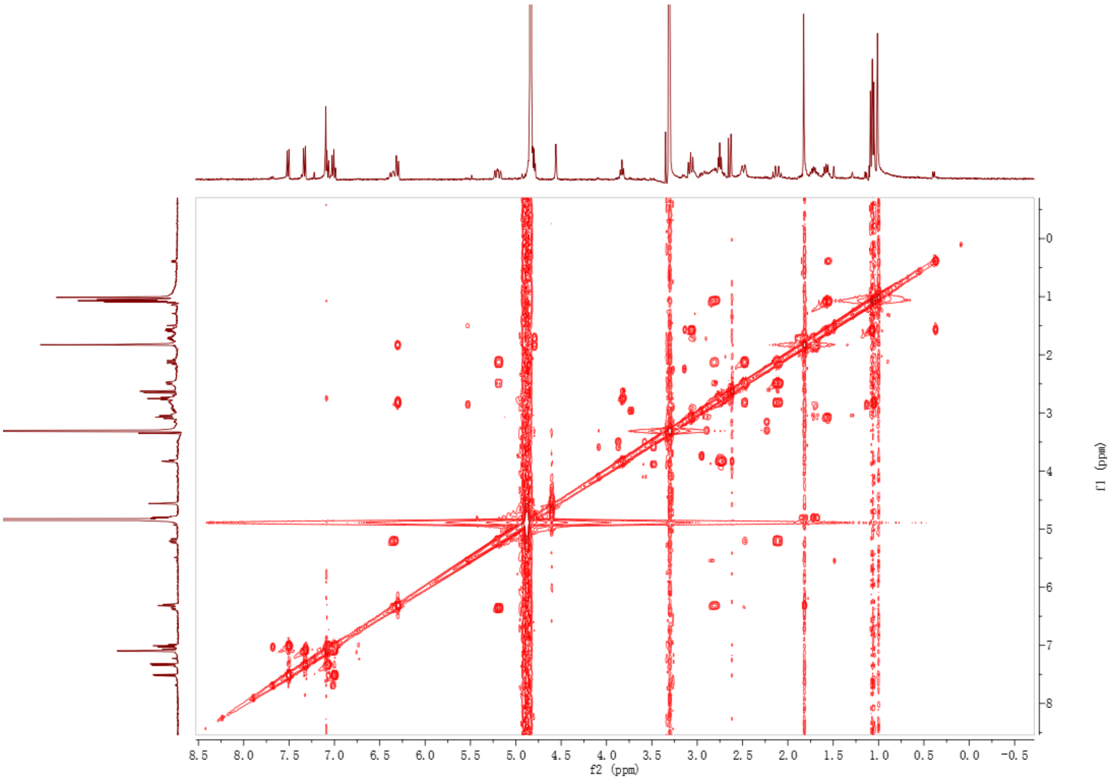

NOESY spectrum of compound **5**

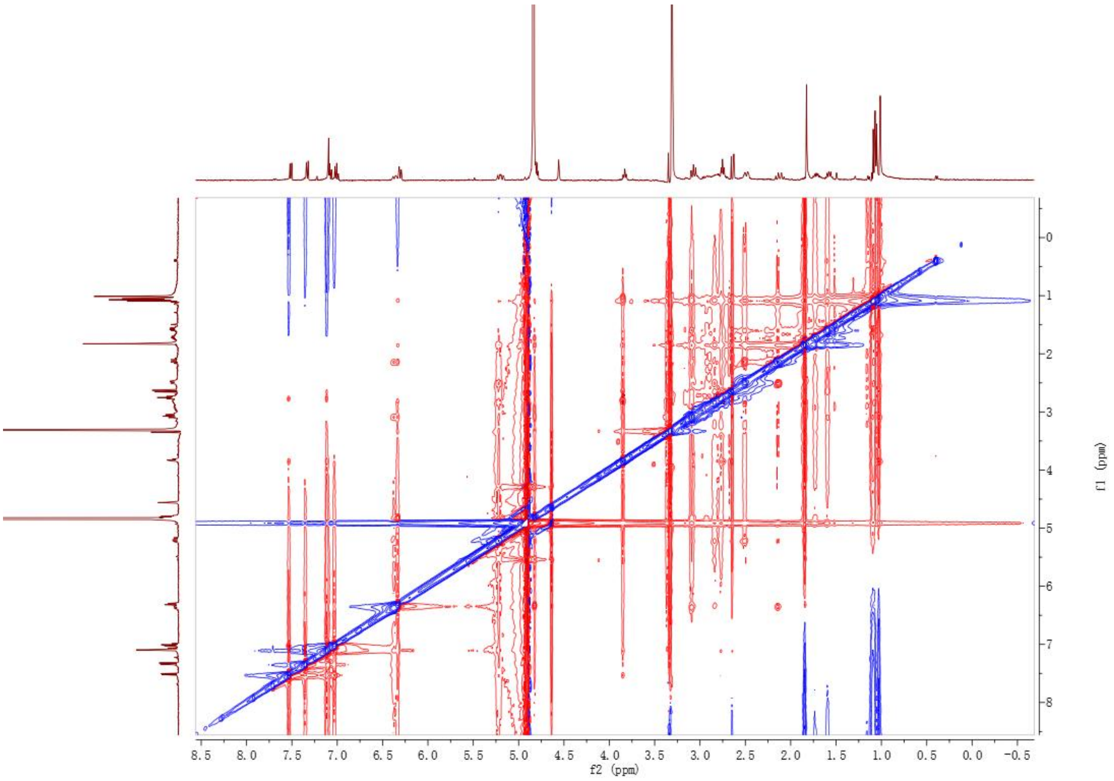

UV spectrum of compound **6**

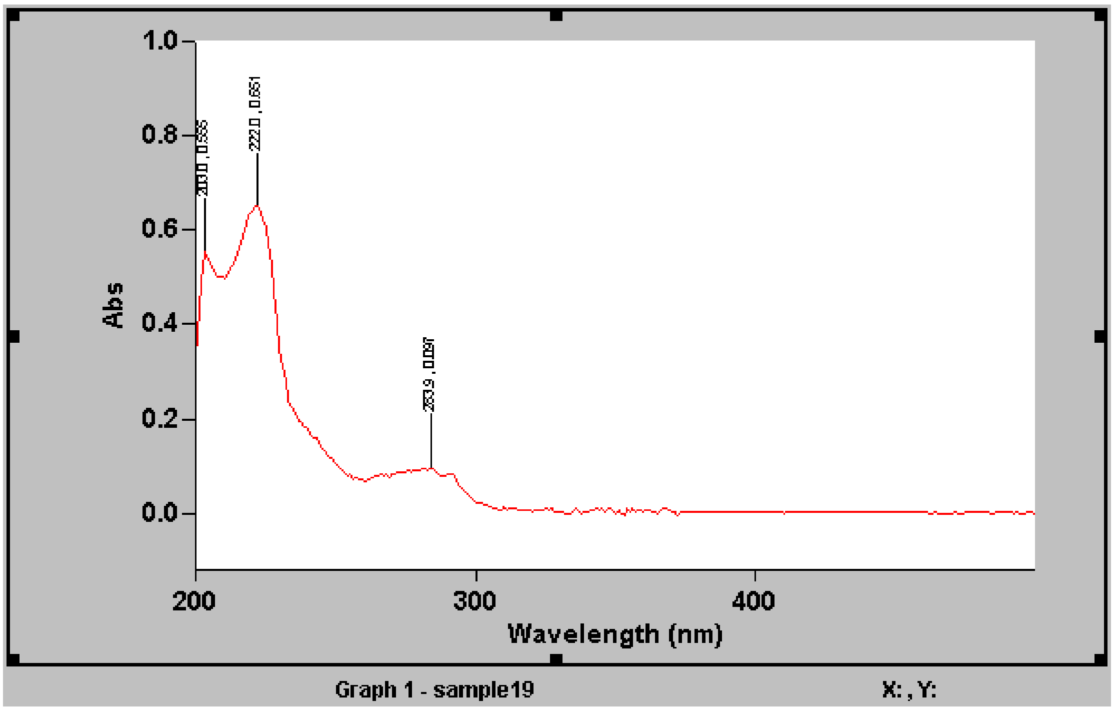

IR spectrum of compound **6**

E:\同济医学院\张勇慧\20131217\2-65-2.0

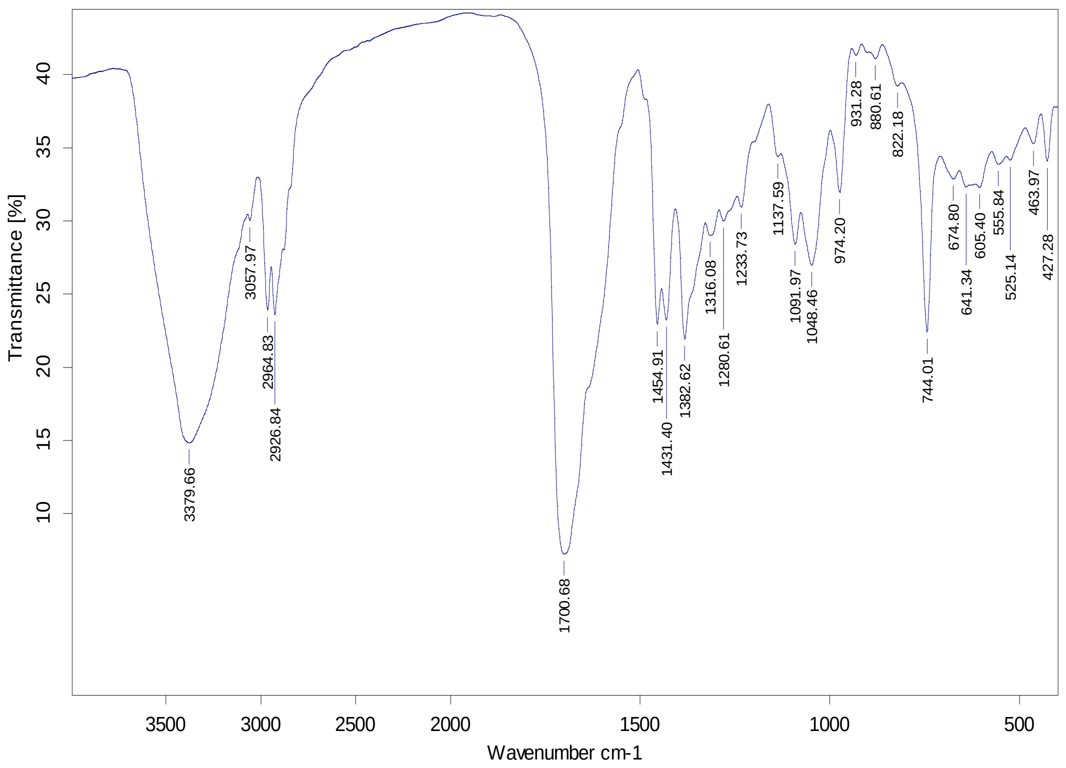

HRESIMS spectrum of compound **6**

CCM2-98-4 #16 RT: 0.24 AV: 1 NL: 1.76E5
T: FTMS + p ESI Full ms [50.00-1500.00]

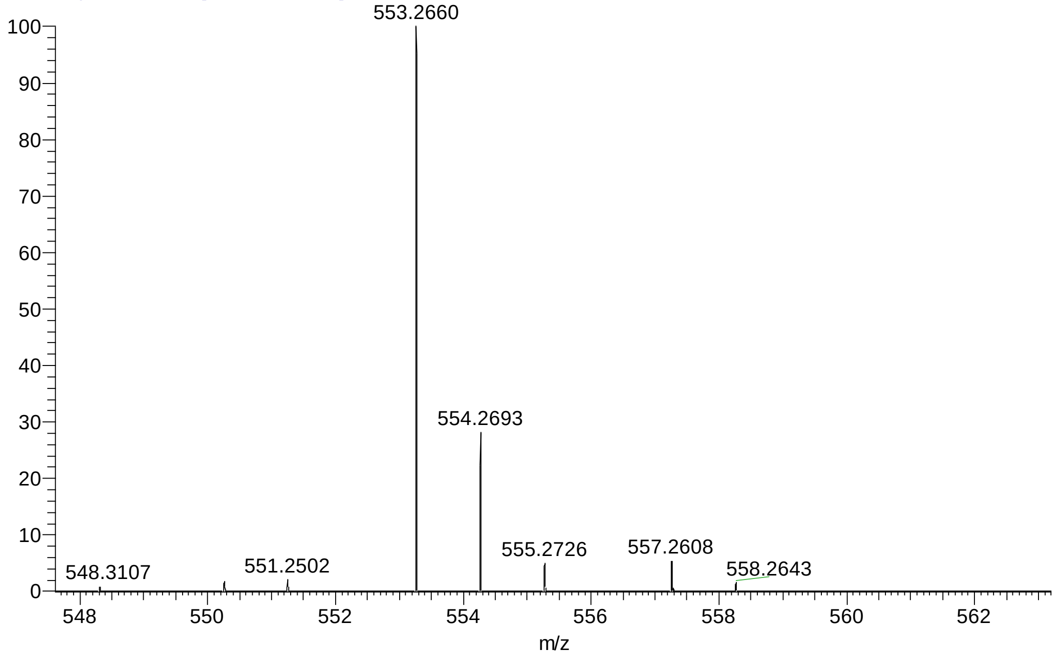

^{1}H NMR spectrum of compound **6**

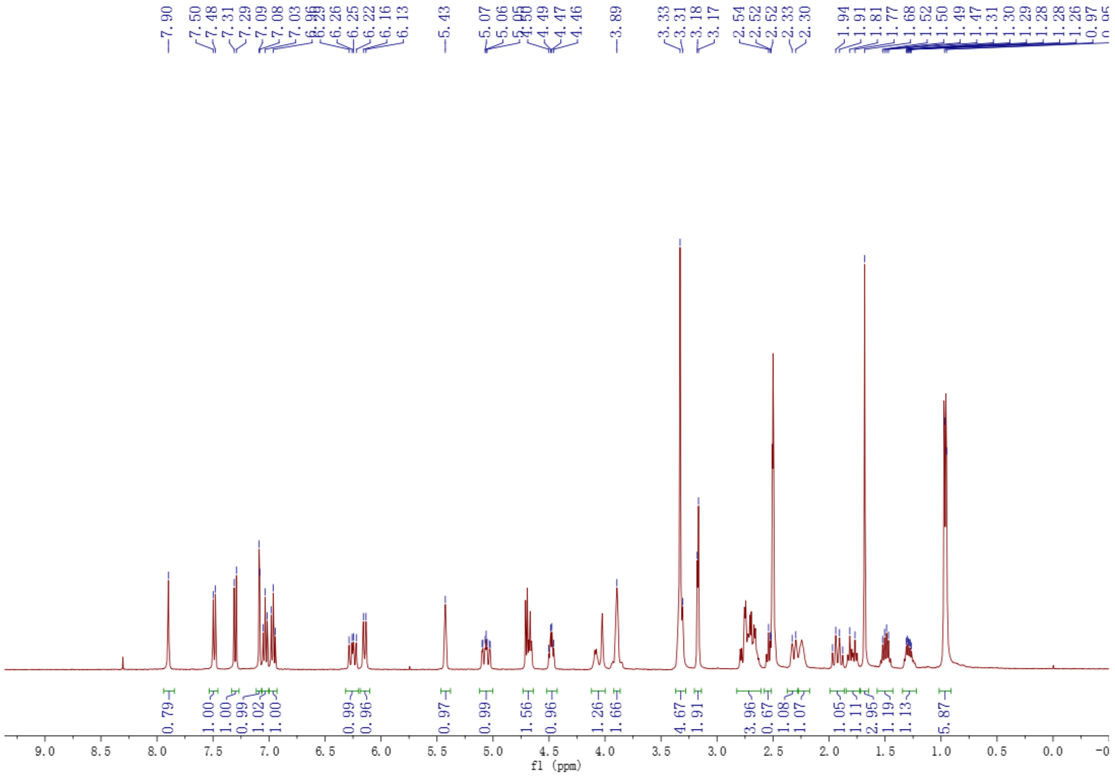

^{13}C NMR and DEPT spectra of compound **6**

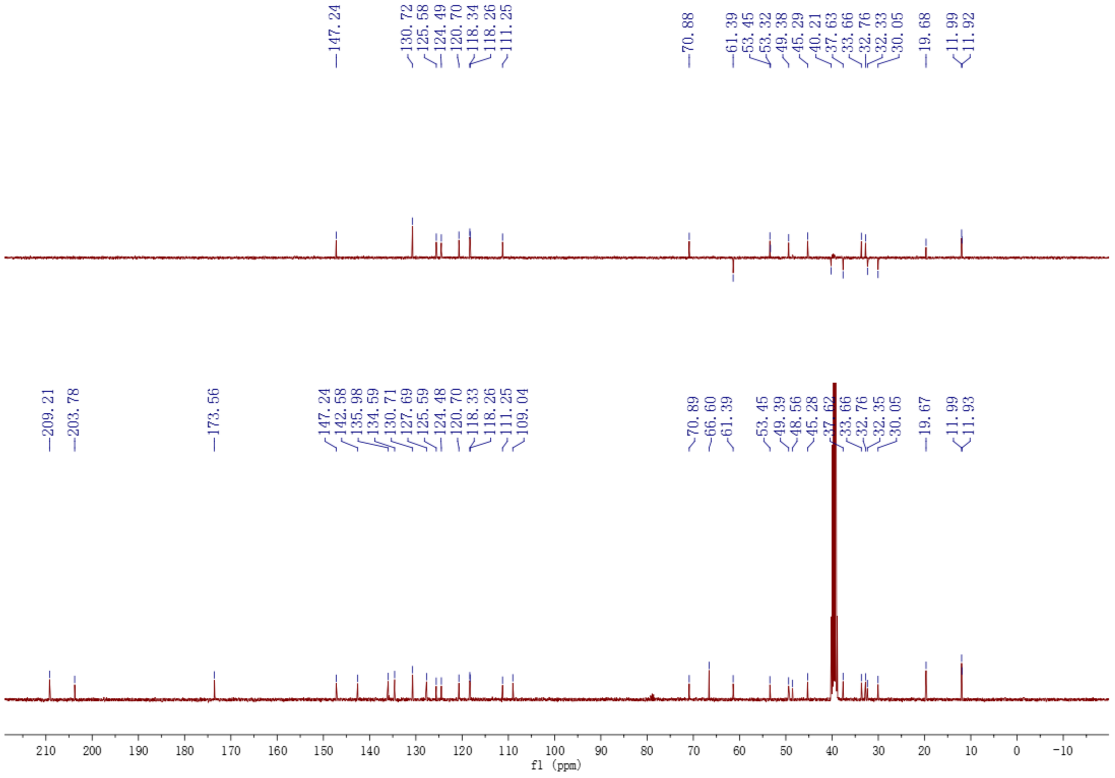

HSQC spectrum of compound **6**

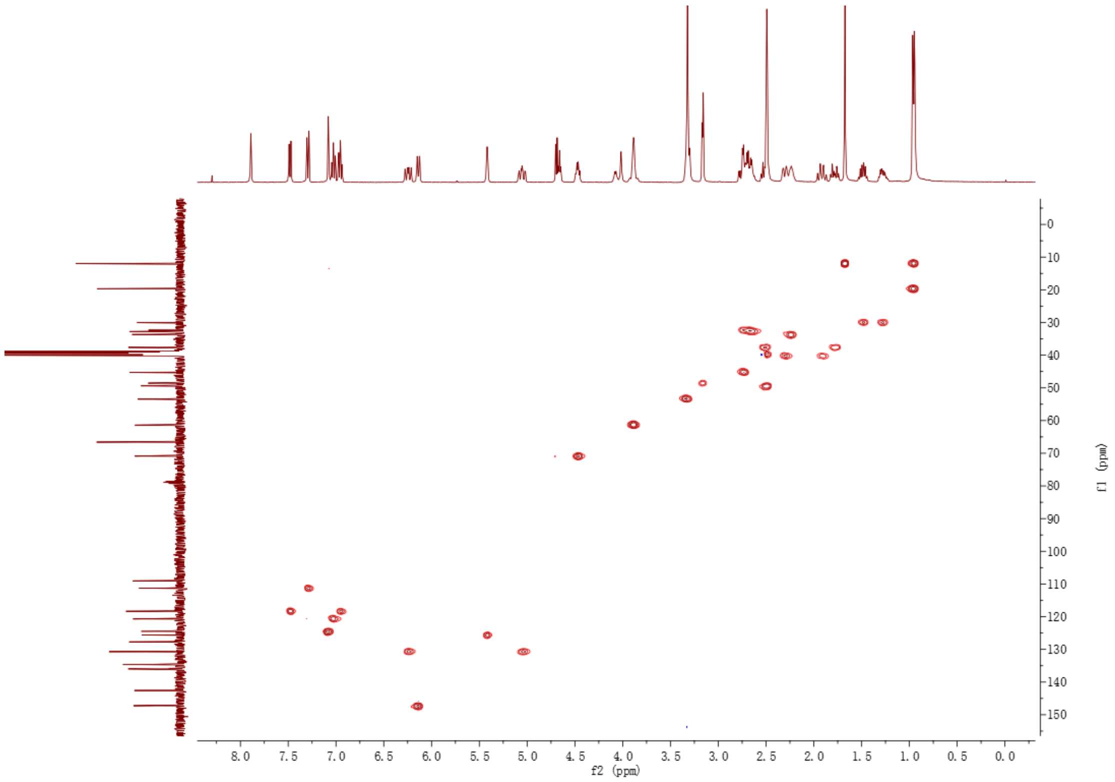

HMBC spectrum of compound **6**

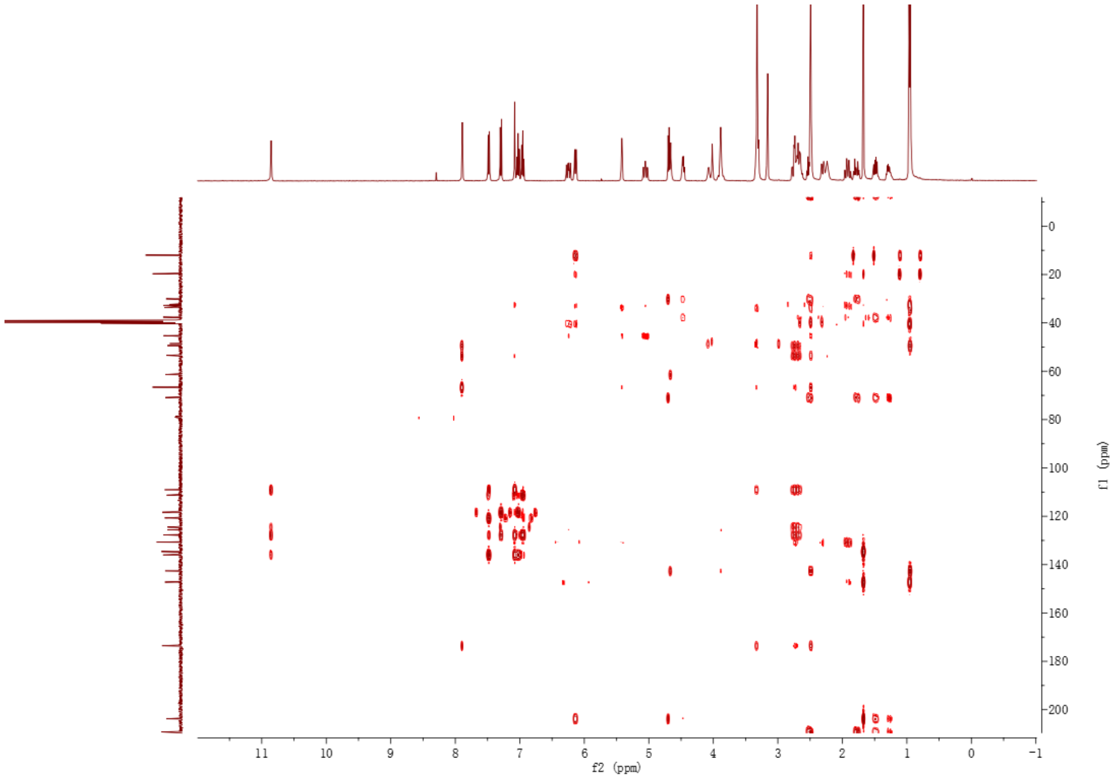

^1H–^1H COSY spectrum of compound **6**

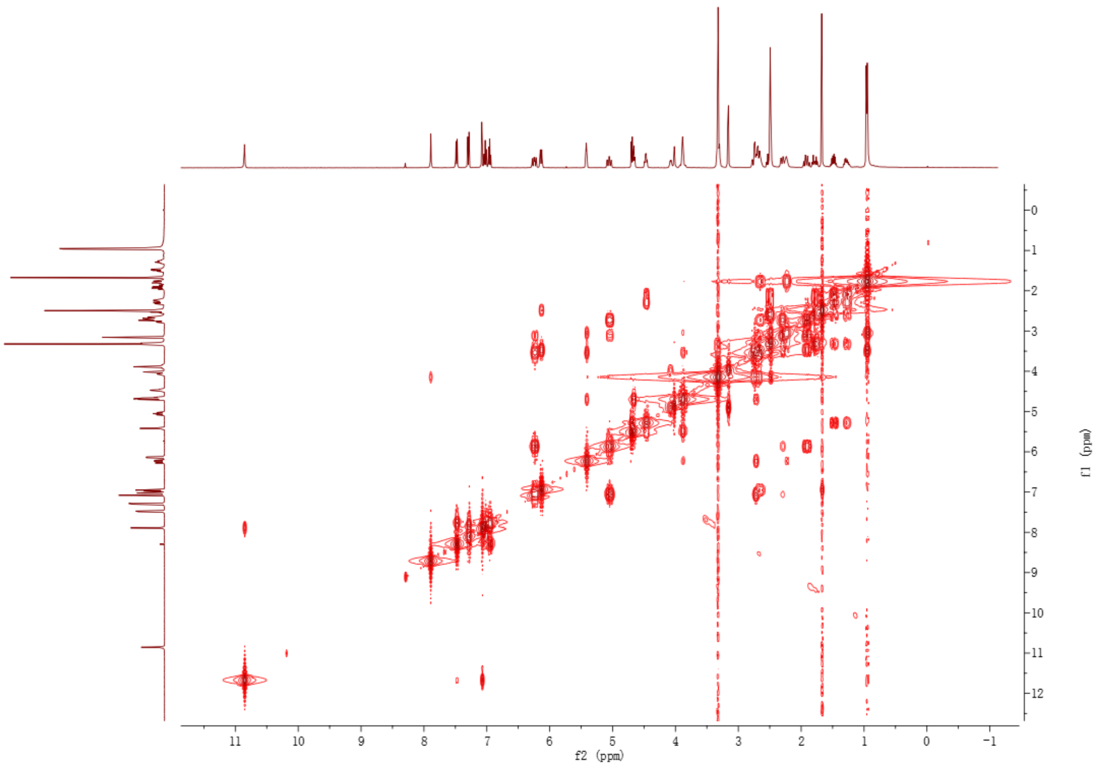

NOESY spectrum of compound **6**

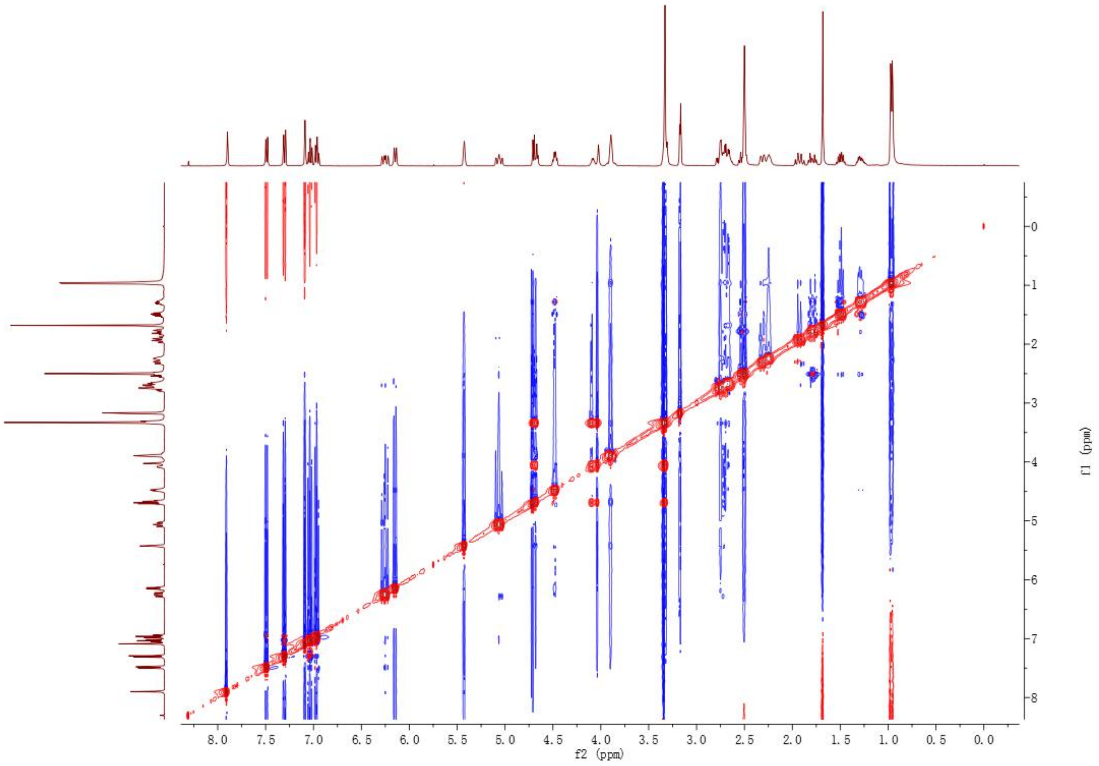

UV spectrum of compound **7**

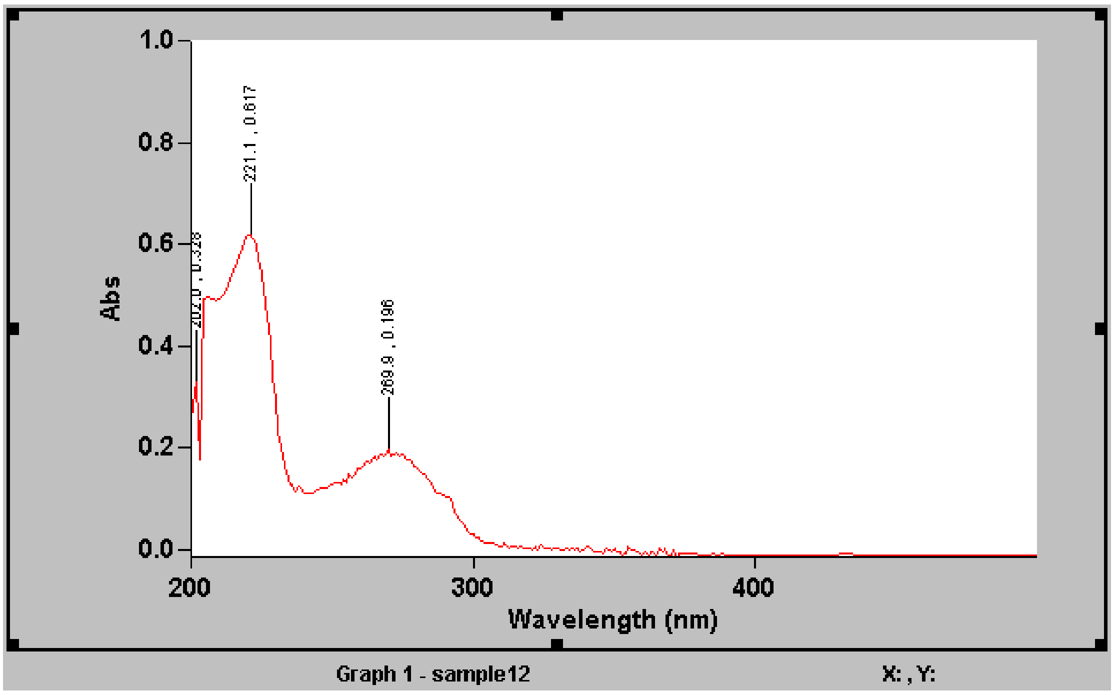

IR spectrum of compound **7**

E:\同济医学院\张勇慧\20140617\001.9

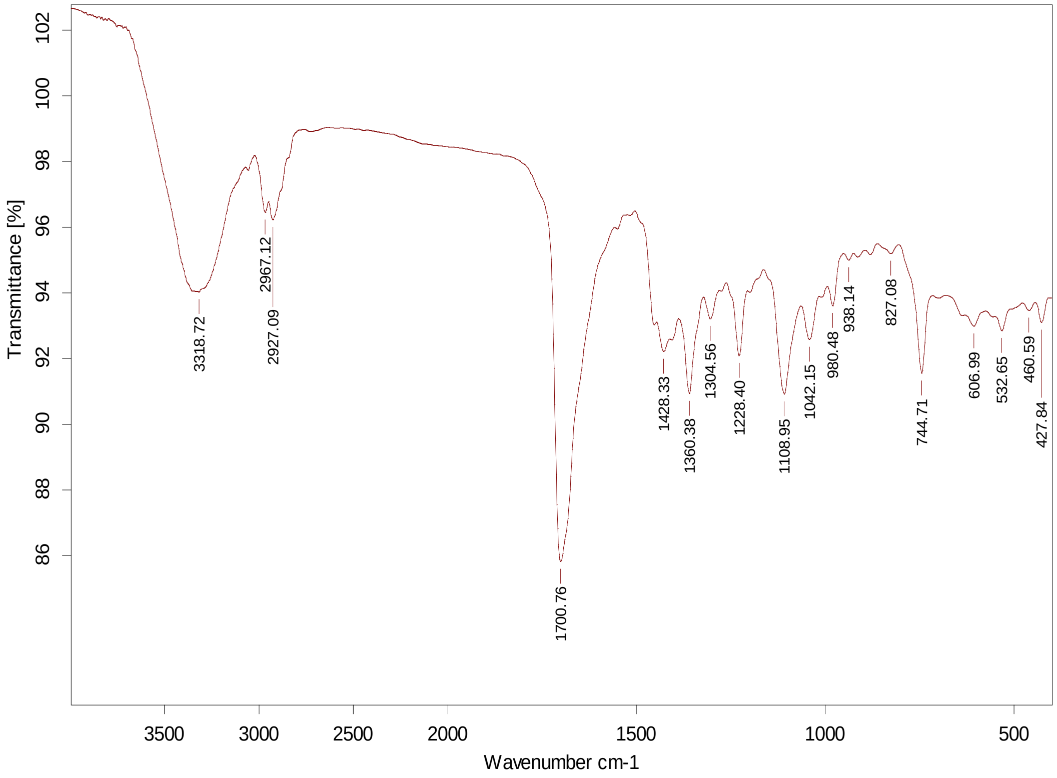

HRESIMS spectrum of compound **7**

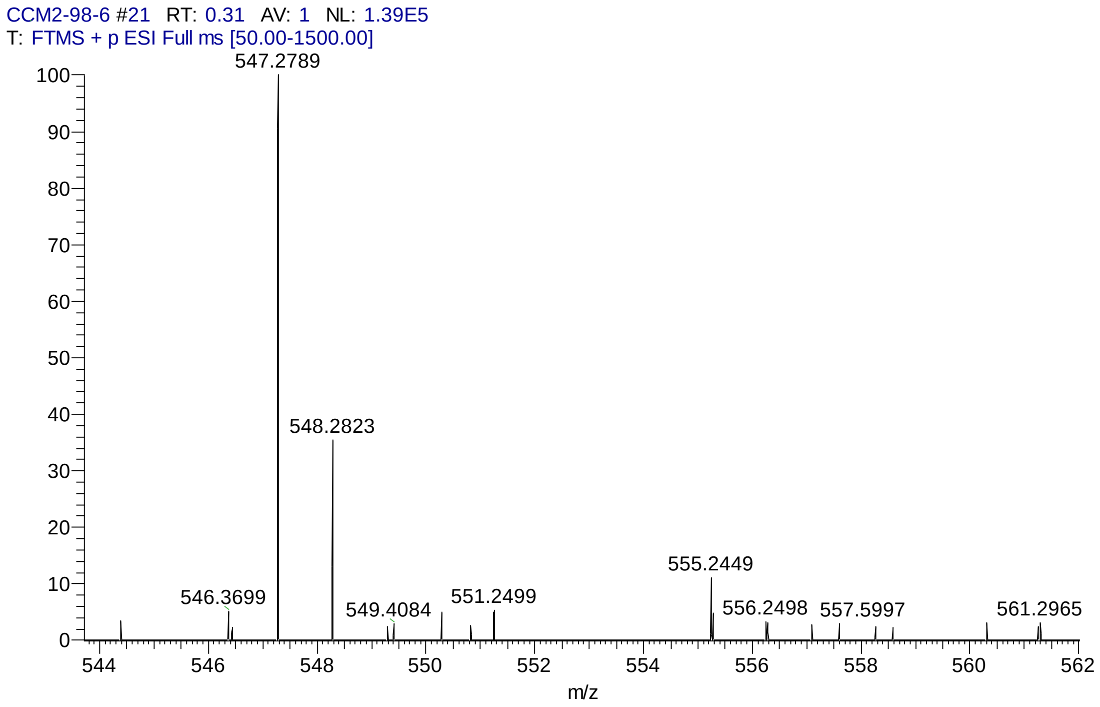

^{1}H NMR spectrum of compound **7**

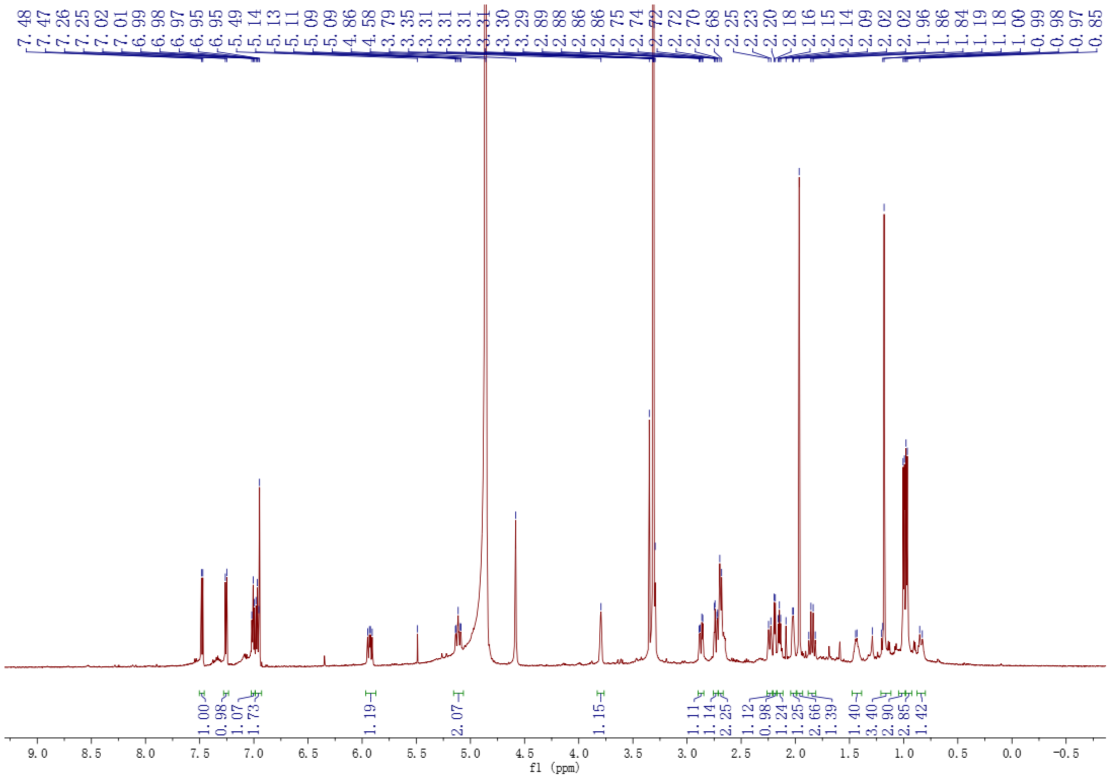

^{13}C NMR and DEPT spectra of compound **7**

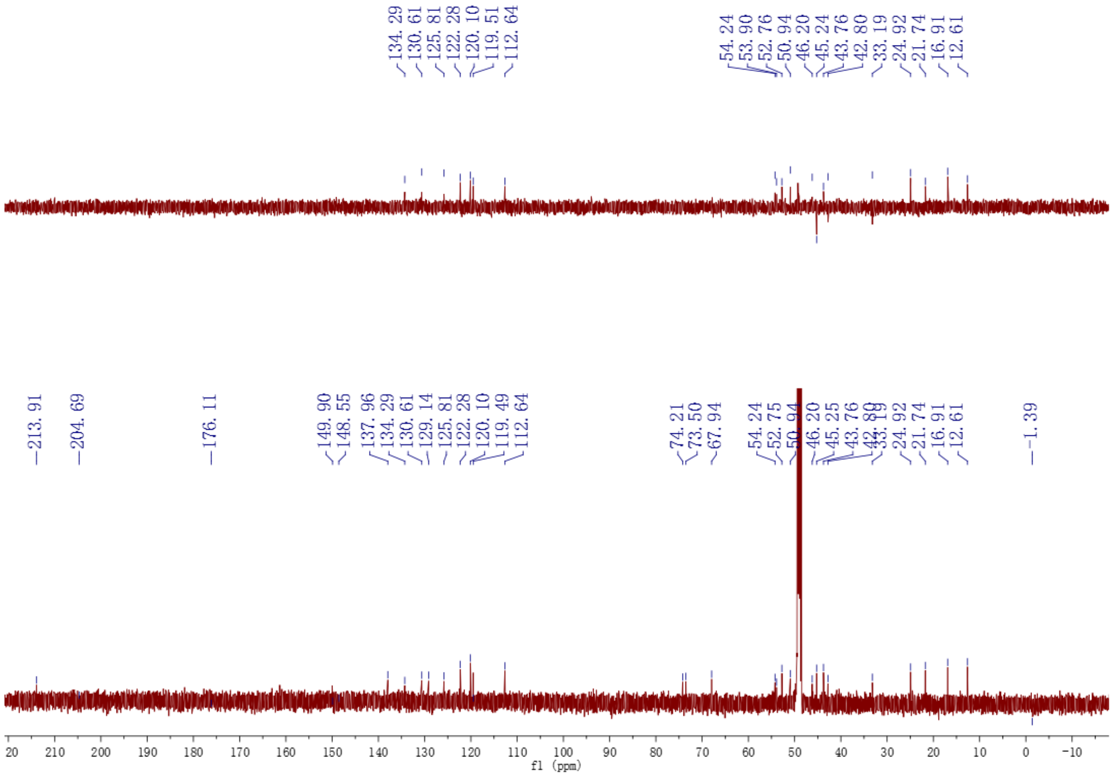

HSQC spectrum of compound **7**

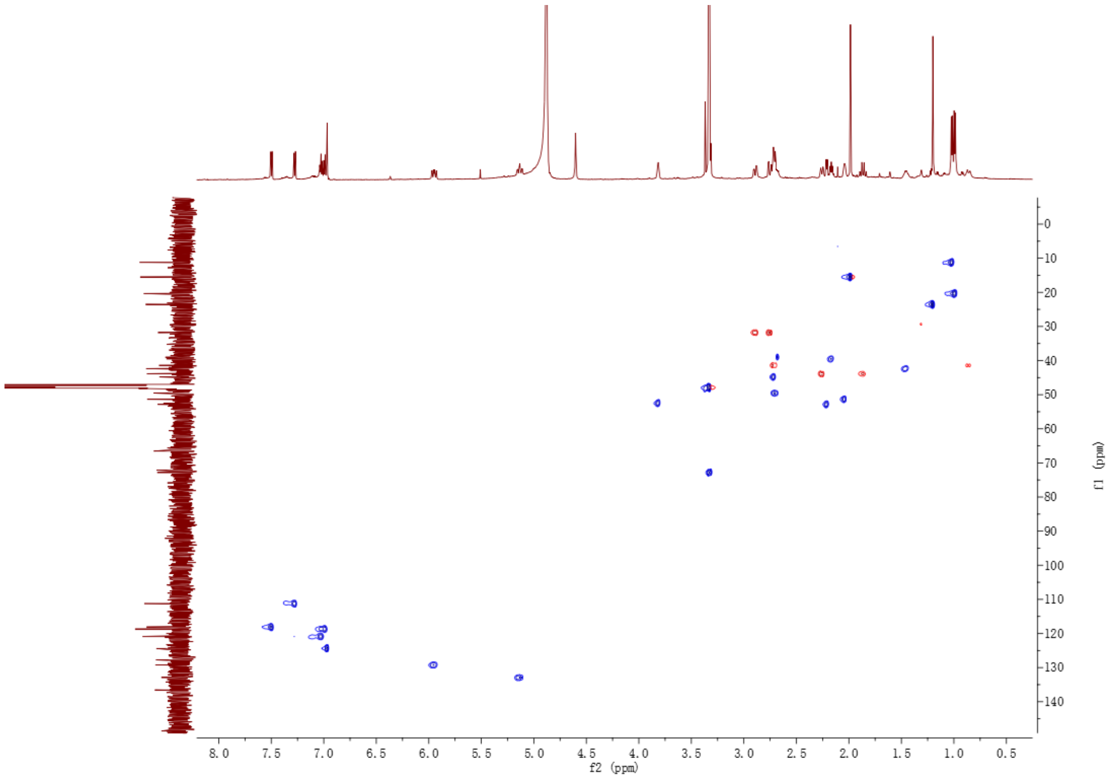

HMBC spectrum of compound **7**

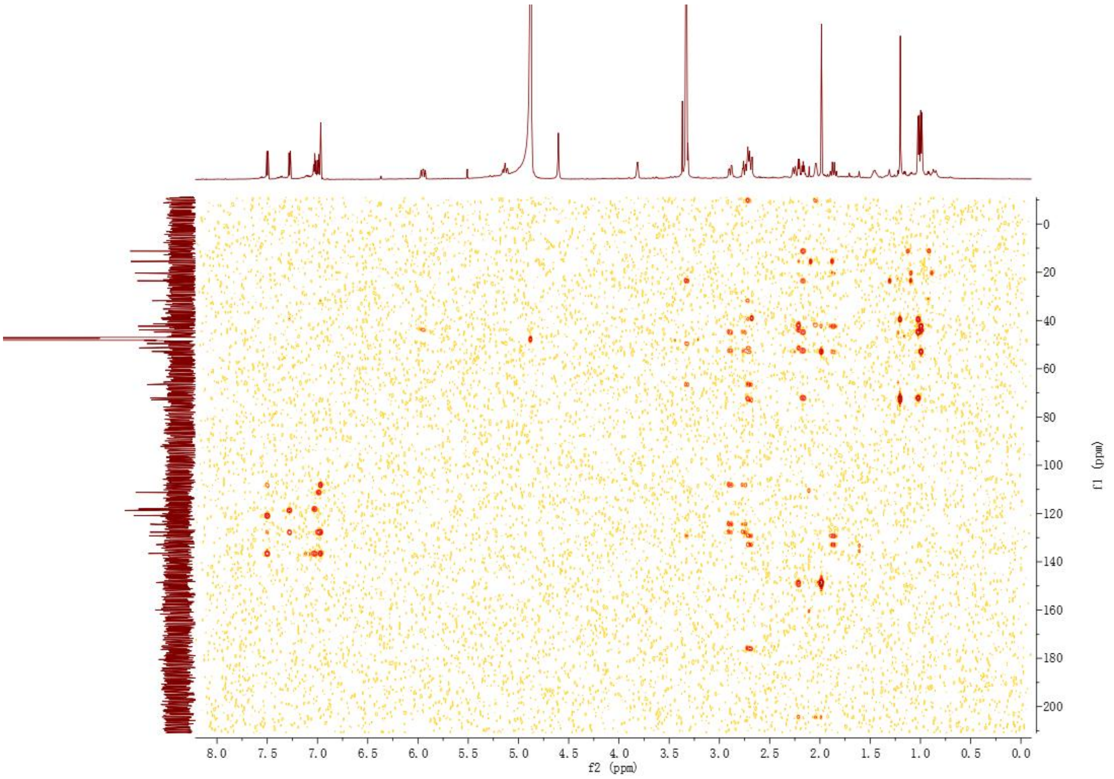

^{1}H–^{1}H COSY spectrum of compound **7**

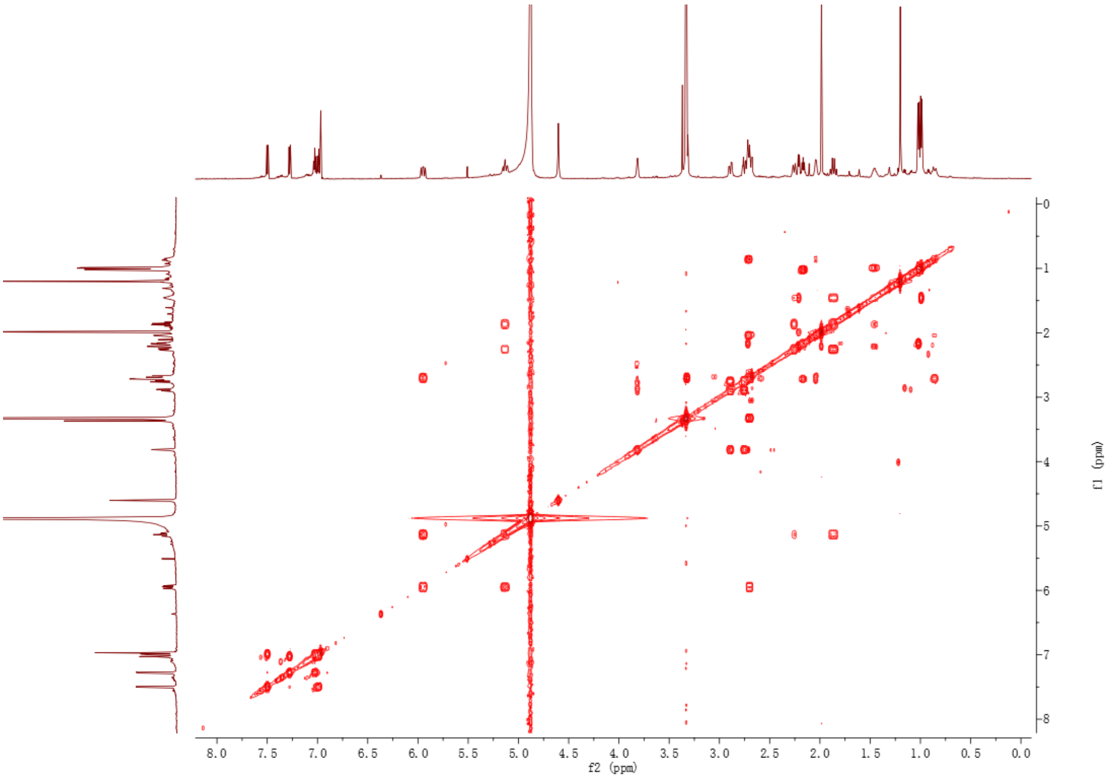

Y spectrum of compound **7**

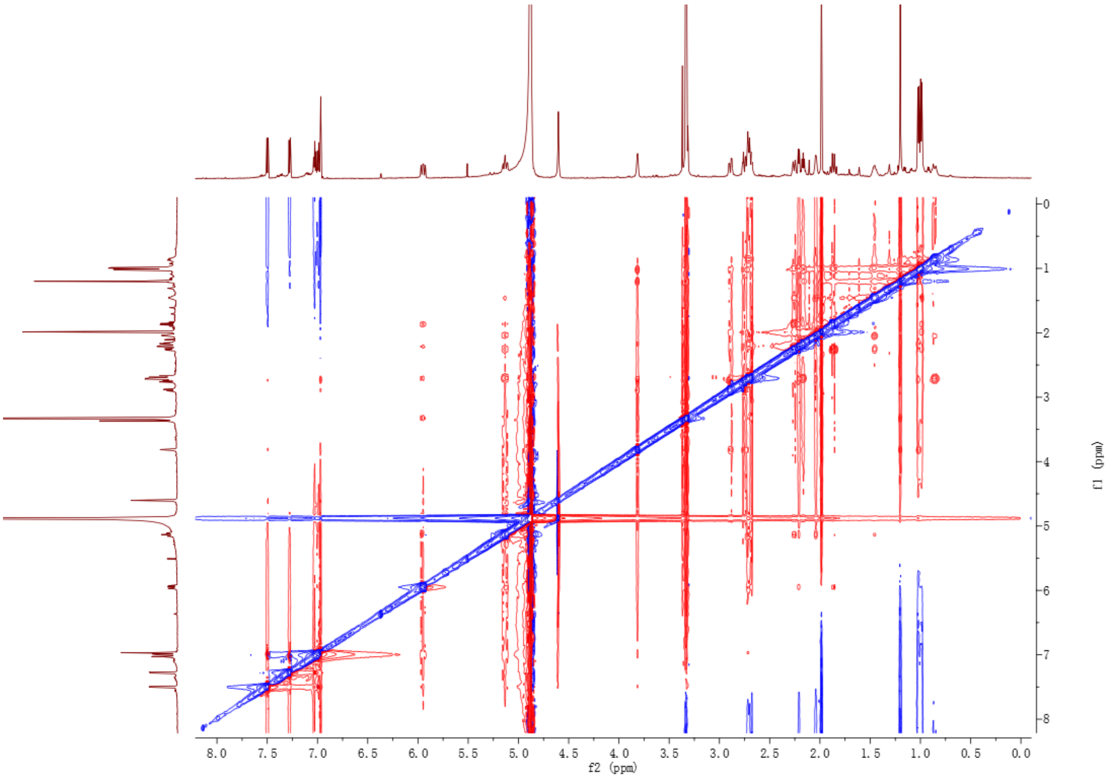

UV spectrum of compound **8**

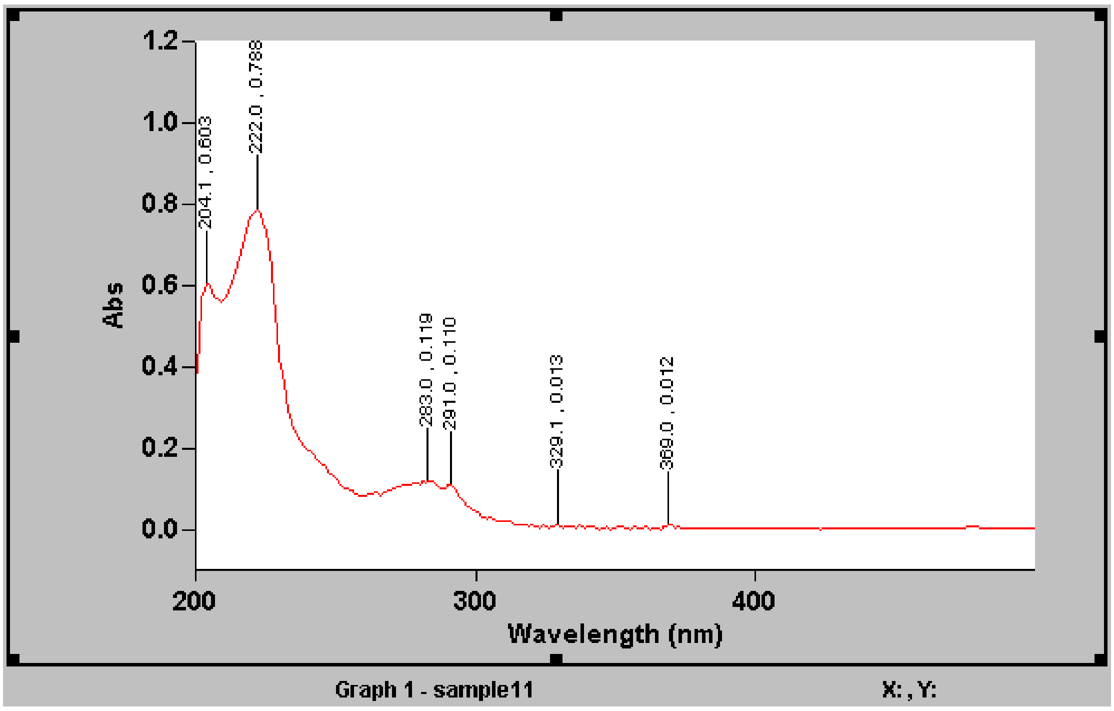

IR spectrum of compound **8**

E:\同济医学院\张勇慧\20131217\2-64-1.0

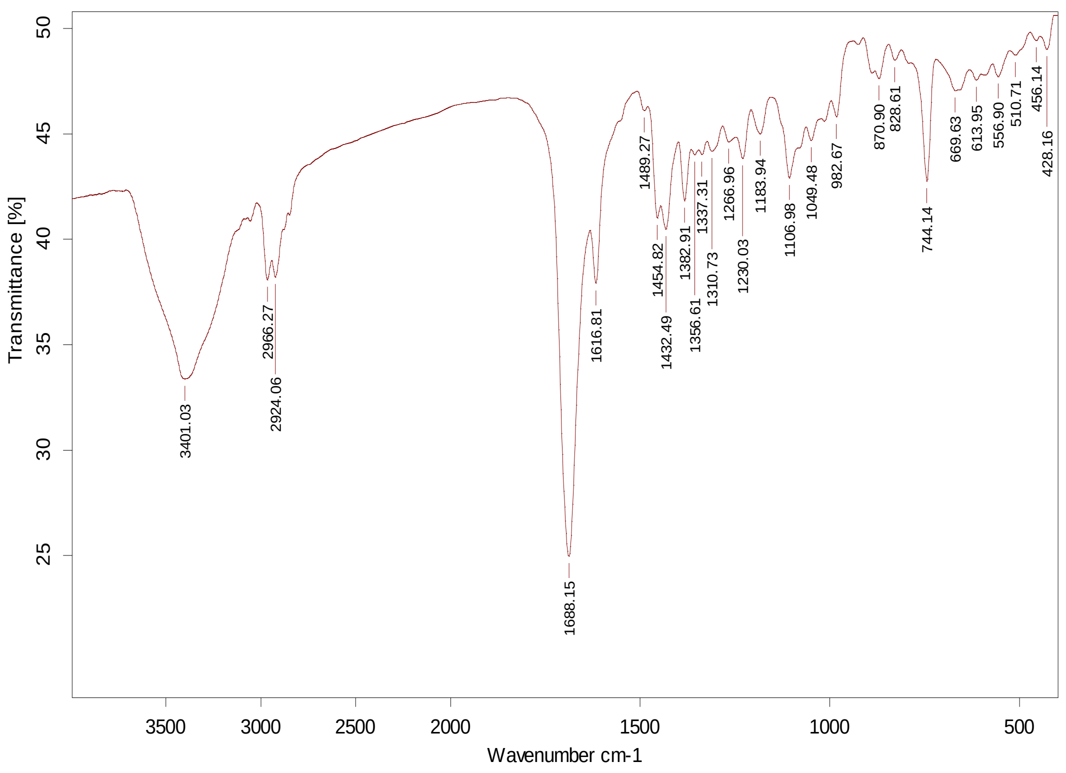

HRESIMS spectrum of compound **8**

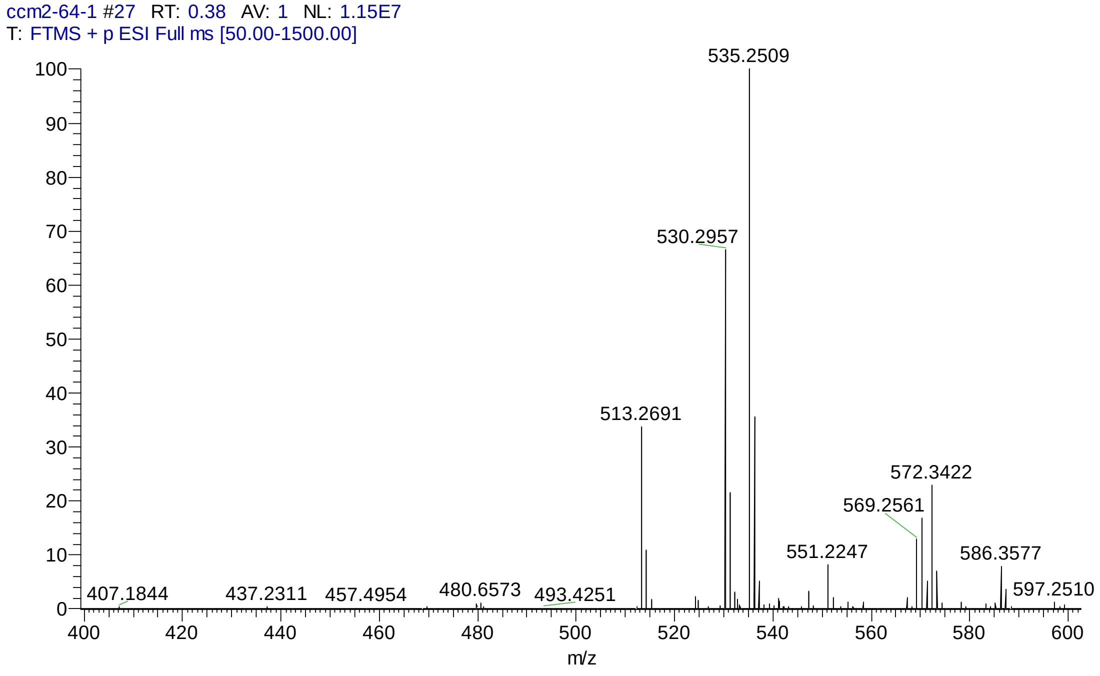

1H NMR spectrum of compound **8**

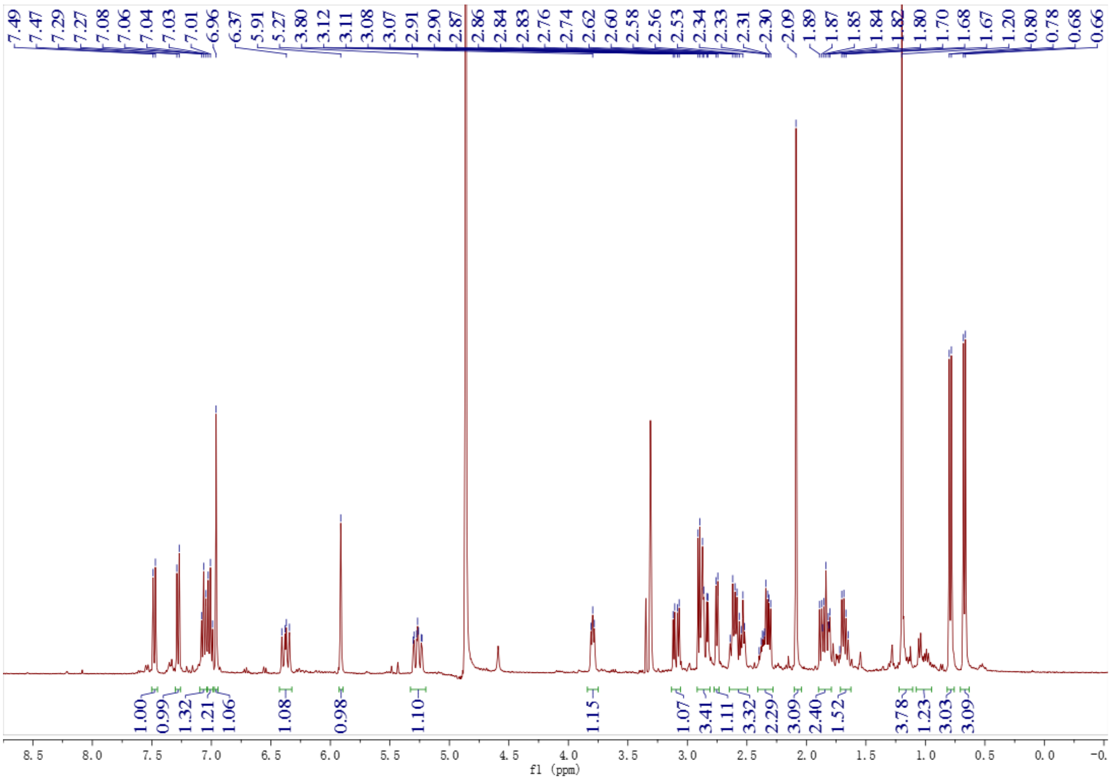

13C NMR and DEPT spectra of compound **8**

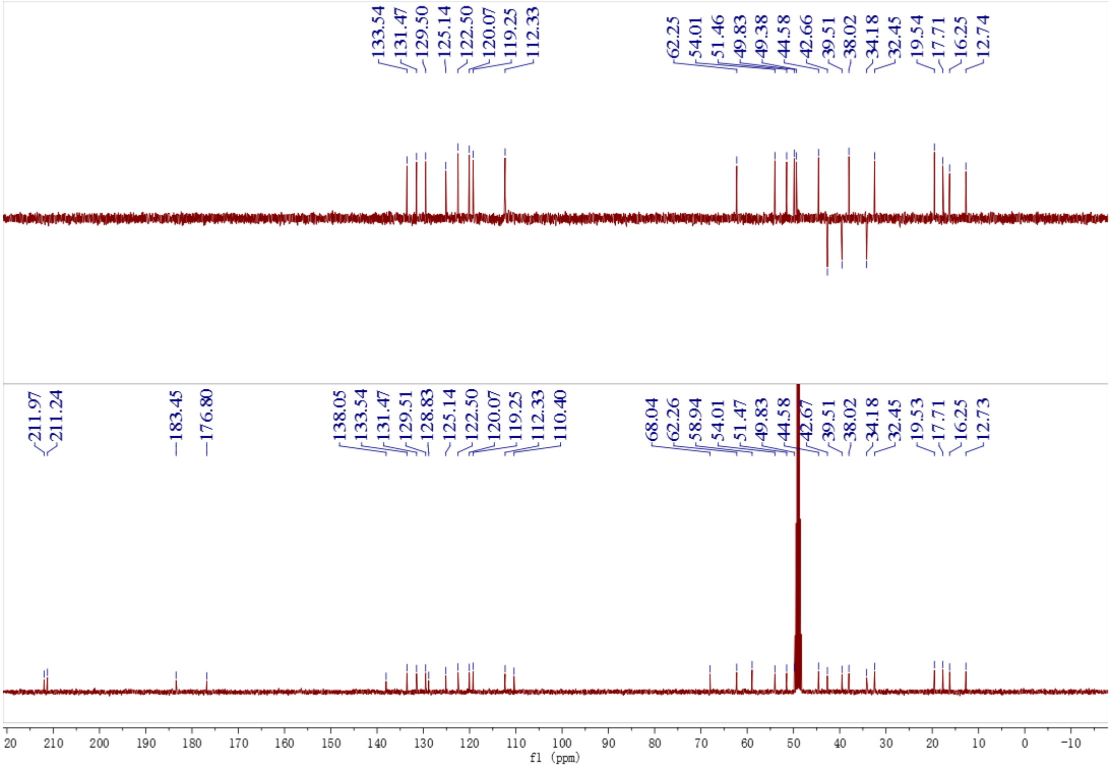

HSQC spectrum of compound **8**

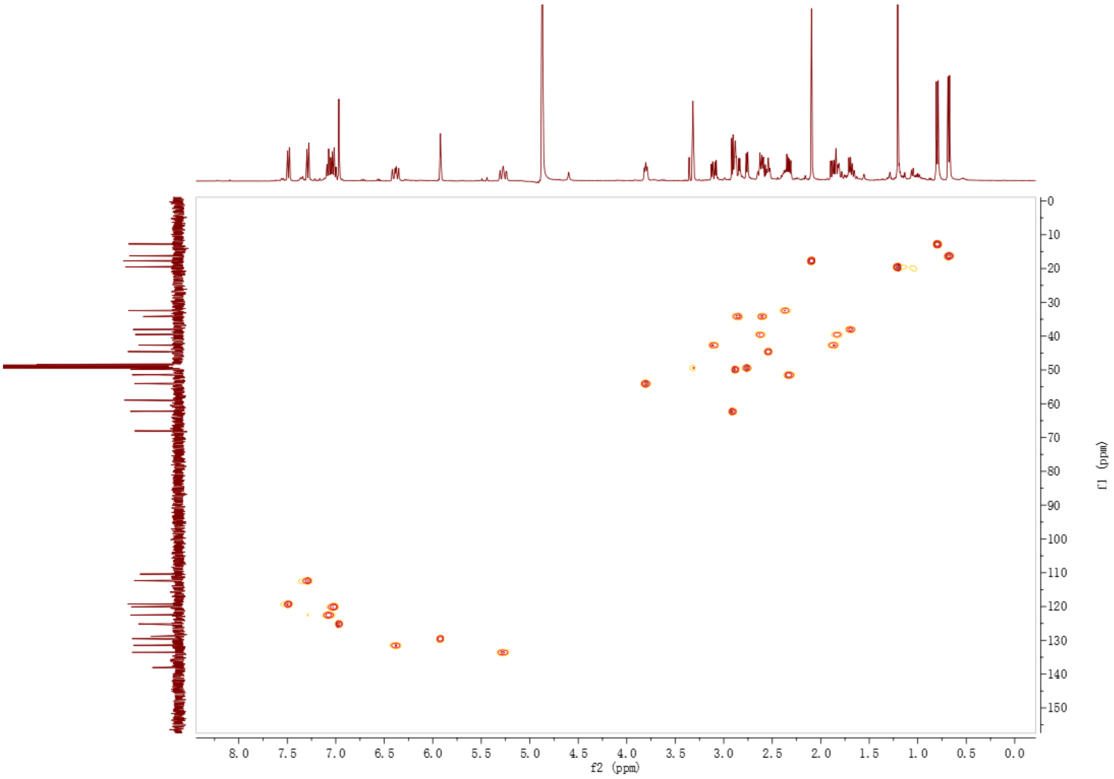

HMBC spectrum of compound **8**

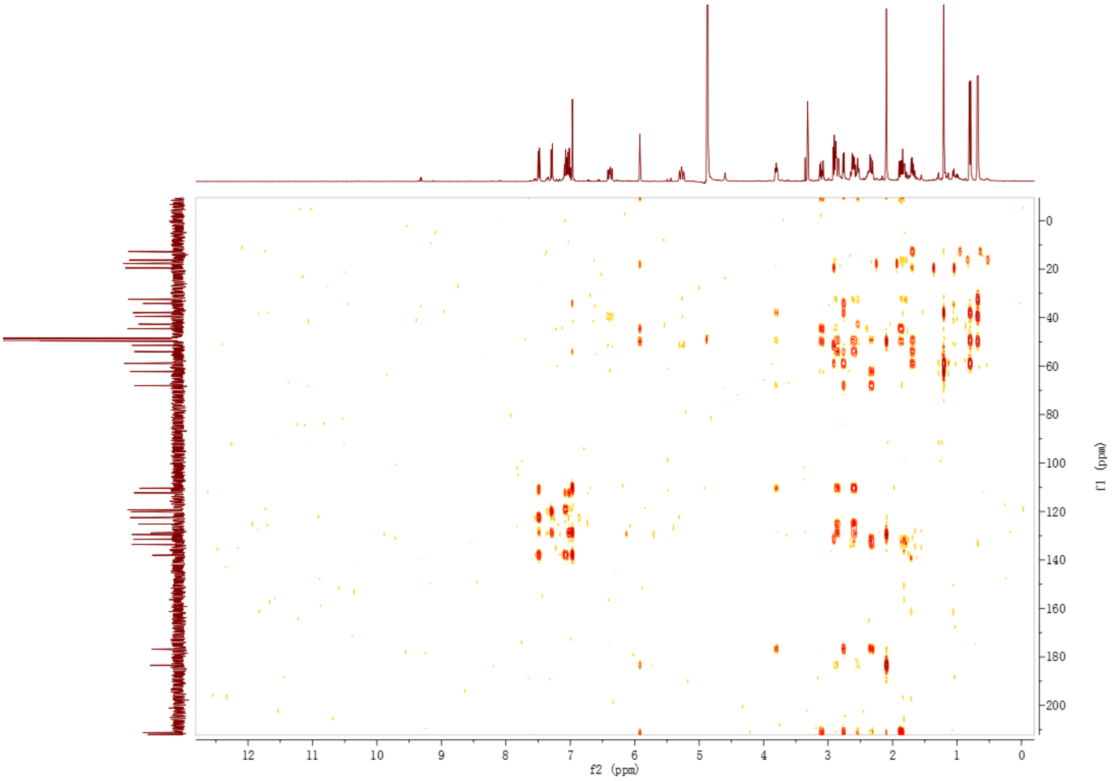

^1H–^1H COSY spectrum of compound **8**

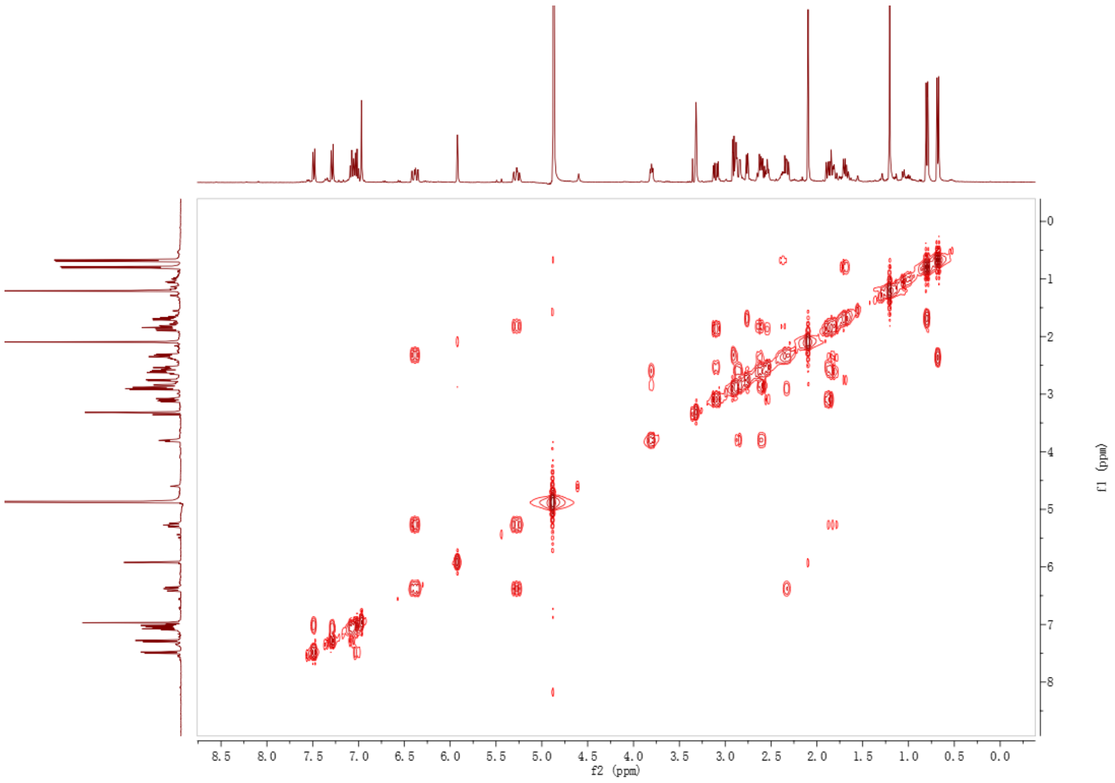

NOESY spectrum of compound **8**

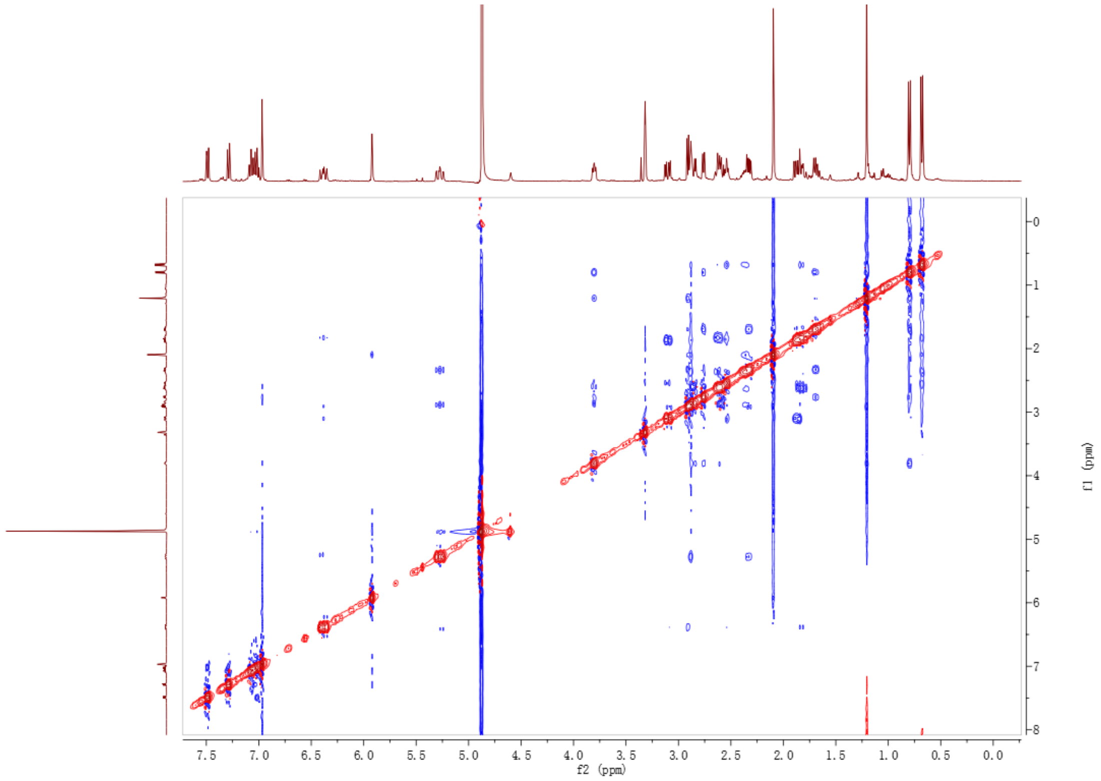

UV spectrum of compound **9**

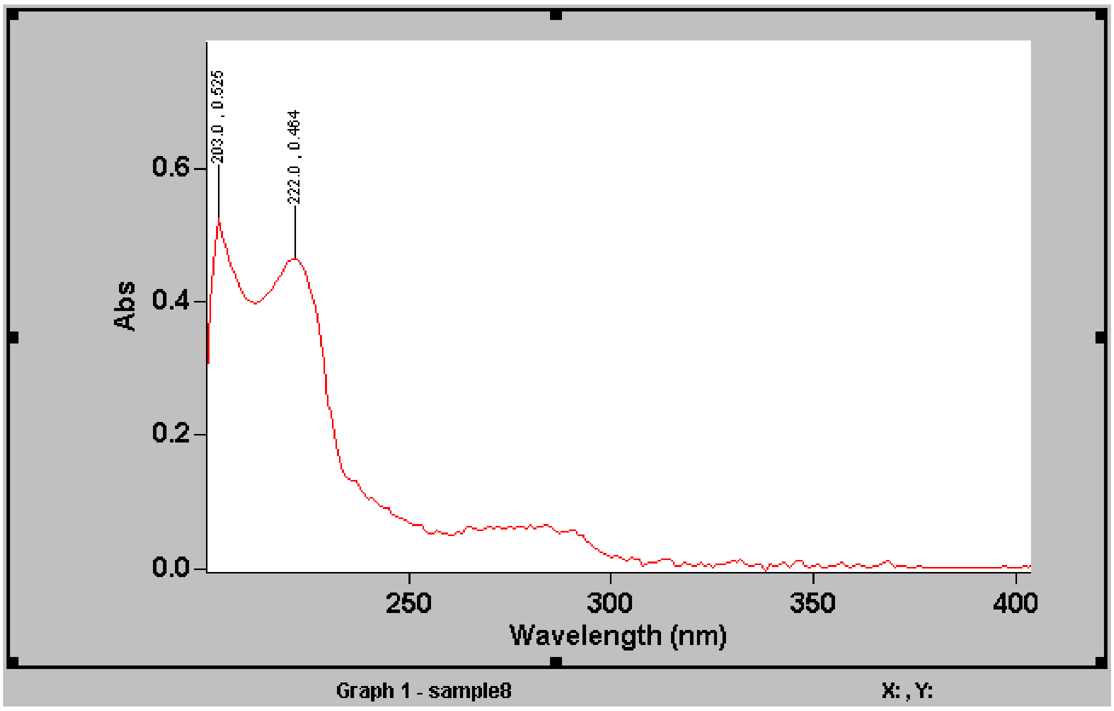

IR spectrum of compound **9**

E:\同济医学院\张勇慧\20140325\CG2-73-1.0

仪器型号：Bruker Vertex 70

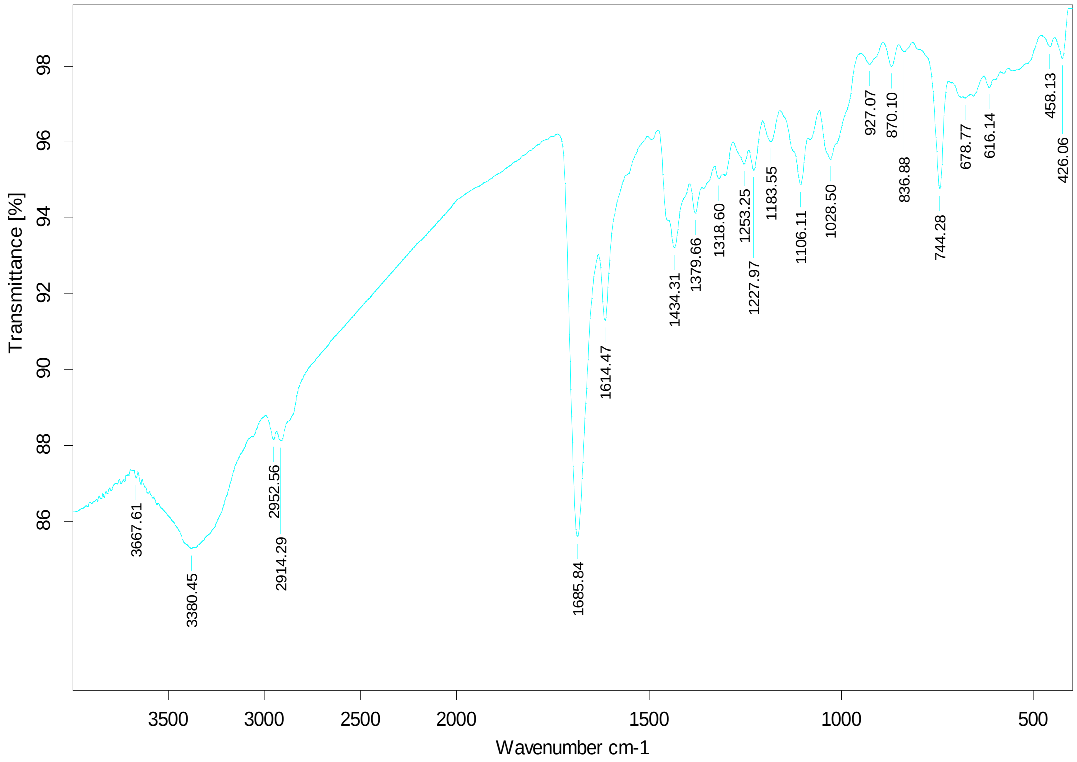

HRESIMS spectrum of compound **9**

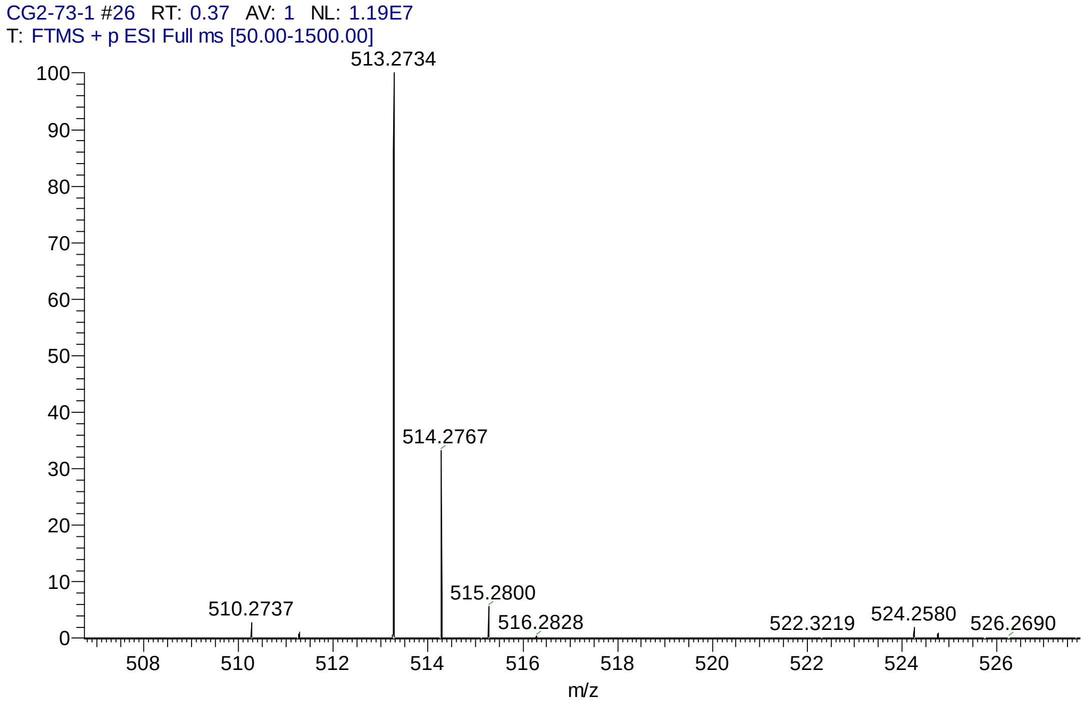

^{1}H NMR spectrum of compound **9**

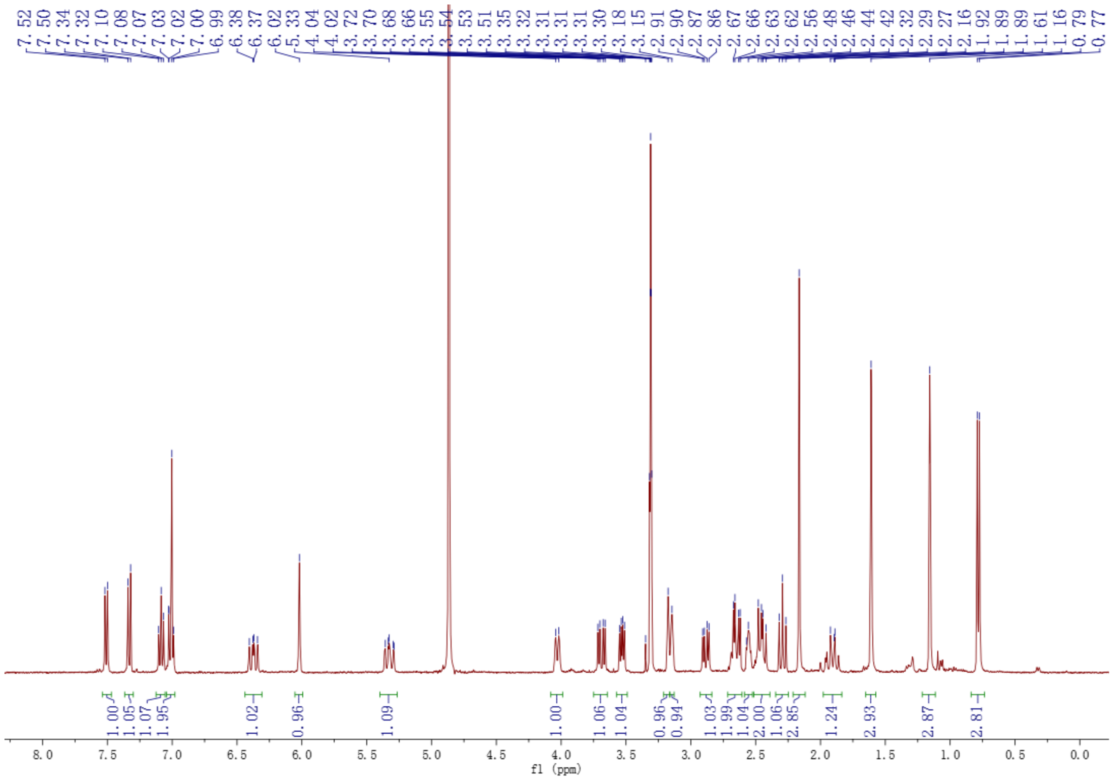

^{13}C NMR and DEPT spectra of compound **9**

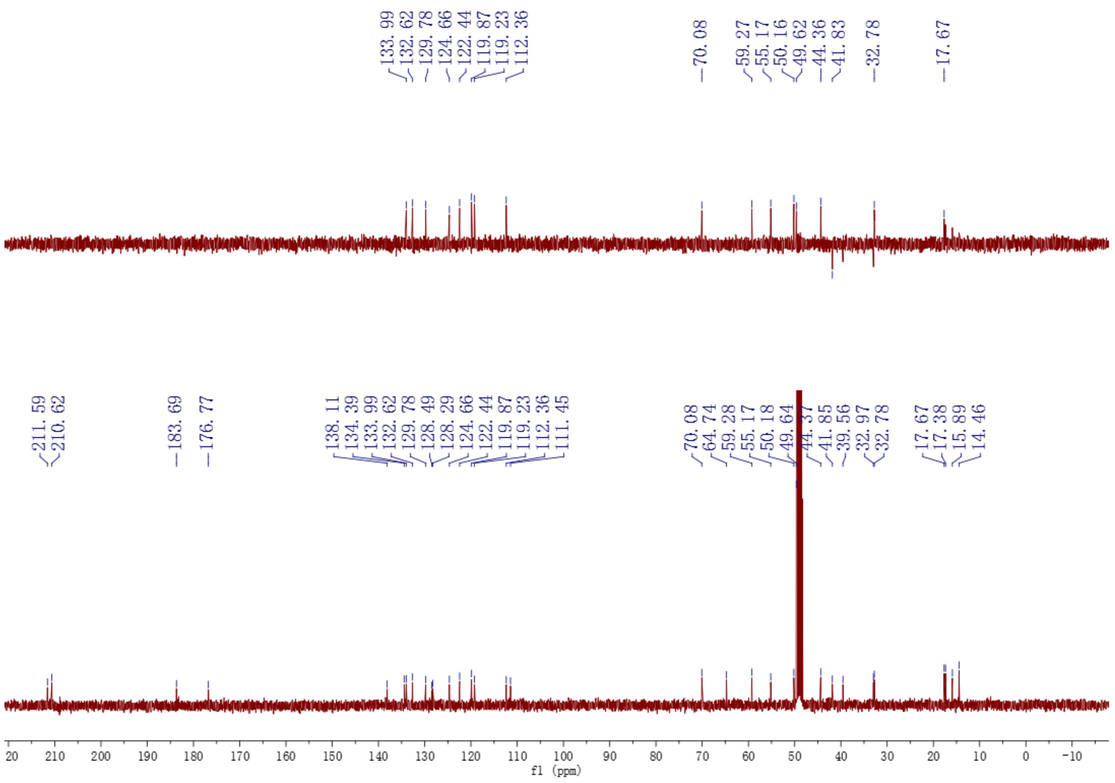

HSQC spectrum of compound **9**

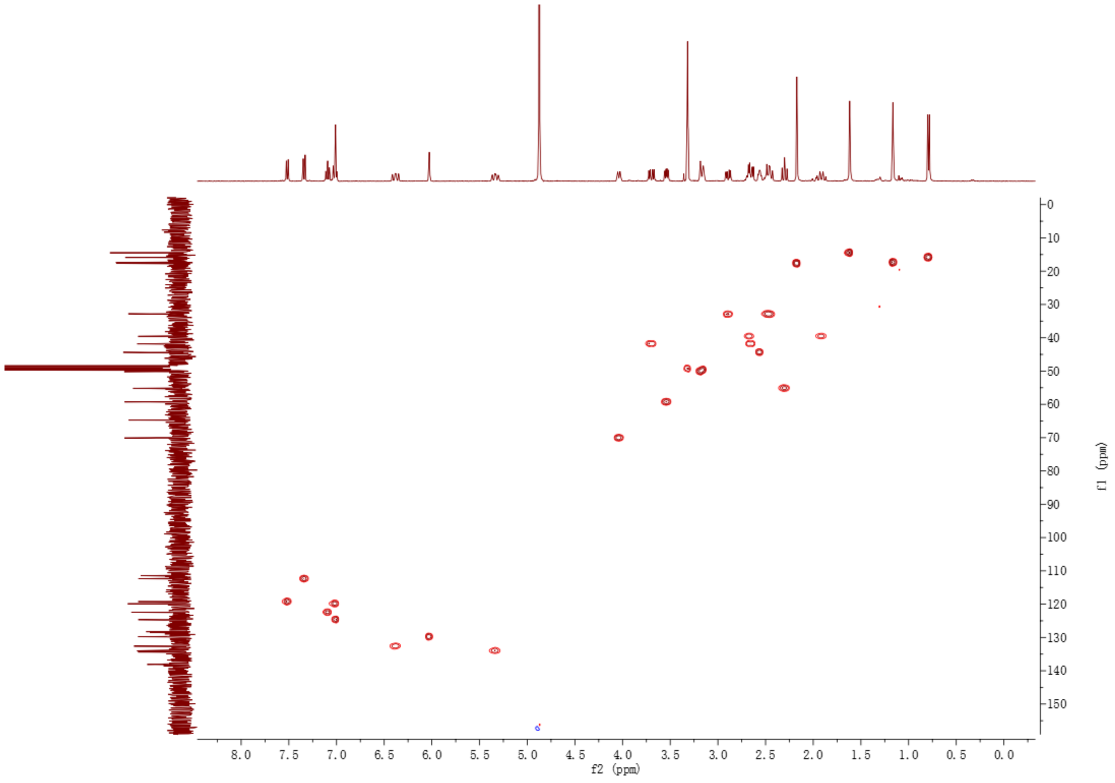

HMBC spectrum of compound **9**

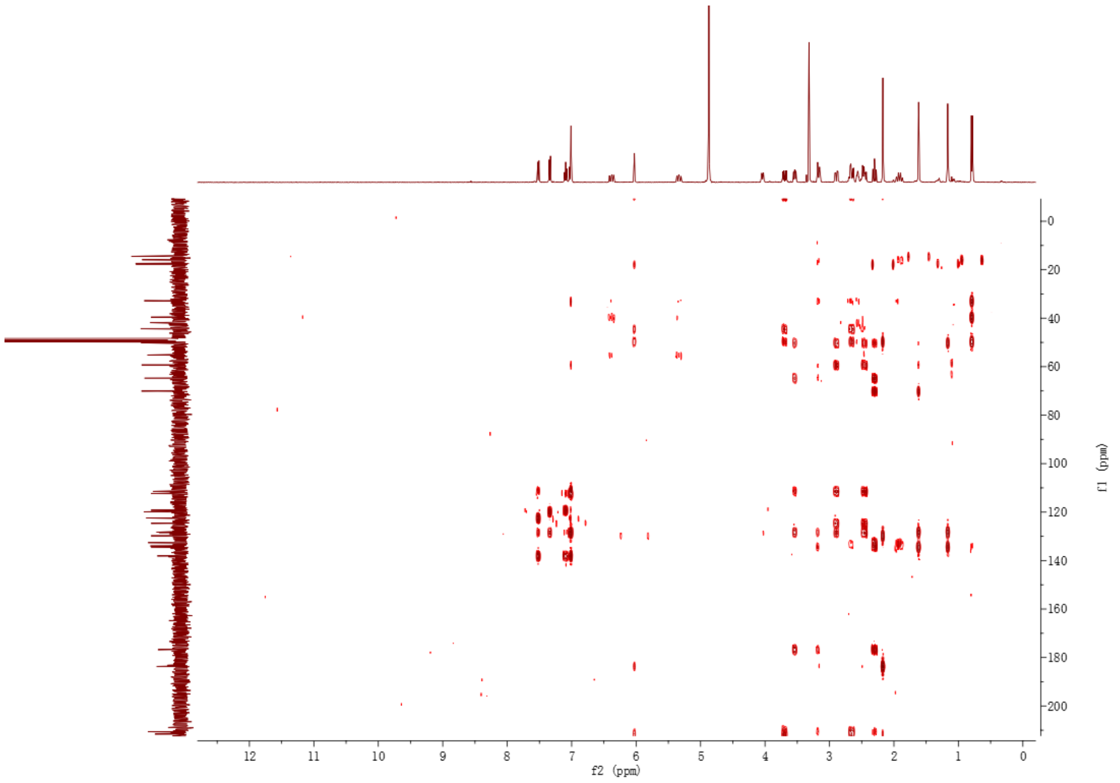

^{1}H–^{1}H COSY spectrum of compound **9**

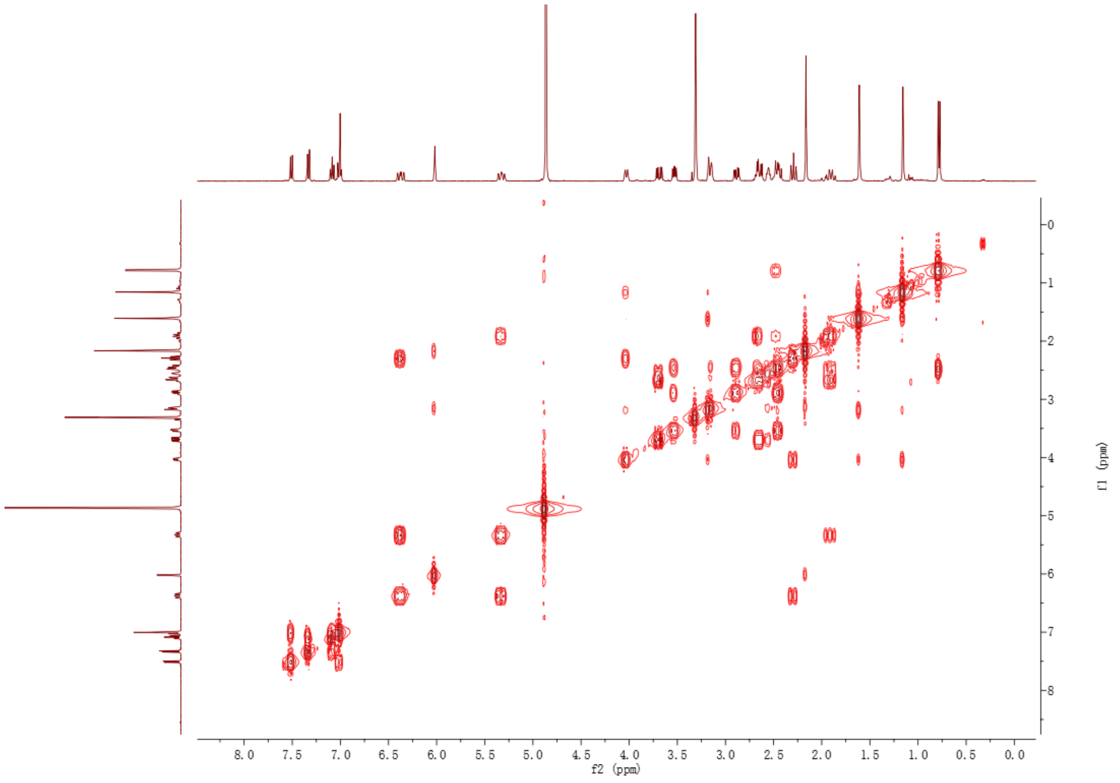

NOESY spectrum of compound **9**

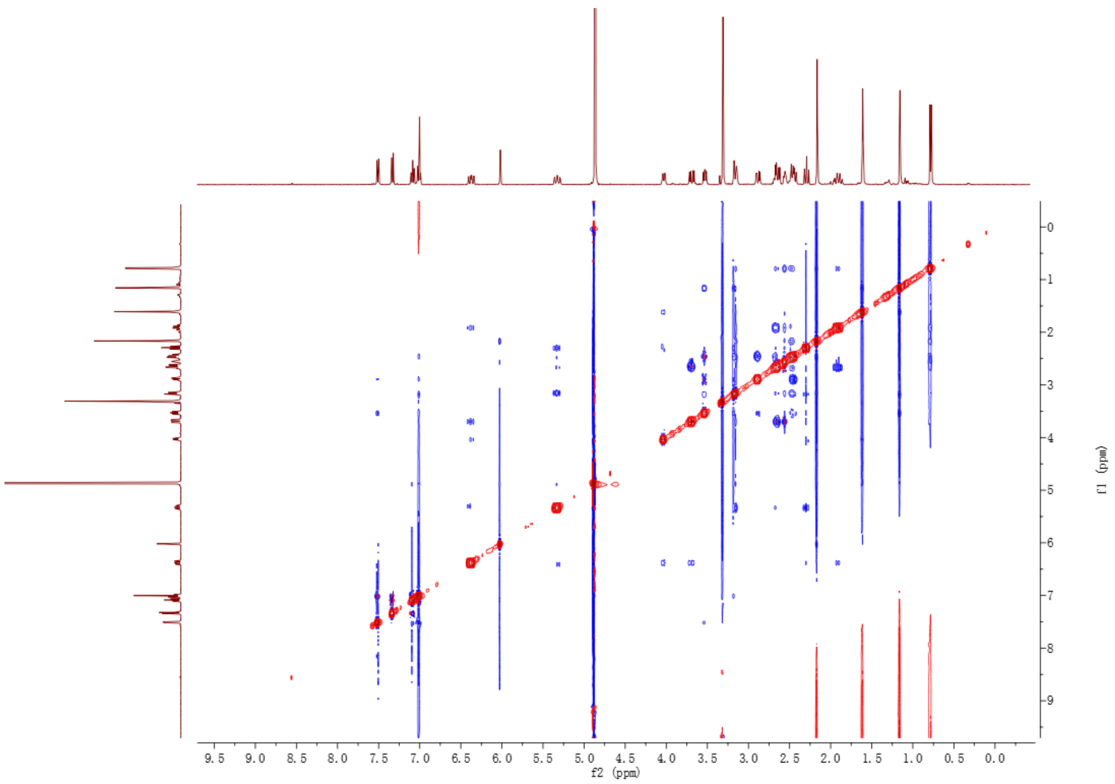

Supplement: Supplementary Information [file srep18711-s1.pdf]
